# Integrated Epigenetics of Human Breast Cancer: Synoptic Investigation of Targeted Genes, MicroRNAs and Proteins upon Demethylation Treatment

**DOI:** 10.1371/journal.pone.0027355

**Published:** 2011-11-04

**Authors:** Ramin Radpour, Zeinab Barekati, Corina Kohler, Martin M. Schumacher, Thomas Grussenmeyer, Paul Jenoe, Nicole Hartmann, Suzette Moes, Martin Letzkus, Johannes Bitzer, Ivan Lefkovits, Frank Staedtler, Xiao Yan Zhong

**Affiliations:** 1 Laboratory for Gynecological Oncology, Department of Biomedicine/Women's Hospital, University of Basel, Basel, Switzerland; 2 Biomarker Development, Novartis Institutes of BioMedical Research, Novartis Pharma AG, Basel, Switzerland; 3 Department of Biomedicine and Department of Cardiac Surgery, University Hospital Basel, Basel, Switzerland; 4 Biozentrum, University of Basel, Basel, Switzerland; 5 Department of Obstetrics and Gynecology, Women's Hospital, University of Basel, Basel, Switzerland; Ohio State University Medical Center, United States of America

## Abstract

**Background:**

The contribution of aberrant DNA methylation in silencing of tumor suppressor genes (TSGs) and microRNAs has been investigated. Since these epigenetic alterations are reversible, it became of interest to determine the effects of the 5-aza-2′-deoxycytidine (DAC) demethylation therapy in breast cancer at different molecular levels.

**Methods and Findings:**

Here we investigate a synoptic model to predict complete DAC treatment effects at the level of genes, microRNAs and proteins for several human breast cancer lines. The present study assessed an effective treatment dosage based on the cell viability, cytotoxicity, apoptosis and methylation assays for the investigated cell lines. A highly aggressive and a non-aggressive cell line were investigated using omics approaches such as MALDI-TOF MS, mRNA- and microRNA expression arrays, 2-D gel electrophoresis and LC-MS-MS. Complete molecular profiles including the biological interaction and possible early and late systematic stable or transient effects of the methylation inhibition were determined. Beside the activation of several epigenetically suppressed TSGs, we also showed significant dysregulation of some important oncogenes, oncomiRs and oncosuppressors miRNAs as well as drug tolerance genes/miRNAs/proteins.

**Conclusions:**

In the present study, the results denote some new molecular DAC targets and pathways based on the chemical modification of DNA methylation in breast cancer. The outlined approach might prove to be useful as an epigenetic treatment model also for other human solid tumors in the management of cancer patients.

## Introduction

Aberrant DNA methylation patterns are associated with various human diseases [1] including cancer development [2]. Hypermethylation of human tumor suppressor genes (TSGs) leads to transcriptional inactivation followed by the gene silencing and carcinogenesis [3].

It was also discovered that microRNAs (miRNAs), endogenous non-coding RNAs with 19–25 nucleotides in size, play important roles in various cellular processes including cellular growth, differentiation and apoptosis [4] that contribute to cancer development and progression [5]. Moreover, emerging studies reported that miRNAs are involved in promoter DNA methylation changes [6]. DNA sequences of encoding miRNAs were also found to be a target of aberrant DNA methylation as well as protein-coding genes [7].

Genetic changes such as mutation or deletion are resulting in permanent loss of gene expression while epigenetic changes are often reversible [8], [9]. Reversal hypermethylation of silenced TSGs or miRNAs is increasingly being targeted for cancer therapy and prevention [10], [11]. Moreover, these approaches are particularly appealing because DNA methylation inhibitors are considerably less toxic in non-cancerous tissues compared to other anti-cancer drugs [12].

The 5-aza-2′-deoxycytidine (decitabine; DAC; Dacogen, Eisai, Inc.), has recently been approved by the Food and Drug Administration (FDA) for the treatment of patients with Myelodysplastic Syndromes (MDS) and leukemia [13], [14]. Since DAC is one of the nucleotide analogs that is activated via phosphorylation by cellular deoxcytidine kinase and is incorporated into the DNA, the result of this process is thought to lead to the depletion of methyltransferase activity and to demethylation of DNA [15]. Several strategies have been applied to optimize or enhance the activity of DAC as a promising agent for cancer therapy [16].

Pan-omics approaches at multiple molecular levels, after DAC treatment for solid tumors, are promising in opening new mechanistic insights in this area of cancer biology. These approaches enable to synoptically probe the transcriptome and the proteome to epigenetic changes in order to understand the complete phenotype of treated cells. Therefore we used DAC in this study as a tool to predict early and late effects of DAC on different breast cancer cell lines using pan-omics approaches.

## Materials and Methods

An optimal treatment dose screening for DAC was established based on the viability, toxicity, apoptosis and methylation alterations of candidate TSGs, for six breast cancer cell lines (MDA-MB231[17], MCF-7[18], HS578T[19], BT549[20], T47D[21] and SKBR3[22]) and a breast epithelial cell line (HB2)[23] as a control.

After finding an optimal dose, three selected cell lines (HB2, MDA-MB231 and SKBR3) were treated with the optimal dose of DAC (10^2^ nM, ∼5 days), then cells were cultured up to 10 passages at “drug holiday” condition. Simultaneous extraction of DNA, RNA, miRNA and proteins was performed according to a previously published protocols [24]. 3-dimentional omics analysis including gene expression, microRNA expression and proteomics analysis was assessed before treatment, after almost 5 days of continuous treatment and at five point follow-ups (1^st^, 3^rd^, 5^th^, 7^th^ and 10^th^ passages) at “drug holiday” condition. Differentially expressed genes, miRNAs and proteins, after treatment and at follow-up passages, were measured relative to the untreated samples. Microarray expression profiling of mRNAs and miRNAs were conducted using the Human Genome 133 Plus 2.0 GeneChips and Affymetrix GeneChipR miRNA array v1.0, respectively using protocols recommended by the manufacturer (Affymetrix). In order to avoid random fluctuations in mRNAs/miRNAs expression, after log transformation and Robust Multi-array Analysis (RMA) normalization, differentially expressed mRNAs/miRNAs were defined by applying three stringent filtering criteria (mean intensity greater than six, fold change greater than two and ANOVA test set to *P*<0.05) using Partek Genomics Suite software v6.5 (Partek Incorporated, Missouri, USA). All mRNA microarray data compiled for this study is made publicly available on GEO (http://www.ncbi.nlm.nih.gov/geo/) under accession number GSE28968 and miRNA microarray data under accession number GSE28969. To validate the microarray findings, quantitative real-time (qRT) PCR was performed for 28 candidate genes and 15 candidate miRNAs.

The proteomic profile was investigated using two-dimensional gel electrophoresis (2DE) based on the previously published method [24] and analyzed with Progenesis SameSpot software (v 4.0, NonLinear Dynamics, UK). The samples variables were expressed as mean of each replicate (SE or percentages, and were statistically analyzed by ANOVA). The cutoff level for a differentially expressed protein was defined based on an ANOVA test at significance of *P*<0.05 using the SameSpots software considering a minimum of 1.5-fold change (normalized volume). The protein spots of interest were excised from the gels and analyzed by capillary liquid chromatography tandem MS (LC-MS-MS). Protein identification was done using TurboSequest software [25]. Gene networks and canonical pathways including protein-protein interactions as well as genes-miRNAs interactions were identified using the Pathway Studio® software and ResNet® (Mammal) database). The complete materials and method is presented in Dataset S1.

## Results and Discussion

### 5-aza-2′-deoxycytidine (DAC) optimal dose-range finding

In the present study, we have first identified the optimal, effective dosage of DAC by investigating the impact of concentrations in a range of 10^1^ to 10^4^ nM on seven different breast cell lines (six breast cancers and a control cell line) based on the cell viability, cytotoxicity, apoptosis and DNA methylation.

### Quantification of cell viability, cytotoxicity and apoptosis

**Figure 1:**
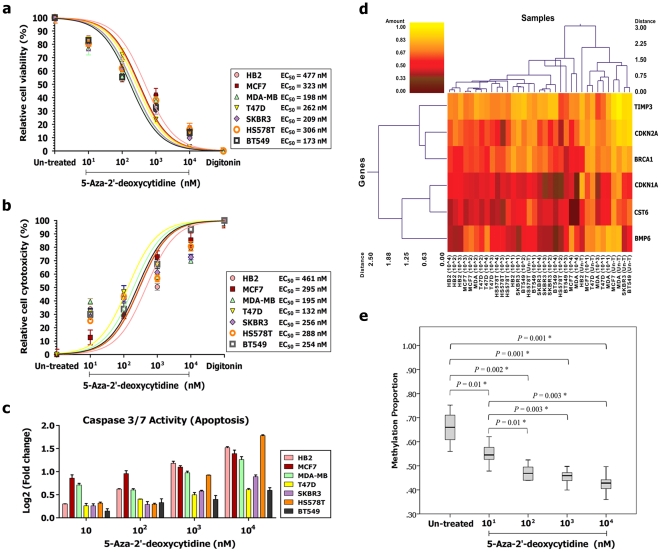
Dose response screening within six breast cancer cell lines (MCF7, MDA-MB231, T47D, SKBR3, HS578T and BT549) and an epithelial breast cell line (HB2) as a control. a-b) Multiplex quantification of the cell viability and cytotoxicity protease activities upon demethylation treatment with DAC (digitonin treated cells were considered as a cell death control). c) Quantification of caspase-3 and caspase-7 activities as a measure of apoptosis. d-e) Methylation profiling of six candidate tumor suppressor genes (red clusters indicate 0% methylated, yellow clusters indicate 100% methylated, color gradient between red and yellow indicates methylation ranging from 0-100, and black clusters indicate not analyzed CpG sites).

The MultiTox-Glo Multiplex Cytotoxicity assay measured the relative number of live and dead cells in cell populations after treatment with increasing doses of DAC and could thus determine cell viability and cytotoxicity (Fig. 1a, b). Increasing the concentration of the DAC (ranging from 10^1^ to 10^4^ nM) elevates the cytotoxicity and reduces the ratio of the cell viability in all cell lines. The results of these two measurements, cell viability and cytotoxicity, are inversely correlated (as expected). Two concentrations, 10^1^ and 10^2^ nM DAC, showed viability higher than at EC_50_ for all studied cell lines and they revealed lower cytotoxicity compared to the other concentrations.

Two cellular caspases (caspase-3 and caspase-7) play a crucial role in apoptosis and are activated in a sequential cascade of cleavages from their inactive forms. To investigate the extent of apoptosis after exposure to different concentration of DAC, the enzymatic activity of caspase-3/7 was measured (Fig. 1c). These cell death markers showed a dose dependent behavior; by increasing the DAC concentration the caspase-3/7 activities in all seven studied cell lines were elevated (Fig. 1c). The mean protease activities in the cells treated with 10^3^ and 10^4^ nM of drug were significantly higher than the lower dosages (*P* = 0.001) while the mean activities of the caspase-3/7 were similar for both 10^1^ and 10^2^ nM concentrations of DAC.

### Quantitative methylation profiling of breast cancer candidate genes

Based on our previous study where we assessed the methylation status of more than 42,528 CpG sites in 22 different genes in cancerous breast tissues versus matched normal tissues [26], we selected six hypermethylated TSGs (*BMP6*, *BRCA1*, *CST6*, *CDKN2A*, *CDKN1A* and *TIMP3*) to assess the effective DAC dose range. We analyzed the methylation proportion of these six breast cancer candidate TSGs in all seven cell lines before and after treatment at increasing doses. For all studied genes one amplicon containing CpG rich islands (number of CpG sites higher than 20) was analyzed (Table 1). In total, we assessed six amplicons, containing 171 CpG sites per sample (total of 2,394 sites in all analyzed samples) (Table 1; Fig. 1d). The mean methylation quantity of the informative CpG sites per each gene was used to assess the methylation proportion of the candidate genes (Fig. 1d). DNA methylation proportion of these six studied genes were significantly decreased for all applied concentrations in the studied cell lines (*P*<0.01) (Fig. 1d). There were no significant differences on the extent of demethylation induced by DAC at concentrations of 10^2^, 10^3^ and 10^4^ nM, whereas hypomethylation of DNA was significantly more than the lowest treatment dosage (10^1^ nM) (Fig. 1e). This data suggests that 10^2^, 10^3^ and 10^4^ nM dosages of DAC have more effective demethylation activity by significantly reducing the methylation proportion in the treated cells.

**Table 1:** High-throughput methylation analysis of CpG sites per amplicon for the 10 candidate genes.

Genes	Amplicon size (bp)	Total No. of CpG sites in the amplicon	No. of analyzed CpG sites in the amplicon	No. of analyzed CpG sites per amplicons	
				Single	Composite
*BMP6*	397	37	30	11	19
*BRCA1*	413	30	15	10	5
*CST6*	445	49	27	15	12
*CDKN2A*	580	62	36	13	23
*CDKN1A*	419	30	19	10	9
*TIMP3*	441	51	44	11	33

The *in silico* digestion was performed for T-cleavage assay. The percentage of total CpG sites in the amplicon is divided into single sites (single CpG sites) and composite sites (two or more adjacent CpG sites fall within one fragment, or when fragment masses are overlapping).

Based on the results obtained from studies of the cell viability, cytotoxicity, apoptosis and DNA methylation profiling, the concentration 10^2^ nM of DAC was identified to be the most favorable because of the less cytotoxicity and good efficiency in demethylation of DNA. This dose of DAC was chosen for the further study on the early and late effects of the treatment using pan-omics assays for the MDA-MB231 (highly aggressive) and SKBR3 (non-aggressive) cell lines together with HB2 as a control cell line. The terms “aggressive” and “non-aggressive” cell lines refer to the *in vitro* phenotype of the cell lines as indicated by a previously published paper [27].

### MRNA expression profiling

**Figure 2:**
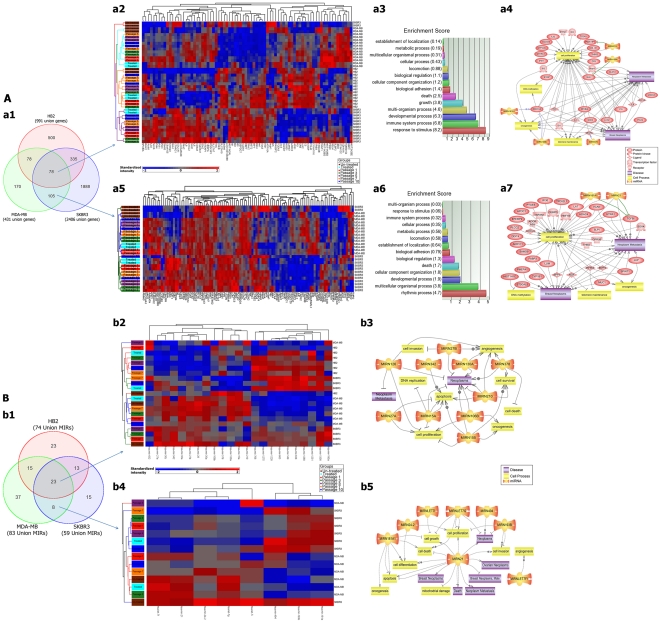
Gene and micoRNA expression matrix. A) Gene expression of HB2, MDA-MB231 and SKBR3 cell lines. a1) The total number of significant up/down-regulated genes within three analyzed cell lines. a2) Expression profiles of 78 intersection genes among all three cell lines as non-cancer specific changes. a3) Gene set analysis of 78 intersection genes. a4) Pathway analysis of 30 out of 78 genes that are linked to breast neoplasms, metastasis and/or cell proliferation. a5) Expression profiles of 105 intersection genes between MDA-MB231 and SKBR3 as cancer specific changes. a6) Gene set analysis of 105 intersection genes. a7) Pathway analysis of 36 out of 105 genes that are linked to breast neoplasms, metastasis and or cell proliferation. B) MicroRNA expression of HB2, MDA-MB231 and SKBR3 cell lines. b1) The total number of significant up/down-regulated miRNAs within three analyzed cell lines. b2) Expression profiles of 23 intersection miRNAs among all three analyzed cell lines as non-cancer specific changes. b3) Pathway analysis of 10 out of 23 miRNAs that are linked to breast neoplasms, metastasis and/or cell proliferation. b4) Expression profiles of 8 intersection miRNAs between MDA-MB231 and SKBR3 as cancer specific changes. b5) Pathway analysis of all 8 miRNAs as cancer specific changes that are linked to breast neoplasms, metastasis and or cell proliferation.

Gene expression analysis using microarray chips was assessed before treatment, after ∼5 days of treatment and at five follow-up passages at “drug holiday” condition. Dysregulated genes in at least in one of the analyzed passages of each cell line were considered as “union genes”. The common up/down-regulated genes in different cell lines were considered as “intersection genes”. Annotated lists were created for the total of 991 union genes for HB2, 431 union genes for MDA-MB231 and 2406 union genes for SKBR3 cell line (*P*<0.05, >2 fold change; Fig. 2-a1; Dataset S2 & S5). All of the significant dysregulated union or intersection genes within three analyzed cell lines were subjected to the pathway analysis in order to establish their possible role in breast neoplasms or metastasis. In parallel, K-means cluster analysis of three studied cell lines was assessed based on the Euclidean distances in order to find differences in gene expression profiles within treatment passages, while the variance provided the measure of the cluster sizes. (Dataset S2).

The expression profile of 21 candidate genes using qRT-PCR technically confirmed the results of the expression array results and also demonstrated the reliability and reproducibility of the assay using the appropriate gene identifiers [28] with the high stringency criteria as a noise filter [29].

To interpret the significance of the study, we focused on intersection genes within the two breast cancer cell lines and also on the intersection genes within all three studied cell lines. Besides that, expression profiles of known tumor suppressor genes and oncogenes were investigated based on the union lists for each cell line.

#### Intersection genes within cancer cell lines

In total, 105 intersection genes were differentially expressed in MDA-MB231 and SKBR3 cell lines (Fig. 2-a5). The gene set analysis revealed the involvement of these genes in multicellular organismal processes, especially those that are pertinent to the generation and maintenance of rhythms in the physiology of the cells (rhythmic process) (Fig. 2-a6). Pathway analyses showed that 36 out of 105 intersection genes are linked to breast neoplasms or metastasis and most of the significantly altered gene expressions in this category can potentially trigger cell proliferations (Fig. 2-a7). As an example, *CXCL3* (a small cytokine belonging to the CXC chemokine family that is also known as *GRO3* oncogene) was up-regulated after treatment in both cancer cell lines, while *JUP* (a proliferation and oncogenesis marker) was up-regulated in MDA-MB231 and down-regulated in SKBR3 (Table 2).

**Table 2:** Differentially expressed well-known cancer and/or drug tolerance related TSGs, oncogenes, miRNAs and proteins after DAC treatment in breast cancer.

3D omics	Cancer relatedgenes/miRNAs/proteins	*Up-regulated*			*Down-regulated*		
		HB2	MDA-MB231	SKBR3	HB2	MDA-MB231	SKBR3
**Gene expression** *	**Tumor suppressor genes**	*CDKN1A* [EL]*GJA1 (CX43)* [L]	*CDKN1A* [E]*CST6* [E]*PDCD4* [EL]	*CDKN1A* [E]*BRCA1* [L]*FLCN* [E]*RASSF1* [E]	*DLGAP5* [L]*FAT3* [EL]*PTEN* [L]	*GJA1 (CX43)* [L]	*GJA1 (CX43)* [EL]*CADM1* [EL]*CDKN1B* [EL]*ING4* [EL]*STEAP3* [EL]*PDCD4* [EL]
	**Oncogenes**	*CXCL1* [EL]*CXCL2* [L]*ETS1* [L]*ETV7* [E]*LCN2* [L]*MAFF* [EL]*MAP3K8* [EL]*PDGFB* [L]*RAB33B* [L]	*CXCL1* [EL]*CXCL2* [EL]*CXCL3* [EL]*JUP* [E]*LCN2* [EL]*RAB31* [EL]	*BRAF* [E]*CXCL2* [EL]*CXCL3* [E]*KLF6* [EL]*LCN2* [E]*MYC* [EL]	*RAB15* [L]	*MERTK* [E]*CXCR4* [EL]	*ERBB2* [E]*JUP* [L]*VAV1* [EL]
	**Drug tolerance related genes**	*FGF2* [L]*IL6* [EL]*MDK* [E]*TNFSF10* [EL]*VEGFA* [L]	*CSF2* [EL]*IL6* [EL]	*FGF2* [E]*IL6* [EL]*MDK* [E]*RAD51* [EL]*SERPINB5* [EL]*SFN* [EL]	*SERPINB5* [L]	*COL1A1* [EL]*VEGFA* [E]	*COL18A1* [EL]*ERBB2* [E]*TGFB1* [E]*TNFSF10* [E]*VEGFA* [L]
**MiRNA expression** **	**Tumor suppressor** **(oncosuppressor)** **miRNAs**	miR-155 [EL]miR-126 [EL]	miR-193b [EL]	miR-155 [EL]miR-193b [E]miR-125b [EL]	miR-27b [EL]miR-210 [L]miR-15a [EL]miR-155 [EL]	miR-27b [EL]miR-210 [E]miR-15a [L]let-7f [L]let-7g [L]let-7i [E]miR-125a [E]miR-125b [E]miR-126 [L]	miR-27b [EL]miR-210 [EL]miR-15a [E]let-7f [EL]let-7g [EL]let-7i [EL]
	**OncomiRs**		miR-494 [E]miR-378 [L]	miR-494 [E]miR-378 [E]	miR-27a [EL]miR-106b [EL]miR-130a [L]miR-378 [EL]	miR-27a [L]miR-106b [L]miR-21 [EL]miR-24-2 [E]miR-130a [L]	miR-27a [EL]miR-106b [EL]miR-21 [L]miR-24-2 [EL]
	**Drug tolerance related miRNAs**	miR-638 [EL]miR-768 [EL]	miR-638 [EL]miR-34a [E]	miR-638 [EL]miR-768 [EL]miR-424 [E]	miR-28 [E]miR-181a [L]miR-27b [EL]	miR-28 [L]miR-181a [L]miR-27b [EL]miR-21 [EL]let-7i [E]miR-768 [L]	miR-28 [EL]miR-181a [E]miR-27b [EL]miR-21 [L]let-7i [EL]miR-17 [L]
**Protein expression** ***	**Proteins related to cell proliferation, neoplasms and angiogenesis**	Arhgdib [E]Anxa2 [E]Tgf [E]	Gstm2 [EL]Lgals7 [EL]Pebp1 [EL]Sod2 [EL]	Cbx1 [EL]			Bat1 [EL]Pdia6 [EL]Rbbp7 [L]Ywhab [EL]
	**Proteins related to cell invasion and metastasis**	P4hb [E]	Arhgdia [EL]Pebp1 [EL]Sod2 [EL]				Pdia6 [EL]Ywhaz [EL]
	**Drug tolerance related proteins**	P4hb [E]	Arhgdia [EL]Sod2 [EL]	Glo1 [EL]			Pdia6 [EL]

The complete data include both early and late up- and down-regulations, *P* values and fold changes are presented in supplementary data.

*Dataset S2 & 5;

**Dataset S3 & 5;

***Dataset S4.

[E]: Early stage change (change after treatment and within passages 1 & 3); [L]: Late stage change (change within passages 5, 7 & 10);

[EL] Early and late change (change within passages 1-10).

#### Intersection genes within all three cell lines

There were 78 intersection genes which were differentially expressed in all three cell lines (treated and follow up passages in comparison to untreated for each cell line respectively) (Fig. 2-a2; Dataset S2). Based on the gene set analysis, these genes were mostly involved in developmental, immune system processes and response to stimulus (Fig. 2-a3). Pathway analysis showed that 30 out of 78 intersection genes have roles in breast neoplasms, metastasis and/or cell proliferation (Fig. 2-a4). For example, *CDKN1A* (*P21*) upon treatment was significantly up-regulated in all three cell lines. This is of some importance, since *CDKN1A (P21)* plays a regulatory role in DNA replication, DNA damage repair and has strong tumor suppressor activity. *GJA1* (*CX43*) was up-regulated in HB2 while down-regulated in MDA-MB231 and SKBR3. This is an important finding since *GJA1* is a TSG which is associated with suppression of metastasis [30]. One explanation for *GJA1* down-regulation might be strong activation of *IL8* in cancerous cell lines that has been also reported previously as a suppressor of *GJA1* [31]. *VEGFR* was over-expressed in SKBR3 and HB2. This gene was down-regulated in the MDA-MB231. Since the *VEGFR* gene is augmenting the cell proliferation and oncogenesis, it can play an oncogenic role in carcinogenesis [32]. *IL6* was very strongly expressed in all passages of three studied cell lines as both early and late effect of DAC treatment. IL6 is a multifunctional regulator of immune and inflammatory responses, and an increase of its expression has been detected in multiple epithelial tumors including breast cancer [33]. *IL6* is involved in the regulation of the cell proliferation, survival, and metabolism. Additionally, high expression level of *IL6* is correlated with a poor clinical prognosis which can reflect its oncogenic role [34]. However, the involvement of *IL6* in cancer is still controversial, as dichotomous roles for *IL6* in both tumor-promoting and -suppressive activities have been reported [35].

#### Gene ontology (GO) enrichment analysis

**Figure 3:**
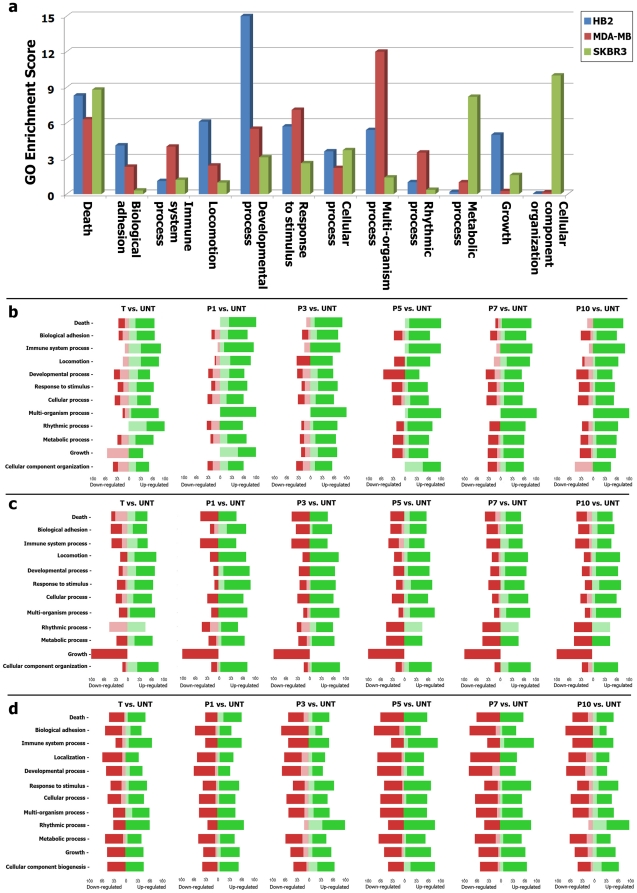
Gene ontology analysis. a) Gene set analysis and GO enrichment score for union genes of three cell lines. If a functional group had an enrichment score over 1, the functional category was over expressed. A value of 3 or higher corresponds to significant over expression (chi-square test; *P*<0.05). b) Forest plot analysis shows up/down-regulated of HB2 union genes based on their ontology enrichment. c) Forest plot analysis of MDA-MB231 union genes. d) Forest plot analysis of SKBR3 union genes.

The GO enrichment analysis was assessed for three cell lines based on the list of significantly expressed union genes in order to group them in the functional hierarchy (Fig. 3a). The enrichment scores were calculated using a chi-square test comparing the proportion of the gene list in a group to the proportion of the background in the group. Forest plot analysis showed up/down-regulated genes according to their ontology enrichment scores in each cell line, respectively (Fig. 3b).

#### Differentially expressed TSGs after DAC treatment

It is known that TSGs are hypermethylated in different tumor types as an early phenomenon during carcinogenesis [1], [36]. In the present study, expression status of well-known TSGs was analyzed after treatment and follow-up passages (Dataset S2). Up-regulation of epigenetically silenced TSGs is considered as a positive effect of DAC treatment. Several TSGs (e.g. *CDKN1A*, *PTEN*, *CST6 BRCA1* and *RASSF1*) were differentially expressed after treatment (Table 2). In HB2 cell line, most of the up/down-regulated TSGs were changed within late passages after treatment. Contrary to this, up-regulation of several TSGs in cancer cell lines was detected in early passages and remained stable till late passages (Table 2).

The *CDKN1A* (*P21*) gene showed significant hypomethylation and activation in all three studied cell lines. *CDKN1A* was previously reported as a hypermethylated TSG in breast cancerous tissue [26]. *PTEN* is an essential component of *TP53* gene that regulates p53 function through keeping Akt inactive and making Mdm2 incapable of translocation into the cell nucleus [37], [38]. Recently, we showed significant hypermethylation of *PTEN* gene in breast cancer patients lacking *TP53* mutations [39]. The epigenetic silencing of lysosomal cysteine protease inhibitor cystatin 6 (*CST6*) is more frequently observed in metastatic lesions than in primary cancers [40]. Our previous study revealed *CST6* to be significantly hypermethylated in breast tumors as compared to the matched normal tissue [26]. *CST6* was significantly reactivated after treatment in MDA-MB231. Two important TSGs, *BRCA1* and *RASSF1*, which were hypermethylated in breast tumors, [26] were significantly up-regulated in SKBR3.

#### Differentially expressed oncogenes after DAC treatment

It has been reported that some oncogenes are epigenetically hypomethylated and activated in human cancers [1]. The expression status of well-known oncogenes was analyzed after treatment and during follow-up passages (Dataset S2). Over-expression of oncogenes might be a negative feature of the treatment with DAC, while down-regulation of oncogenes could consider as positive feature of the treatment. Oncogenes such as *RAB* family genes, *ETSA*, *CXCL1*, *CXCL2*, *CXCL3*, *ERBB2*, *MAFF*, *MERTK*, *MYC* and *PDGFB* were differentially expressed after treatment (Table 2). For example, *CXCL1* and *CXCL2* are two inflammatory cytokines that have a role in cell growth, proliferation, angiogenesis and in diminishing the apoptosis; additionally they have an oncogenic role in carcinogenesis. Furthermore, depletion of *CXCL1* and *CXCL2* expressions can inhibit metastasis [41]. In total, the dysregulation of most important oncogenes within the analyzed passages of three cell lines could be detected in both early and late passages after treatment (Table 2).

### MicroRNA expression profiling

To seek specific miRNA profile after treatment with DAC, the expression analysis was assessed before treatment, after ∼5 days of treatment and at five follow-up passages at “drug holiday” condition. Annotated lists were created of the 74, 83 and 59 miRNAs which were differentially dysregulated (*P*<0.05, >2 fold change) in HB2, MDA-MB231 and SKBR3 cell lines, respectively (Fig. 2-b1; Dataset S3 & 5). All of the significant dysregulated union or intersection miRNAs within three cell lines were introduced to the pathway analysis software to find their possible role in breast neoplasms or metastasis. Additionally, K-means cluster analysis of three studied cell lines was performed to find differences in miRNA expression profiles within treatment passages (Dataset S3).

For each cell line, expression profile of intersection and union miRNAs within the passages, and their biological relevance are presented in Dataset S3. To interpret the miRNA signatures, we focused on the intersection miRNAs within two breast cancer cell lines, the intersection miRNAs within all three studied cell lines and also differentially expressed oncosuppressors and oncomiRs based on the union lists for each cell line.

#### Intersection miRNAs within cancer cell lines

We detected eight intersection miRNAs (miR-21, -24-2, -494, -193b, -181, let-7f, let-7g and let-7i) which were differentially expressed in two cancer cell lines (Fig. 2-b4; Table 2). The pathway analysis revealed that all eight miRNAs have a role in breast neoplasms, metastasis and cell proliferation (Fig. 2-b5).

#### Intersection miRNAs within all three cell lines

In total 23 intersection miRNAs were differentially expressed in all three studied cell lines (Fig. 2-b2). Pathway analysis showed that 10 out of 23 miRNAs (miR-106b, -126, -130a, -155, 15a, -210, -27a, -27b, -342 and -378) have roles in breast neoplasms, metastasis and or cell proliferation (Fig. 2-b3).

#### Differentially expressed oncosuppressor and oncomiRs after DAC treatment

Numerous reports demonstrated that miRNAs contribute to the development and progression of cancer by acting as oncogenes or tumor suppressor genes [4], [42]. Several oncosuppressor miRNAs showed significant up/down-regulation after DAC treatment but the dysregulation of the most important ones or miRNAs were detected in both early and late passages after treatment (Table 2). This finding highlights the strong effects of the treatment on oncosuppressor miRNAs which were stable for several passages at “drug holiday” condition. As an example of up-regulated oncosuppressor miRNAs; miR-155 was over-expressed during the scheduled follow up of the treatment in both cancer cell lines while down-regulation of this miRNA has been reported in breast cancer. The TSG role for miR-155 was suggested due to decreasing cell proliferation and triggering the apoptosis [43].

As down-regulated oncosuppressor miRNAs, we can mention in both cancerous cell lines the lower-expression of two members of let-7 family, let-7f and let-7g. The expression of several members of let-7 family including let-7f and let-7g, was previously reported to be down-regulated in breast cancer samples with either lymph node metastasis or distance metastases [43]. In addition, miR-27b also showed down-regulation after treatment in all three analyzed cell lines. MiR-27b is considered as a regulator of *CYPB1* and acts as a tumor suppressor that is suppressed in breast cancer [44].

As oncomiRs, several miRNAs were differentially expressed after treatment (Table 2). Interestingly, the up-regulation of most important oncomirs was detected in early passages after treatment but down-regulation of the other oncomiRs mostly happened as a late effect (Table 2). This finding suggests that the up-regulation of oncomiRs occurred as an early response to the therapy and it changed over the late follow up passages. However, down-regulation of oncomiRs as a positive effect of the treatment was mostly found in late follow up passages and sowed more stability. These late changes might be the consequence of dysregulation of some upstream regulatory genes/miRNAs.

From oncomiR group, miR-21 has been reported as a potential oncogene which promotes cell survival and proliferation [45]. In breast cancer, miR-21 is overexpressed [43] and by targeting multiple tumor suppressor genes, such as *PTEN*, *PDCD4*, *TPM1*, and *MASPIN* has been implicated in the acquisition of invasiveness and in promoting tumor metastatic properties in breast cancer [46]. Moreover, miR-21 overexpression has been associated with an advanced clinical stage and lymph node metastasis [47]. Interestingly, miR-21 was significantly down-regulated in both MDA-MB231 and SKBR3 (*P*<0.001) after treatment. As another important example, miR-27a plays an important role in breast cancer by suppressing the expression of the transcription factors *ZBTB10*/*RINZF*, and subsequently increasing several angiogenic molecules, such as Survivin, *VEGF* and *VEGFR1* [48]. A previous report indicated over-expression of the miR-27a in breast cancer [49]. Our results demonstrated that DAC treatment can significantly suppress miR-27a expression.

### Protein expression profiling

**Figure 4:**
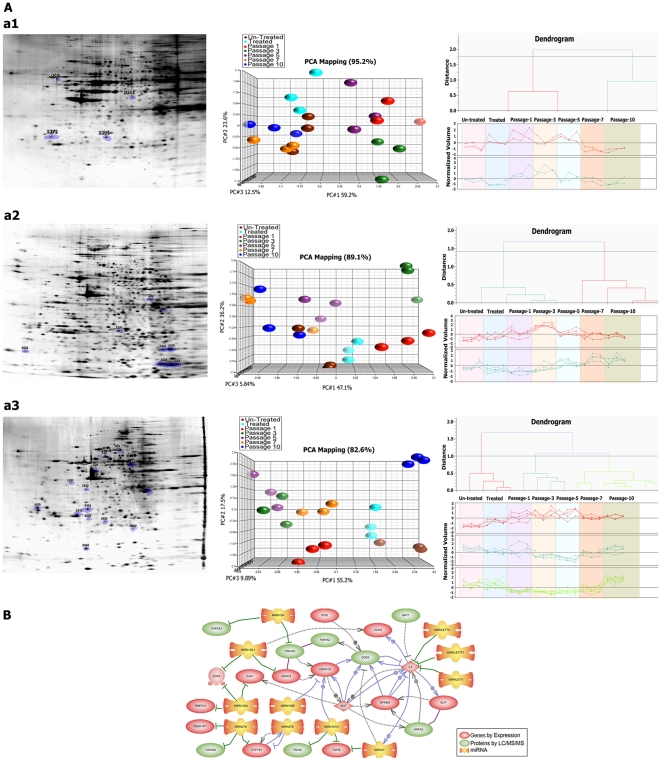
Protein expression matrix. A) Proteomics analysis of three cell lines. (a1 – a3 left panel) Proteomic images of representative gels of the three cell lines HB2, MDA-MB231 and SKBR3 with indicated spot positions excised for mass spectrometry analysis; spot picking performed from up/down-regulated intersection proteins. (a1 – a3 middle panel) PCA analysis of intersection proteins from treated cells and follow up passages 1, 3, 5, 7 and 10 from three cell lines. (a1 – a3 right panel) Clustering and classification of significantly up/down-regulated proteins based on their profiles throughout the passages. B) Crosstalk between 3-dimensional Omics. Prediction of the possible interactions and links between intersection genes, miRNAs and proteins.

Proteins from the three selected cell lines were analyzed by 2D gel electrophoresis. We could detect a total of 27 intersection significantly up/down-regulated proteins (4 in HB2, 8 in MDA-MB231 and 15 in SKBR3 cell lines) (Fig. 4A; Dataset S4). Principal component analysis (PCA) showed similarity between different treatment passages and in a dendrograms, differentially expressed proteins clustered in different groups based on their expression profile (Fig. 4A). In the control cell line (HB2), the number of differentially expressed proteins was lower than in the other two cancerous cell lines. The dendrogram of HB2 revealed two clusters and these two clusters revealed approximately the same expression level compared to the untreated cells. In MDA-MB231 (a highly aggressive cancerous line), proteins whose expression changed significantly, were categorized into two clusters. One cluster showed fluctuating up-regulation along the passages while the other returned to the level of untreated cells. Protein expression profiles in SKBR3 (non- aggressive cancerous line) were divided into three clusters. Interestingly, all three clusters ended with over-expression and are attributable to late effect of treatment. The protein expression profiles in both cancerous cell lines were related to long term effects of the treatment and could help to better define treatment intervals. The fold change, *P* value, theoretical pI and spot volumes are summarized in Dataset S4.

In total, 18 candidate spots were excised and submitted to LC-MS-MS analyses for the protein identification. Identified proteins, their gene IDs, theoretical molecular weights (kDa), number of identified peptides and the percentage of amino acid coverage are shown in Table 3. In some excised spots, we identified a single protein, in some others multiple isoforms, and yet in others we observed several proteins in a single spot. In all instances where the probability of correct determination was close to 100% (as determined by Scaffold) we have checked the observed molecular mass and pI value (according to spot position on the gel) with the MS identification.

**Table 3:** Detected proteins using capillary liquid chromatography tandem MS within three analyzed cell lines.

Cell line	No. of proteins/spot	Spot no.	Detected proteins	Gene name	Gene ID	Accession No.	MW (kDa)	No. of detected peptides	aa coverage (%)	Comments
**HB2**	Two	1385	rho GDP-dissociation inhibitor 2	ARHGDIB	397	gi|56676393	23	4	27	
			ran-specific GTPase-activating protein	RANGAP1	5905	gi|542991	23	3	15	
	Three	850	beta-tubulin	TUBB1	81027	gi|2119276	49	5	14	Replica (a)
			protein TRK-fused gene	TFG	10342	gi|21361320	43	4	8	
			protein disulfide-isomerase	P4HB	5034	gi|20070125	57	25	46	Replica (b)
	Four	1044	F-actin-capping protein subunit alpha-1F-actin-capping protein subunit alpha-2	CAPZA1CAPZA2	829830	gi|5453597gi|5453599	33	7	36	Two subunits were detected in replica (a)
			eukaryotic translation initiation factor 3 subunit I	EIF3I	8668	gi|4503513	37	9	28	Replica (b)
			zinc-alpha-2-glycoprotein	AZGP1	563	gi|4502337	34	3	7	
			annexin A2 isoform 2	ANXA2	302	gi|4757756	39	3	3	
**MDA-MB231**	One	1536	S-phase kinase-associated protein 1	SKP1	6500	gi|25777713	19	2	10	
		1622	peroxiredoxin 5	PRDX5	25824	gi|6912238	22	4	21	
	Two	953	T-complex protein 1 subunit zeta isoform aT-complex protein 1 subunit gamma isoform a	CCT7 CCT3	105747203	gi|4502643gi|63162572	58	10	19	Two subunits were detected in replica
			stress-induced-phosphoprotein 1	STI1	10963	gi|5803181	6	6	12	
		1076	voltage-dependent anion channel 2	VDAC2	7417	gi|48146045	30	7	25	
			Calponin 2	CNN2	1265	gi|4758018	34	5	17	
	Four	1293	tumor protein D54	TPD52L2	7165	gi|40805860	22	8	47	Replica (a)
			rho GDP dissociation inhibitor (GDI)	ARHGDIA	396	gi|36038	23	6		
			Galectin-7	LGALS7	3963	gi|3891470	28	3		
			glutathione S-Transferase M2-3	GSTM2	2946	gi|5822511	26	5	21	Replica (b)
		1486	manganese superoxide dismutase	SOD2	6648	gi|34707	25	5	12	
			phosphatidylethanolamine-binding protein 1	PEBP1	5037	gi|4505621	21	5	30	
			regulator of G-protein signaling 10	RGS10	6001	gi|18266777	20	4	12	
			transgelin-2	TAGLN2	8407	gi|4507357	22	3	10	
**SKBR3**	One	1121	RCN3 reticulocalbin 3	RCN3	57333	gi|28626510	7	37	21	
		1219	retinoblastoma binding protein 7 (histone-binding protein RBBP7)	RBBP7	5931	gi|4506439	48	4	7	
		1470	ribosomal protein L7	RPL7	6129	gi|35903	29	3	17	
		1543	14-3-3 protein	YWHAQYWHABYWHAZYWHAH	10971752975347533	gi|5803227gi|4507949gi|4507953gi|4507951	28	6	23	Four different isoforms were detected and confirmed in both replicates
		1590	chromobox protein homolog 1	CBX1	10951	gi|5803076	21	2	10	
		1633	glyoxalase-I	GLO1	2739	gi|5020074	21	5	22	
		2162	canopy homolog 2 isoform 1 precursor	CNPY2	10330	gi|7657176	21	2	14	
	Two	1727	GTP-binding protein SAR1a	SAR1A	56681	gi|9910542	22	3	17	
			proteasome subunit beta type-2	PSMB2	5690	gi|4506195	23	3	19	
	Four	960	spliceosome RNA helicase BAT1	BAT1	7919	gi|4758112	49	13	32	
			26S proteasome non-ATPase regulatory subunit 5	PSMD5	5711	gi|4826952	56	7	15	
			beta-tubulin	TUBB1	81027	gi|2119276	49	5	14	
			protein disulfide-isomerase A6	PDIA6	10130	gi|5031973	48	5	15	

(aa: amino acids).

Notably, in the spot 1543 (SKBR3 cell line) we could identify four different isoforms of 14-3-3 protein which were confirmed with two different replicates referring to the same spot; their calculated molecular masses or pI values deviated from the observed ones (and also from the main set of polypeptides). This highly conserved protein family, is a large family of 25–30 kDa acidic proteins and has seven homologous isoforms in mammalian cells which are involved in a variety of biological interactions, cell-cycle progression, apoptosis, and mitogenic signaling, such as the ATM-*p53* pathway, suggesting their possible role in tumorigenesis [50]. 14-3-3 theta is an adapter protein implicated in the regulation of both general and specialized signaling pathways by binding to a large number of partners, usually by recognition of a phosphoserine or phosphothreonine motif.

Interestingly, five differentially expressed genes detected by transcriptomic analysis were also identified by mass spectrometry at the proteome level, which indicated similar expression trends at both, the transcriptome and proteome (Dataset S4). The results implicated that two of these five proteins (Sod2 and Pebp1) were detected in one spot (SKBR3; 1486). These data support the mass spectrometry results for those cases where more than one protein species were detected within one single spot (Table 3).

Following the protein identification, the molecular function, biological processes, and subcellular localization was categorized using the PANTHER gene ontology database (http://pantherdb.org) and ResNet® 7 (Mammal) database. The analyzed data showed that 28 out of 41 detected proteins/isoforms in the three analyzed cell lines are linked to neoplasms, metastasis or carcinogenesis (Dataset S4).

### Integrative, pan-omics

Possible crosstalk between all identified union genes, miRNAs and proteins with a reported role to carcinogenesis, was investigated in this study. In total, 11 genes, 11 miRNAs and 10 proteins showed interesting regulatory interactions with each other (Fig. 4B).

#### Dysregulation of metastasis related genes/miRNAs

**Figure 5:**
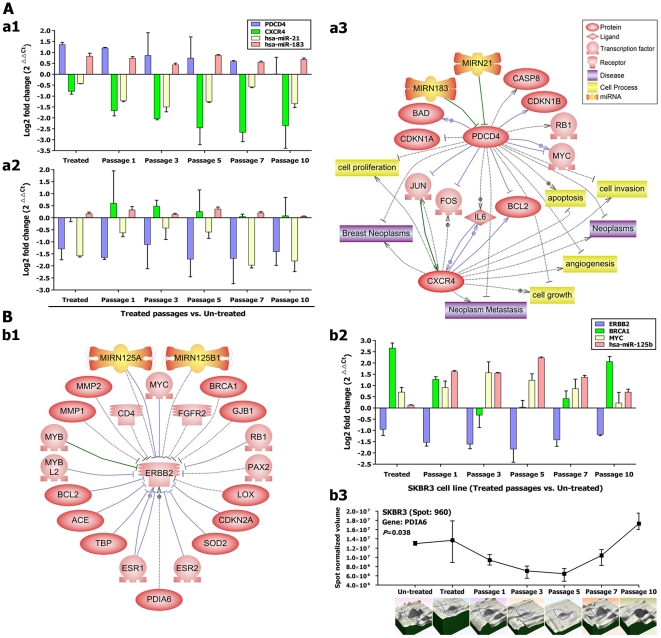
Dysregulation of important indicators of metastasis, prognosis and hormone receptors after treatment. A) Regulation of *PDCD4* and *CXCR4* genes as important indicators of metastasis and prognosis before and after treatment in two studied cancerous cell lines. a1-2) Expression profiles of *PDCD4* and *CXCR4* genes and regulatory microRNAs in MDA-MB231 and SKBR3 cell lines, respectively. a3) Pathway analysis predicts the role of *PDCD4* and *CXCR4* in cancerogenesis and shows the upstream regulatory miRNAs. B) Mechanism of *ERBB2* receptor down-regulation in SKBR3 (non-aggressive breast cancer cell line). b1) Pathway analysis shows negative upstream regulators of *ERBB2* gene and *PDIA6* as an example of positive regulators. b2) Expression profiles of *ERBB2* and some negative regulators (*BRCA1*, *MYC* and miR-125b) that demonstrated significant changes during treatment. b3) Expression profile of Pdia6 protein as a positive regulator of *ERBB2* that is detected by proteomics analysis.

In order to screen the direct effect of the DAC treatment on tumor metastasis, we analyzed the expression profile of *PDCD4*, *DHFR*, and *HOXD10*, three of the most promising genes implicated in the metastatic process [49], [51]. Among these metastasis related TSGs, only *PDCD4* was altered by the scheduled treatment and was significantly up-regulated in the highly aggressive cell line (MDA-MB231) and significantly down-regulated in the non-aggressive cell line (SKBR3) (Fig. 5A). Two miRNAs (miR-21 and miR-183), have complementary sites for the 3′-UTR of *PDCD4* and are considered to be possible suppressors of this gene [51], [52]. Interestingly, the co-expression analysis revealed down-regulation of miR-21 in the MDA-MB231, which was inversely correlated to *PDCD4* in the same cell line (*P*<0.001) (Fig. 5-a1). This finding is in line with previous reports suggesting miR-21 as a potential down-regulator element for *PDCD4* gene [51], [52]. However, miR-183 did not show any significant correlation with the expression of *PDCD4* in either of the two breast cancer cell lines (Fig. 5A), which is in contradiction with previous report [53]. This suggests that miR-21 (rather than the miR-183) is responsible for regulation of *PDCD4*.

Furthermore, *CXCR4* as one of 19 known human G protein–coupled chemokine receptors is specifically implicated in cancer metastasis and HIV-1 infection [54]. *CXCR4* has been associated with more than 23 cancer types promoting the metastasis, angiogenesis, tumor growth and poor prognosis of patients. Over-expression of *CXCR4* has also been shown in ∼10% of the tumors [55]. There is the possibility of using *CXCR4* as a therapeutic target against solid tumors [56]. Interestingly, *CXCR4* was significantly down-regulated in all scheduled follow-ups after demethylation treatment in MDA-MB231 (Fig. 5-a1). This finding suggests that down-regulation of *CXCR4* can be a secondary response to the demethylation of other upstream regulatory elements in the aggressive form of breast cancer.

Taken together, over-expression of *PDCD4*, inhibitor of cell proliferation and invasion through increasing the apoptosis, and conversely down-regulation of *CXCR4*, activator of cell proliferation and invasion, after treatment with DAC, suggests reduction of the invasion and metastasis for the highly aggressive subtype of breast cancer.

#### Suppression of ERBB2/HER2 receptor in the non-aggressive breast cancer subtype after DAC treatment

The estrogen receptor (ER), progesterone receptor (PR) and ERBB2/HER2 markers are used as prognosis indicators in breast cancer to stratify patients for appropriately targeted therapies [57]. After DAC treatment, there were no significant changes in hormonal receptor expression status (*ESR1*, *ESR2*, *PGR* and *ERBB2*) for HB2 (breast epithelial cell line) and MDA-MB231 (triple hormonal negative cell line). In SKBR3 (ERBB2/HER2 positive cell line), *ERBB2* showed a steady down-regulation after treatment (Fig. 5B). The *ERBB2* is a proto-oncogene that encodes a member of the epidermal growth factor (EGF) receptor family of tyrosine kinases. Amplification and/or over-expression of this gene have been reported in numerous cancers, including breast and ovarian tumors [58]. To understand the molecular mechanism of *ERBB2* down-regulation, we analyzed the expression profile of negative upstream regulators (Fig. 5-b1). From 21 candidates, two genes (*BRCA1* and *MYC*) and a miRNA (miR-125b) showed significant up-regulation in the treated cells. From these three negative regulators, expression of two (*MYC* and miR-125b) were inversely correlated with *ERBB2* expression (*P*<0.001) (Fig. 5-b2). MiR-125b, which was previously reported to be down-regulated in breast cancer [43], is involved in the regulation of *ERBB2* and *ERBB3*, impairing the downstream signaling pathway and the ability of the cells to grow and invade [59]. MiR-125b was significantly over-expressed as early and late effects only in SKBR3 (Table 2). Interestingly, in this regard we could identify a positive upstream regulator protein (Pdia6 protein) by mass spectrometry, which presented a significant correlation with *ERBB2* down-regulation (*P*<0.05) (Fig. 5-b3). Pdia6 is an activator for *ERBB2* which can contribute to the cell proliferation and breast neoplasm [60].

#### Dysregulation of drug tolerance genes/miRNAs

**Figure 6:**
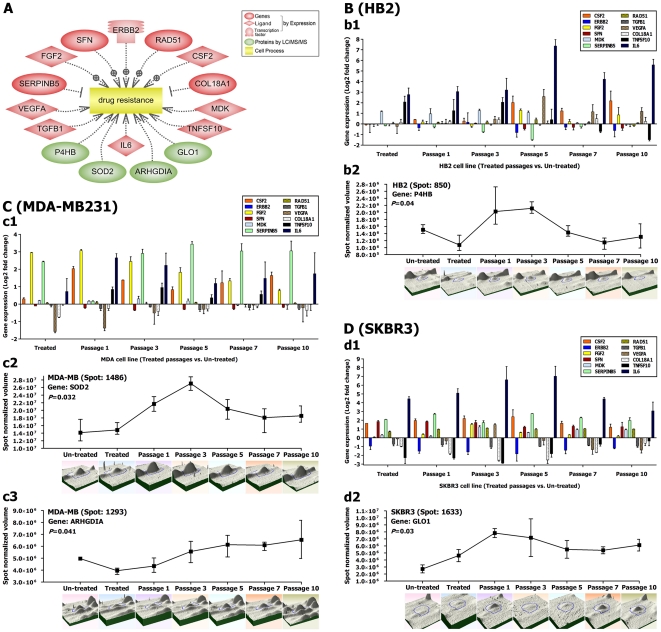
Drug tolerance after treatment with DAC. Candidate genes and proteins that are involved in drug tolerance with significant expression changes after treatment (A). Cell lines: HB2 (B), MDA-MB231 (C) and SKBR3 (D). Expression profiles of 12 candidate genes (*TGFB1*, *VEGFA*, *SERPINB5*, *FGF2*, *SFN*, *ERBB2*, *RAD51*, *CSF2*, *COL18A1*, *MDK*, *TNFSF10* and *IL6*) (b1, c1 and d1). Expression profiles of candidate proteins: P4hb (b2), Sod2 (c2), Arhgdia (c3) and Glo1 (d2).

The relatively rapid acquisition of resistance to cancer drugs remains a key obstacle for a successful cancer therapy [61]. Cancer-initiating cells have been proposed as potential culprits because of their capacity to escape from the drug effects by becoming quiescent [62]. To get insights into the underlying mechanisms of drug resistance against DAC, we checked the expression status of more than 40 candidate genes suggested by the pathway analysis that are involved in a variety of resistance mechanisms. From these candidate genes/proteins, 12 genes detected by differential transcriptomics (*IL6*, *TGFB1*, *VEGFA*, *SERPINB5*, *FGF2*, *SFN*, *ERBB2*, *RAD51*, *CSF2*, *COL18A1*, *MDK* and *TNFSF10*) and four proteins detected proteomically (P4hb, Sod2, Arhgdia and Glo1) were mostly up-regulated after DAC treatment (Table 2; Fig. 6). At the protein expression level, significant alterations over the scheduled follow ups were found for the P4hb protein in the HB2 cells, Sod2 and Arhgdia proteins in MDA-MB231 cells, and for the Glo1 protein in SKBR3 cells (Fig. 6).

The miRNAs expression alteration in response to the chemotherapy was previously studied and showed that Let-7i, miR-181a, -221, -27b, -34a, -424, -638 and -768 were up-regulated and miR-17, -21 and -28 were down-regulated after exposure to several drugs [63]. Our analysis of the genome-wide effects of DAC on cellular miRNAs, implicated significant down-regulation of miR-28 in all three cell lines, miR-21 in both cancerous cell lines and miR-17 in the non-aggressive cell line (Table 2; Dataset S5). At least three miRNA families were up-regulated: MiR-638 in all three cell lines, miR-34a in the highly aggressive line, miR-424 in the non-aggressive line, in a concordance with previous reports [63]. Other miRNAs were down-regulated upon treatment with DAC in all cell lines (miR-181a and -27b). In some instances, Let-7i and miR-221 presented different responses than reported in the literature [63]. In conclusion, most of the miRNAs with a role in drug resistance were down-regulated (Table 2) which suggests a lower resistance control of miRNAs after drug treatment.

#### Prominent regulatory role of miR-24 on the methylated *P16-INK4A* gene in the non-aggressive breast cancer subtype

**Figure 7:**
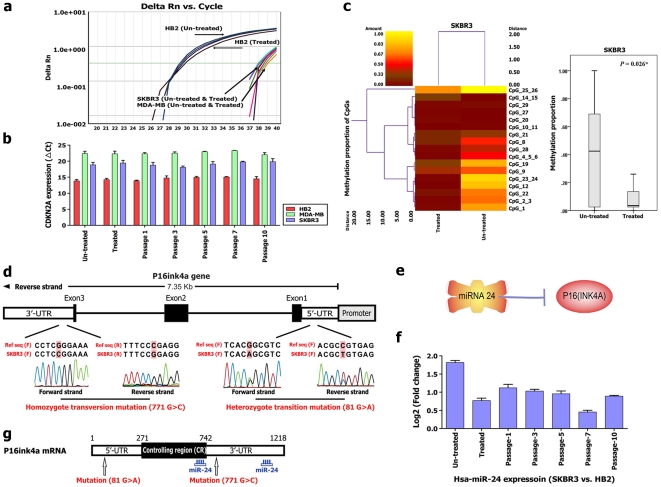
Epigenetic regulation of *P16-INK4A* tumor suppressor gene. a) Expression profile of *P16-INK4A* in treated and untreated cell lines; in two cancerous cell lines (MDA-MB231 and SKBR3) no expression was detected. b) Expression profile of *P16-INK4A* within all analyzed passages according to the normalized Ct values (ΔCt). c) Methylation proportion of informative CpG sites of *P16-INK4A* in SKBR3 cell line. d) Full gene sequencing of *P16-INK4A* revealed a heterozygote transition mutation in 5′UTR and a homozygote transversion mutation in 3′UTR of the gene. e) Pathway analysis predicts the role of has-miR-24 (MIRN24) as a down-regulator of the *P16-INK4A*. f) Expression profile of has-miR-24 in the non-aggressive breast cancerous cell line (SKBR3) versus control breast cell line (HB2). g) Comparison of miR-24 recognition site with the location of found mutations in the mature *P16-INK4A* mRNA.

*P16-INK4A* acts as an inhibitor of CDK4 kinase and in cooperation with *TP53* has a regulatory role for cell cycle G1 control. This gene is frequently silenced (through hypermethylation of specific CpG islands in the promoter, mutation, homozygote deletion or other epigenetic regulators) in many nonendocrine tumors [64], and this alteration may be predictive of the recurrence, tumor growth, or aggressiveness [36]. Our previous study showed significant promoter hypermethylation of *CDKN2A* (*P16-INK4A*) gene in breast cancer tissues as well as in the circulating cell free DNA (cff DNA) of patients [26], [65]. In the present study, *P16-INK4A* was not transcriptionally active and after treatment with DAC showed no significant change in the expression level in both cancer cell lines (Fig. 7a-b). It is reported that *P16-INK4A* has homozygote deletion (c.1_471 del 471) in MDA-MB231 cell line [66]. Our data from full gene sequencing also confirmed homozygote deletion in this cell line (data not shown).

SKBR3 cell line showed significant reduction in the methylation proportion of the *P16-INK4A* promoter (*P*<0.05) after treatment with DAC (Fig. 7c). In spite of *CDKN2A* hypomethylation induced by DAC, there wasn't any change in the expression level of the gene (Fig. 7b). In order to screen the possible mutation in the *P16-INK4A*, we performed full gene sequencing on the promoter region, 5′UTR, three exons including exon/intron boundaries (exon 1-3) and 3′UTR. The sequencing result showed two different mutations/polymorphisms, a heterozygote transition mutation in 5′UTR (c. 81 G>A) and a homozygote transversion mutation in 3′UTR (c.771 G>C) of the gene (Fig. 7d). These two mutations/polymorphisms were located in the UTR part of the gene that may not be the cause of lacking *P16-INK4A* expression in SKBR3.

Further investigation demonstrated steady up-regulation of miR-24 in SKBR3 after treatment and at all follow-up passages (Fig. 7f). MiR-24 has intriguing complementarities to 3′-UTRs and controlling region (CR) of the *P16-INK4A* and can suppress the gene (Fig. 7e) [67]. Two found mutations/polymorphisms are not located in the complementary site of the miR-24, and therefore they cannot be responsible for the change of the binding affinity of the miR-24 to the gene (Fig. 7g). The expression of miR-24 showed significant inverse correlation to *P16-INK4A* which might provide an explanation for *P16-INK4A* shut down in the non-aggressive cell line. These findings highlight the specific role of the epigenetic regulation of TSGs during carcinogenesis.

#### Inverse correlation of up-regulated miR-29b as a methylation suppressor, with expression *of DNMT3A* in the non-aggressive cell line

**Figure 8:**
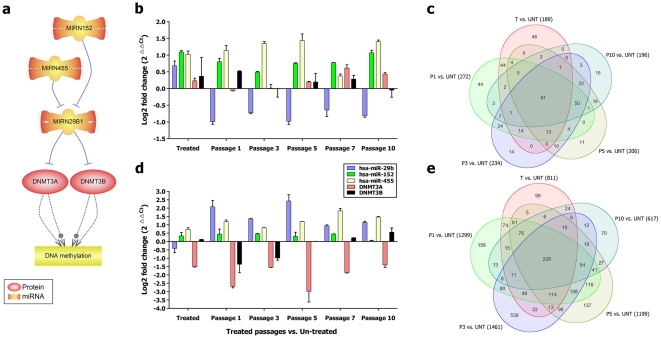
Role of has-miR-29b (MIRN29b) as a methylation suppressor during chemical methylation treatment. a) Pathway analysis predicted the inhibitory effects of MIRN29b for *DNMT3A* and *DNMT3B* genes and showed negative upstream regulators of MIRN29b. b) Expression profiles of MIRN29b, *DNMT3A*, *DNMT3B* and regulatory microRNAs in MDA-MB231 cell line. c) The number of significant up/down-regulated genes within different passages of MDA-MB231. d) Expression profiles of MIRN29b, *DNMT3A*, *DNMT3B* and regulatory microRNAs in SKBR3. e) The number of significant up/down-regulated genes within different passages of SKBR3.

The miRNA (miR)-29 family (29a, 29b, and 29c) has intriguing complementarities to 3′-UTRs of DNA methyltransferase (*DNMT*)*3A* and *-3B* (de novo methyltransferases), two key enzymes involved in DNA methylation, that are frequently up-regulated in solid tumors and associated with the poor prognosis. In lung squamous cell carcinomas, elevated Dnmt1 expression has been reported as a poor prognosis factor. Moreover, elevated expressions of both Dnmt1 and Dnmt3B have been shown to be correlated with hypermethylation of TSG promoters [68]. It was shown that expression of miR-29s, especially miR-29b, is directly targeting both *DNMT3A* and *-3B* and is inversely correlated to their expression in lung cancer tissues [69], [70]. In the present study, after demethylation treatment with DAC, the expression of miR-29b was inversely correlated with *DNMT3A* expression in SKBR3 (Fig. 8). Additionally, in SKBR3 cell line with up-regulation of miR-29b and steady down-regulation of *DNMT3A,* an almost 10 fold increase of number of the differentially expressed genes after demethylation treatment was found compared to MDA-MB231 (Fig. 8e vs. Fig. 8c). The presented data highlight a prominent role of Dnmt3A in the hypermethylation of promoters in the studied breast cancer cell lines. This finding also suggests that a combination of chemical demethylation treatment with *DNMT* inhibitors (decitabine or azacitidine) with enforced expression of miR-29b in breast cancer might have a synergistic hypomethylation effects that may result in a better disease response in breast cancer along with more robust gene re-expressions especially for the epigenetically silenced TSGs.

To understand the molecular mechanism of miR-29b deregulation, we analyzed the co-expression profiles of two important upstream suppressors (miR-152 and miR-455) [71]. Both of miR-29b suppressors, miR-152 and miR-455, were over-expressed in MDA-MB231 cell line. In SKBR3, along with the over-expression of miR-29b, miR-455 was also up-regulated whereas miR-152 did not show any significant change. These data suggest a prominent inhibitory effect of miR-152 rather than miR-455 on the regulation of miR-29b emphasizing the controlling effect of miRNAs in certain types of cancer.

### Conclusions

The aberrant DNA methylations of genes/miRNAs which are involved in malignant phenotypes provide interesting targets for chemotherapeutic intervention. To the best of our knowledge, the study presented here is the first pan-omics approach to synoptically identify epigenetic, transcriptome and proteome-wide alterations after effective demethylation treatment with DAC in the context of breast cancer.

The results of our synoptic pan-omics analysis provide a comprehensive view of DAC treatment defined by stable or transient early and late systematic effects in the model of cancer specific changes. We investigated DAC targets in depth (e.g. genes, miRNAs and proteins) and identified new pathways as well as biological interactions at the molecular levels that may correlate with particular steps in breast neoplasm including cell proliferation, cell/tissue invasion, oncogenesis, angiogenesis, apoptosis, neoplasm metastasis and senescence.

In total, SKBR3 (non-aggressive breast cancer cell line) showed more significant changes in the gene expression and proteomics levels than MDA-MB231 (highly aggressive breast cancer cell line) does. This finding highlights that the epigenetic treatment of hypermethylation genes during carcinogenesis, might have a more advantageous effect when cells are still less aggressive.

Additionally, along with the activation of several epigenetically suppressed TSGs, we also showed significant down-regulation of some important miRNAs with oncogenic functions in breast cancer cell lines (e.g. miR-21) as well as over-expression of some miRNAs with tumor suppressor functions (e.g. miR-155) that highlights the potential of a miRNA-based therapy in breast cancer. In the present study, there is a limitation of lacking inclusion of a luminal cell line to be compared with two studied basal like breast cancer subtypes MDA-MB231 (the triple negative receptor subtype) and SKBR3 (the Her2-associated subtype).

The presented approach might become a useful model for other human solid tumor malignancies, alone or in combination with other treatments such as enforced targeted therapies for miR-29b and/or IL6 (IL6 or IL6 receptor antagonists).

## Supporting Information

Dataset S1 **Complete materials and method:**
Eligibility criteria and study designCell lines and culture conditionsDAC treatmentsMultiplex quantification of cell viability and cytotoxicity protease activitiesQuantification of caspase-3 and caspase-7activitiesSimultaneous isolation of DNA, RNA miRNA and proteinsMethylation quantification of candidate tumor suppressor genes (TSGs) using thymidine-specific cleavage mass array on MALDI-TOF silico-chipMicroarray analysis and qRT-PCR validationsMutation screening of the *P16-INK4A* geneProteomic profilingLiquid Chromatography - Mass Spectrometry and Liquid Chromatography - Tandem Mass Spectrometry (LC-MS-MS)Gene ontology enrichment
*In silico* prediction of miRNA targetsCell signaling and pathway analysis
(PDF)

Dataset S2 **MRNA Expression Profiles.**
PCA analysis of studied cell lines (HB2, MDA-MB231 and SKBR3)K-means cluster analysis of three cell lines based on the Euclidean distance and variance within different treatment passagesThe number of significant up/down-regulated genes within different passagesHeatmaps of significant up/down-regulated oncogenesPathway analysis of oncogenes that are linked to breast neoplasms and metastasisHeatmaps of significant up/down-regulated tumor suppressor genesPathway analysis of tumor suppressor genes that are linked to breast neoplasms and metastasisExpression level of six cancer related genes after treatment using qPCR
(PDF)

Dataset S3 **MicroRNA Expression Profiles.**
PCA analysis of studied cell lines (HB2, MDA-MB231 and SKBR3)K-means cluster analysis of three cell lines based on the Euclidean distance and variance within different treatment passagesThe number of significant up/down-regulated miRNAs within different passagesPathway analysis of miRNAs that are linked to neoplasms, metastasis or carcinogenesisHeatmaps of significant up/down-regulated union miRNAsExpression level of important cancer related miRNAs using qPCR
(PDF)

Dataset S4 **Proteomic Profiles.**
Spot picking gels for significant up/down-regulated intersection proteinsExpression profile of candidate spots within different treatment passagesPathway analysis of proteins/isoforms in that are linked to neoplasms, metastasis or carcinogenesisExpression profiles of five genes/proteins that were detected similarly in both transcriptomic and proteomic analysis
(PDF)

Dataset S5 **List of significantly up/down-regulated genes/miRNAs as early or late effects of the treatment in three cell lines.**
(XLS)
