# Supplementary material for: Integrated Epigenetics of Human Breast Cancer: Synoptic Investigation of Targeted Genes, MicroRNAs and Proteins upon Demethylation Treatment
Source: PLoS One. 2011 Nov 4;6(11):e27355. doi: 10.1371/journal.pone.0027355 (PMC3208622; doi:10.1371/journal.pone.0027355)
Supplement: Dataset S1 — Complete materials and method: Eligibility criteria and study designCell lines and culture conditionsDAC treatmentsMultiplex quantification of cell viability and cytotoxicity protease activitiesQuantification of caspase-3 and caspase-7activitiesSimultaneous isolation of DNA, RNA miRNA and proteinsMethylation quantification of candidate tumor suppressor genes (TSGs) using thymidine-specific cleavage mass array on MALDI-TOF silico-chipMicroarray analysis and qRT-PCR validationsMutation screening of the P16-INK4A geneProteomic profilingLiquid Chromatography - Mass Spectrometry and Liquid Chromatography - Tandem Mass Spectrometry (LC-MS-MS)Gene ontology enrichment In silico prediction of miRNA targetsCell signaling and pathway analysis (PDF) [file pone.0027355.s001.pdf]

Dataset S1
Complete Materials and Methods

Eligibility criteria and study design

An optimal DAC concentration for the effective treatment was screened based on the cell viability, toxicity, apoptosis and DNA methylation for seven breast cell lines (six breast cancers and a breast epithelial cell line as a control). Pan-omics analysis at multiple molecular levels including epigenetic, transcriptomic (mRNA and microRNA expression) and proteomic analysis was performed before treatment, after treatment and at five follow-ups up to 10 passages at “drug holiday” condition for the two selected breast cancer cell lines and the control cell line. The study design workflow is illustrated in figure 1.

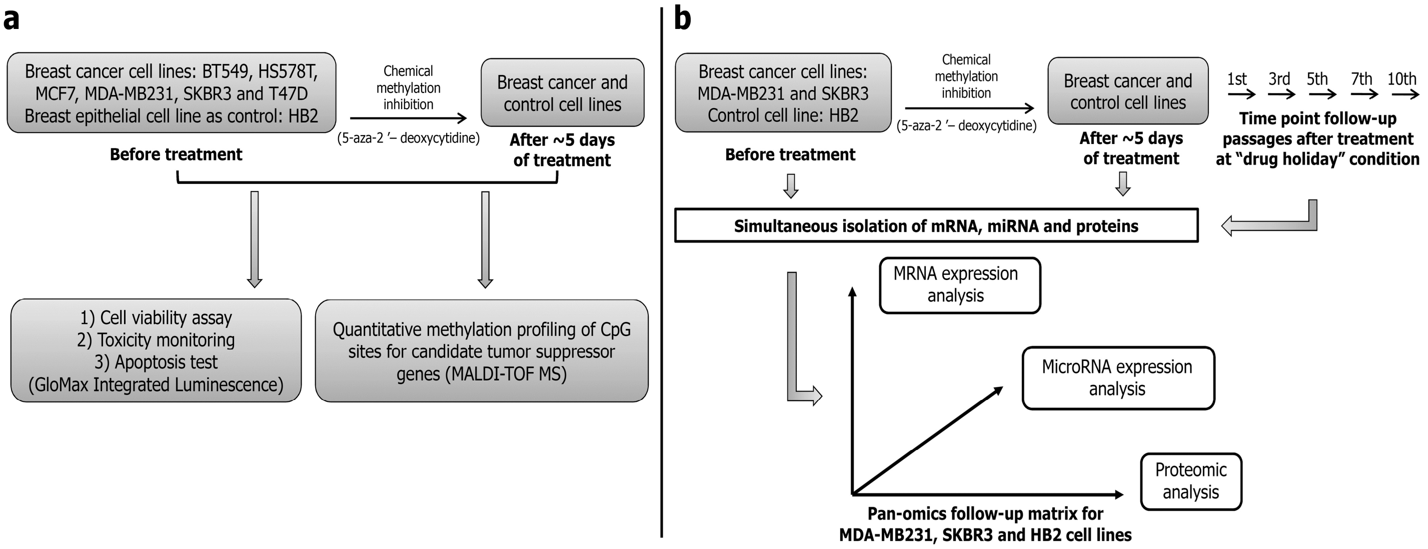

Fig. 1. The study design workflow. a) Screening for the optimal DAC treatment concentration based on the cell viability, toxicity, apoptosis and DNA methylation for six cancerous and a normal breast cell lines. b) Pan-omics follow-up (mRNA expression, microRNA expression and proteomics analysis) after treatment up to passage 10 for two cancerous and a normal breast cell lines.

Cell lines and culture conditions

A total of six breast cancer cell lines (MDA-MB-231, MCF-7, HS578T, BT549, T47D and SKBR3) and a breast epithelial cell line (HB2) were subjected for this study. The characteristics and media conditions are summarized in the table 1.

Table 1. Source, clinical and pathological features, and culture conditions of used cell lines.

Cell line	Gene cluster	Receptor status			TP53	Source	Tumor Type	Culture media
		ER	PR	ERBB2/HER2				
HB2	Lu	+	+	-	++WT	P.Br	-	DMEM, I, H
BT549	BaB	-	-	-	++M	P.Br	IDC, pap	RPMI
HS578T	BaB	-	-	-	+M	P.Br	IDC	DMEM
MCF7	Lu	+	+	-	+/-WT	PE	IDC	DMEM
MDA-MB231	BaB	-	-	-	++M	PE	AC	DMEM
SKBR3	Lu	-	-	+	+	PE	AC	McCoys 5A
T47D	Lu	+	+	-	++M	PE	IDC	RPMI

AC, adenocarcinoma; BaB, Basal B; IDC, invasive ductal carcinoma; Lu, luminal; Pap, papillary; P.Br, primary breast; PE, pleural effusion. ER/PR/HER2/TP53 status: ER/PR positivity, HER2 overexpression, and TP53 protein levels and mutational status (obtained from the Sanger web site; M, mutant protein; WT, wild-type protein) are indicated.

Media conditions: FBS, fetal bovine serum (10%); I, Insulin (0.01 mg/ml); H, hydrocortisone (500 ng/ml); cultured at $37^{o}C$, 5% $CO_{2;}$ DMEM, Dulbecco’s modified Eagle’s medium, GIBCO #11965-092; RPMI, RPMI medium 1640, GIBCO #27016-021.

DAC treatments

Cells were seeded (2 X l0^5 cells / T75 flasks) for 24h then media were removed and cells were freshly exposed to the DAC (Sigma Chemical Co., Switzerland) at concentrations of l0^1, l0^2, l0^3 or 10^4 nM in suspension culture every 24h until cultured cells reached 80-85 % confluence (~5 days). The cells were splitted for 10 follow-up passages at “drug holiday” condition.

Multiplex quantification of cell viability and cytotoxicity protease activities

Multiplex quantification of the cell viability and the cell cytotoxicity protease activities were assessed using the MultiTox-Glo Multiplex Cytotoxicity® Assay kit (Promega AG, Dübendorf, Switzerland) according to the manufacturer´s instructions. In this assay, two protease activities were measured (one as a marker of cell viability, and the other as a marker of cytotoxicity). The live-cell protease activity which is restricted to intact viable cells was measured using a fluorogenic, cell-permeant, peptide substrate (glycyl-phenylalanylaminofluorocoumarin, GF-AFC). This substrate enters intact cells, where it is cleaved by the live-cell protease activity to release AFC and generate a fluorescent signal that is proportional to the number of living cells. For this assay, 50 µL of the GF-AFC reagent was added to each well of a white-walled plate including ~5000 cells followed by mixing and incubation for one hour at 37°C. The live-cell fluorescence was measured at ~400nm$_{Ex}$/~505nm$_{Em}$ on a GloMax®-Multi Microplate Multimode Reader (Promega AG, Dübendorf, Switzerland).

A second, luminogenic cell-impermeant peptide substrate (alanyl-alanyl-phenylalanyl-aminoluciferin; AAF-Glo™ Substrate) was used to measure dead-cell protease activity, which is released from cells that have lost membrane integrity. The AAF-Glo™ Substrate is not cell-permeant; therefore, essentially no signal is generated from this substrate by intact, viable cells. Briefly, 50 µL of AAF-Glo™ reagent was added to each well from viability assay followed by orbital shaking, and incubated for 15 minutes at room temperature. The dead-cell fluorescence was measured at ~400nmEx/~505nmEm. Each sample was measured in triplex format and data were collected as relative fluorescence units (RFU).

Quantification of caspase-3 and caspase-7 activities

The caspases-3 and -7 activities were measured using the Caspase-Glo®3/7 Assay kit (Promega AG, Dübendorf, Switzerland) according to the manufacturer´s instructions. This kit is based on the cleavage of the amino acid sequence DEVD of a luminogenic substrate by the caspases-3 and -7 which results in a luminescent signal. For this assay 50 µL of freshly prepared Caspase-Glo Reagent was added to each well of a white-walled plate including ~5000 cells followed by two hours incubation at room temperature. Luminescence was measured at ~485nm$_{Ex}$/~527nm$_{Em}$ on a GloMax®-Multi Microplate Multimode Reader (Promega AG, Dübendorf, Switzerland). Each sample was measured in triplex format and data were collected as relative fluorescence units (RFU).

Simultaneous isolation of DNA, RNA miRNA and proteins

Three selected cell lines (MDA-MB231, SKBR3 and HB2) were subject for simultaneous isolation of DNA, RNA, miRNA and proteins (5×10^6 cells per sample) before treatment, after treatment and at five point follow-ups (1st, 3rd, 5th, 7th and 10th passages) using AllPrep® DNA/RNA/Protein Mini Kit (QIAGEN AG, Basel, Switzerland) according to the published protocol [1]. The RNeasy MinElute Cleanup Kit (QIAGEN AG, Basel, Switzerland) was used for isolation and purification of enriched miRNAs. The quantity of extracted molecular species was assessed using a NanoDrop ND-1000 spectrophotometer (Biolab, Mulgrave, VIC, Australia). Extracted RNA samples were analyzed for the size fractionation using RNA 6000 Nano LabChip® as well as using RNA 6000 Pico LabChip® for the enriched miRNAs (Agilent Technologies, GmbH, Waldbronn, Germany).

Methylation quantification of candidate tumor suppressor genes (TSGs) using thymidine-specific cleavage mass array on MALDI-TOF silico-chip

The EpiTYPER™ assay using matrix-assisted laser desorption/ionization time-of-flight mass spectrometry (MALDI-TOF MS) was assessed for the quantification of DNA methylation patterns of seven TSGs (*BMP6*, *BRCA1*, *CST6*, *CDKN1A*, *CDKN2A*, *P16-INK4A* and *TIMP3*) according to the previously published methods [2,3,4,5].

Bisulfite treatment. To perform bisulfite conversion of the target sequence, the Epitect® Bisulfite Kit (QIAGEN AG, Basel, Switzerland) was used.

Primer designing and PCR-tagging for EpiTYPER™ assay. CpG sites and density of target sequences in candidate TSGs were analyzed for the PCR primer design. We used previously designed and tagged primers (reverse primer with T7-promoter tag and forward primer with 10mer tag sequence as balance) for the candidate genes. The primer sequences are summarized in table 2. Selected amplicons were mostly located in the promoter regions, or started from the promoter and partially covered the first exon [3]. For the PCR on bisulfite-treated genomic DNA (gDNA), the following PCR condition was used: 1x: 95°C for 10 min; 48x: 95°C for 30s, Ta for 40s, 72°C for 1 min; 1x 72°C for 5 min. The PCR cocktail was: 2µL DNA (2.00µL of at least 10 ng/µL DNA for a final concentration of 2ng/µL per reaction) in a 10µL total volume using 10 pmol of each primer, 200µM dNTP, 0.2 unit Hot Start Taq DNA polymerase, 1.5mM $MgCl_2$ and the buffer supplied with the enzyme.

***In vitro* transcription, T-cleavage assay and mass spectrometry.** *In vitro* transcription and T-cleavage were assessed according to the previously published methods [3,5,6]. Twenty-two nanoliters of cleavage reaction were robotically dispensed (nanodispenser) onto silicon chips preloaded with matrix (SpectroCHIP; SEQUENOM, San Diego). Mass spectra were collected using a MassARRAY Compact MALDI-TOF (SEQUENOM) and spectra's methylation ratios were generated by the Epityper software v1.0 (SEQUENOM, San Diego).

Table 2. The sequence of PCR tagged primers for *in vitro* transcription.

Gene Name	Primer	Sequence (5'→3')	Length	T_a	Prodoct Size (bp)
BMP6	tag-EN1-FW	AGGAAGAGAGGGGGTAAATTTTATGGTGGTTT	22+10	57	397
	T7-EN1-RV	CAG**TAATACGACTCACTATAGGGAGA**AGGCTCCTTCCTAACCCTCAATCCTTA	22+31		
BRCA1	tag-EN1-FW	AGGAAGAGAGAATTGGAGATTTTTATTAGG	20+10	56	413
	T7-EN1-RV	CAG**TAATACGACTCACTATAGGGAGA**AGGCTAAATCTCAACRAACTCAC	18+31		
CST6	tag-EN1-FW	AGGAAGAGAGGTTGGTAGTTTATTTTGGATAGTTT	25+10	59	445
	T7-EN1-RV	CAG**TAATACGACTCACTATAGGGAGA**AGGCTCAAATCCCRAAATTCTCC	18+31		
CDKN1A	tag-EN1-FW	AGGAAGAGAGGGTAAATTTTTGTTTGTTAGAGTGG	25+10	60	419
	T7-EN1-RV	CAG**TAATACGACTCACTATAGGGAGA**AGGCTTAACTTCRACAACTACTCACACCT	24+31		
CDKN2A	tag-EN1-FW	AGGAAGAGAGGGTTGTTTTTGGTAGGG	17+10	58	580
	T7-EN1-RV	CAG**TAATACGACTCACTATAGGGAGA**AGGCTATATAAACCACRAAAACCC	19+31		
P16-INK4A	tag-EN1-FW	AGGAAGAGAGTGGGGTTTTTATAATTAGGAAAGAATA	27+10	57	533
	T7-EN1-RV	CAG**TAATACGACTCACTATAGGGAGA**AGGCTCCACCCTCTAATAACCAACCAA	22+31		
TIMP3	tag-EN1-FW	AGGAAGAGAGTTTTGTTATTGGTTTGAGGG	20+10	59	441
	T7-EN1-RV	CAG**TAATACGACTCACTATAGGGAGA**AGGCTCCAAACTCCAACTACCCA	18+31		

Microarray analysis and qRT-PCR validations

MRNA expression analysis. Microarray analyses of mRNA samples at various follow-up passages including two independent biological replicates were conducted using the Affymetrix Genome 133 Plus 2.0 GeneChips (Affymetrix Inc., Santa Clara, U.S.). The synthesis of cDNA was carried out with a starting amount of approximately 50 ng total RNA using the NuGEN Ovation RNA Amplification System V2 (NuGEN Technologies Inc.; San Carlos, U.S.). The arrays were hybridized with the biotin-labeled fragments and rotated in the hybridization oven for 18 hours at 45°C and 60 rpm. The arrays were washed and stained with a streptavidin phycoerythrin conjugate on GeneChip Fluidics 450 Workstations, and scanned on a GeneChip Scanner 3000 7G (Affymetrix Inc.) The expression data were acquired using the Affymetrix GeneChip Operating Software (GCOS). The system was used to generate the numerical values of the probe intensity (Signals). HGU133Plus2 annotations (v2.1.0, ENTREZG) supplied by University of Michigan, was used as a probe identifier (http://brainarray.mbni.med.umich.edu/Brainarray/Database/CustomCDF/12.1.0/entrezg.asp).

MicroRNA expression analysis. Microarray analyses of enriched miRNA samples at various follow-up passages were carried out using Affymetrix GeneChipR miRNA array (v1.0). The labeling of the microRNAs was carried out with a starting amount of approximately 100 ng small RNA using the FlashTag Biotin HSR RNA Labeling Kit (Genisphere). The labeling process was performed in two stages (i) Poly (A) Tailing (ii) FlashTag Biotin HSR Ligation. After hybridization with the biotin-labeled fragments, arrays were rotated 16 hours at 48°C and 60 rpm. The arrays were washed and stained with a streptavidin phycoerythrin conjugate on GeneChip Fluidics 450 Workstations, and scanned on a GeneChip Scanner 3000 7G (Affymetrix Inc.) The expression data were acquired using the Affymetrix GeneChip Command Console Software (AGCC) and miRNA-1_0 annotations (20081203) supplied by Affymetrix was used as a probe identifier.

Data analysis. In order to avoid random fluctuations in gene expression, after log transformation and Robust Multi-array Analysis (RMA) normalization, differentially expressed mRNAs/miRNAs were defined by applying three stringent filtering criteria (mean intensity greater than six, fold change greater than two and ANOVA set to $P<0.05$) using Partek Genomics Suite software v6.5 (Partek Incorporated, Missouri, USA). MiRNAs that were differentially expressed between untreated and treated or follow-up passages were identified using a paired t–test at $P<0.05$ significance. Present study reported differentially expressed mature miRNAs. Unsupervised hierarchical clustering of significant up/down-regulated genes/miRNAs were applied using Partek software with standard Pearson’s correlation as similarity measurement, and Ward’d method for clustering the data.

Quantitative RT-PCR validation. To confirm the microarray findings, quantitative real-time (qRT) PCR was used for several candidate mRNAs and miRNAs (Table 3 and 4). For each cell line, RNA used for microarray analysis was also used to synthesize the cDNA using High Capacity cDNA Reverse Transcription Kit (Applied Biosystems, USA). Real-time primers were then designed for each gene using Primerquest Software (Integrated DNA Technologies). For qRT-PCR analysis, synthesized cDNAs amplified with specific gene primers using SYBR® Green 2X PCR Master Mix (Applied Biosystems). In order to analyze miRNAs with qRT-PCR the miScript PCR system kit was used with the commercially available primers for mature miRNAs (QIAGEN AG, Basel, Switzerland) (Table 4). Raw values were normalized using geometric mean of seven reference genes (*18S*, *GAPDH*, *GRB2*, *Hmox2*, *TMEM184B*, *USP7* and *ZNF398*) for mRNA expression and two reference miRNAs (miR-151 and miR-193a) for miRNA expression as internal controls. These reference genes/miRNAs were selected according to their low variability overall microarray data in all the analyzed samples [7,8,9,10]. Real-time PCR reactions were performed in two replicates and including no-template controls using ABI Prism 7000 Sequence Detection System (Applied Biosystems). The fold difference for each sample was calculated using the comparative Ct method [11].

Table 3. The primer sequence of selected mRNAs for qRT-PCR.

Gene Name	Ensembl Gene ID	Primer	Sequence (5’→3’)	Length (bp)	T_a	Prodoct Size (bp)
18S*	ENST00000445125	FW	CGCCGCTAGAGGTGAAATTCT	21	60.1	66
		RV	CATTCTTGGCAAATGCTTTCG	21		
BRCA1	ENST00000357654	FW	AGTTGGTCTGAGTGACAAGG	20	60.7	109
		RV	CTGCTTCACCTAAGTTTGAATCC	23		
CDKN1A (P21)	ENST00000244741	FW	TGCGTTCACAGGTGTTTCTG	20	60.3	217
		RV	GTCACCCTCCAGTGGTGTCT	20		
P16-INK4A	ENST00000304494	FW	GAGGAAGAAAGAGGAGGG	17	59.5	265
		RV	CATCATGACCTGGATCGG	18		
CST6	ENST00000312134	FW	CTACTTCCGAGACACGCACA	20	59.8	201
		RV	GGAACCACAAGGACCTCAAA	20		
CXCR4	ENST00000241393	FW	CCAGAAACTTCAGTTTGTTGG	21	60.79	242
		RV	ATGATGGAGTAGATGGTGGG	20		
DNMT1	ENST00000340748	FW	CTGGCTTTGAGAGTTATGAGG	21	60.4	182
		RV	CATTAACACCACCTTCAAGAG	21		
DNMT3A	ENST00000264709	FW	GATGATTGATGCCAAAGAAGTG	22	60.2	272
		RV	CCAAATACCCTTTCCATTTCAG	22		
DNMT3B	ENST00000328111	FW	CAAGGAAATACGAGAACAAGAC	22	59.3	195
		RV	GACAAACAGCCATCTTCCAG	20		
ERBB2	ENST00000269571	FW	GCCTGTCCCTACAACTACC	19	61	178
		RV	GTAACTGCCCTCACCTCTC	19		
ESR1	ENST00000206249	FW	CAGACACTTTGATCCACCTG	20	60.63	115
		RV	GCCTTTGTTACTCATGTGCC	20		
ESR2	ENST00000341099	FW	GGATGGAGGTGTTAATGATGG	21	60.53	233
		RV	GAGGGTACATACTGGAATTGAG	22		
ETS1	ENST00000319397	FW	TCACTAAAGAACAGCAACGAC	21	60.2	250
		RV	TGGTTTCACATCCTCTTTCTG	21		
GAPDH*	ENST00000229239	FW	GAAGGTGAAGGTCGGAGT	18	60	226
		RV	GAAGATGGTGATGGGATTTC	20		
GJA1 (CX43)	ENST00000282561	FW	AGGAAAGTACCAAACAGCAG	20	60.2	209
		RV	CAGTTGAGTAGGCTTGAACC	20		
GRB2*	ENST00000316804	FW	AGAAATGAAACCACATCCGT	20	60.3	155
		RV	ACATCGTTTCCAAACTTGACAG	22		
Hmox2*	ENST00000219700	FW	AACCAAATGAGAATGGCTGAC	21	61.2	164
		RV	GGCTGAGTATGTGAAGTAAAGTG	23		
IL6	ENST00000404625	FW	CACTCACCTCTTCAGAACGA	20	61.08	196
		RV	GCAAGTCTCCTCATTGAATCC	21		
MERTK	ENST00000295408	FW	TCAACATCAAAGCAATTCCCTC	22	61.05	205
		RV	GATTTGGTACAGATGTGGTAAGG	23		
MYC	ENST00000377970	FW	GATTCTCTGCTCTCCTCGAC	20	61.17	110
		RV	TTCTTGTTCCTCCTCAGAGTC	21		
PDCD4	ENST00000280154	FW	GAAGTTGCGGAAATGTTAAGAG	22	60.17	259
		RV	ACAGCTCTAGCAATAAACTGG	21		
PGR	ENST00000263463	FW	CAATGGAAGGGCAGCAC	17	60.58	231
		RV	CCACTGACGTGTTTGTAGG	19		
PTEN	ENST00000371953	FW	CGGAACTTGCAATCCTCAG	19	60.8	233
		RV	GTTTCCTCTGGTCCTGGT	19		
RASSF1	ENST00000359365	FW	AAGTTCACCTGCCACTACC	19	60.4	217
		RV	CCGTCCTTGTTCAAGCTC	18		
TMEM184B*	ENST00000361906	FW	CACCAGATCTACATGCACCT	20	61.3	`277
		RV	TACATACAGCTGGACTCAATGG	22		
USP7*	ENST00000344836	FW	ATTCCTAACATTGCCACCAG	20	60.1	256
		RV	ATTTACACCATTTGCCATCC	20		
VEGFA	ENST00000372067	FW	GAGTACCCTGATGAGATCGAG	21	60.05	213
		RV	CTTTCTTTGGTCTGCATTCAC	21		
ZNF398*	ENST00000420008	FW	CAGGTATTAAGGGAGATATCCCA	23	60.2	161
		RV	TAGCATAATCCATGGAGATGAG	22		

* selected as a reference gene

Table 4. The primer sequence and ID of validated miRNAs by qRT-PCR.

MIR ID	Sanger ID	Sanger Accession	Sequence 5‘ → 3‘
hsa-mir-125b-1	hsa-miR-125b	MIMAT0000423	UCCCUGAGACCCUAACUUGUGA
hsa-mir-130a	hsa-miR-130a	MIMAT0000425	CAGUGCAAUGUUAAAAGGGCAU
hsa-mir-151**	hsa-miR-151-3p	MIMAT0000757	CUAGACUGAAGCUCCUUGAGG
hsa-mir-152	hsa-mir-152	MIMAT0000438	UCAGUGCAUGACAGAACUUGG
hsa-mir-181a	hsa-miR-181a	MIMAT0000256	AACAUUCAACGCUGUCGGUGAGU
hsa-mir-183	hsa-mir-183	MIMAT0000261	UAUGGCACUGGUAGAAUUCACU
hsa-mir-193a**	hsa-miR-193a-5p	MIMAT0004614	UGGGUCUUUGCGGGCGAGAUGA
hsa-miR-21_2	hsa-miR-21	MIMAT0000076	UAGCUUAUCAGACUGAUGUUGA
hsa-miR-24_1	hsa-miR-24	MIMAT0000080	UGGCUCAGUUCAGCAGGAACAG
hsa-mir-27a	hsa-miR-27a	MIMAT0000084	UUCACAGUGGCUAAGUUCCGC
hsa-mir-27b	hsa-miR-27b	MIMAT0000419	UUCACAGUGGCUAAGUUCUGC
hsa-miR-29b_1	hsa-miR-29b	MIMAT0000100	UAGCACCAUUUGAAAUCAGUGUU
hsa-mir-378	hsa-miR-378	MIMAT0000732	ACUGGACUUGGAGUCAGAAGG
hsa-mir-455	hsa-miR-455-3p	MIMAT0004784	GCAGUCCAUGGGCAUAUACAC
hsa-miR-99a_1	hsa-miR-99a	MIMAT0000097	AACCCGUAGAUCCGAUCUUGUG

** selected as a reference miRNA

Mutation screening of the P16-INK4A gene

Full gene sequencing of *P16-INK4A* was performed on the promoter region, 5’UTR, three exons including exon/intron boundaries (exon 1-3) and 3’UTR. Information of primer sequences are listed in Table 5. The PCR for each region carried out in a 50 µL total volume containing 100ng DNA, 200µM of each dNTP, 20 pmoL of each primer, 2.5 U Hot Start Taq DNA polymerase, 1X PCR buffer and 1.5 mM MgCl2**.** The PCR was performed in 40 cycles under following condition: denaturation at 94°C for 15 sec, annealing step for 15 sec at appropriate T_a that is mentioned in Table 5, and 60 sec primer extension at 72°C. Direct DNA sequencing was performed using a Big Dye terminator v3.1 cycle sequencing kit and automated sequencer was performed (ABI 3130, Applied Biosystem). The results of DNA sequence analysis were compared with the reference sequences of the gene bank (www.ncbi.nlm.mih.gov/nucleotide) using DNASTAR sequence alignment software (DNASTAR Lasergene 8, Inc., Madison, USA). All sequencing reactions were performed in both directions and confirmed for concordance.

Table 5. The primer sequence for the *P16-INK4A* gene sequencing.

Gene Name	Primer	Sequence (5’→3’)	Length (bp)	T_a	Prodoct Size (bp)
***P16-INK4A* (Promoter)**	FW	GCTCCTGAAAATCAAGGGTTG	21	58	648
	RV	CCTGCTCTCCCCCTCTCC	18		
P16-INK4A* (5’UTR + Exon1*)	FW	AGTCCTCCTTCCTTGCCAAC	20	57	620
	RV	CTTCTGAAAACTCCCCAGGA	20		
***P16-INK4A* (Exon2)**	FW	AATTAGACACCTGGGGCTTG	20	57	582
	RV	AGGGCGATAGGGAGACTCAG	20		
***P16-INK4A* (Exon3 + 3’UTR)**	FW	GTAGGGACGGCAAGAGAGG	19	57	660
	RV	TGAAACAACAGTGTCAGAAACG	22		

Proteomic profiling

Protein pallets from AllPrep® extraction (5 x 10^6 cells per sample) were directly solubilized in a buffer consisting 7M urea, 2M thiourea, 4% CHAPS, 20mM DTT and 2% ampholines, pH 3-10 (Invitrogen) for a total volume of 0.1mL.

Two-dimensional gel electrophoresis (2DE) and spot visualization. The first dimension was run using the ISODALT system (ampholines pH 3-10 (Invitrogen)) [12,13]; and for the second dimension 11-19% linear acrylamide gradient was used. Protein spots were visualized by silver staining and wet silver-stained gels were scanned by Pharmacia Image Scanner with 300 dpi and 16-bit, based on the published method [1].

Image analysis and spot quantification. Each protein sample was run by 2DE in triplicate in order to evaluate gel reproducibility and improve the reliability of the qualitative and qualitative changes in the protein expression measurement by means of electrophoresis. Progenesis SameSpot software (v 4.0, NonLinear Dynamics, UK) was used for gel alignment, spot detection, spot quantification, and normalization for the total spot volume in each gel, and the data were statistically analyzed using the incorporated statistical package. Gel images were automatically aligned after manually assigning 20 landmark vectors. The samples variables were expressed as mean of each replicate (SE or percentages, and were statistically analyzed by ANOVA). The cutoff level for a differentially expressed protein was defined based on an ANOVA at significance of $P<0.05$ using the SameSpots software considering a minimum of 1.5-fold change (normalized volume). In addition, we applied separate non-parametric statistical analyses between each pair of samples including untreated vs. treated and other follow-up passages.

Liquid Chromatography - Mass Spectrometry and Liquid Chromatography - Tandem Mass Spectrometry (LC-MS-MS)

The protein spots of interest were manually excised from gels and underwent in-gel digestion with trypsin. The trypsin digested proteins were analyzed by capillary liquid chromatography tandem MS (LC-MS-MS) using a setup of a ProteoCol trap C-18 column (0.15 x 10mm, 3 μm particle size, 300Å) (SGE Analytical Science, Victoria, AU) and a separating column (0.1mm x10cm) that had been packed with Magic 300Å C18 reverse-phase material (5 mm particle size, Swiss Bioanalytics, Birsfelden, Switzerland). The columns were connected on line to an Orbitrap FT hybrid instrument (Thermo Finnigan, San Jose, CA, USA). The solvents used for peptide separation consisted 0.1% acetic acid in water (solvent A) and 0.1% acetic acid and 80% acetonitrile in water (solvent B). Peptides were injected via a 2 μL loop to the trap column with the capillary pump of an Agilent 1200 system set to 5 μL/min. After 15 min, the trap column was switched into the flow path of the separating column. A linear gradient from 2 to 35% solvent B in solvent A in 60 min was delivered with an Agilent 1200 nano pump at a flow rate of 500 nL/min. After 60 min the percentage of solvent B was increased to 60% in ten minutes and further increased to 80% in 2 min. The eluting peptides were ionized at 1.7 kV. The mass spectrometer was operated in a data-dependent fashion. The precursor scan was done in the Orbitrap set to 60,000 resolutions, while the fragment ions were mass analyzed in the LTQ instrument. A top five method was run so that the five most intense precursors were selected for fragmentation.

Peptide identification. The MS-MS spectra were then searched against the human data bank (NCBI non-redundant, version October 1st, 2010) using TurboSequest software [14]. The data bank was searched with 10 ppm precursor ion tolerance, while the fragment ions were set to 0.5 Da tolerances. Cleavage rules were set to fully enzymatic – cleaves at both ends, allowing 2 missed cleavages. Post filtering was set to the following parameters: ΔCN, 0.1; Xcorr versus charge state was 1.50 (1+), 2.00 (2+), 2.50 (3+); peptide probability, 0.01; protein probability 0.01. Valid identification required two or more peptides independently matching the same protein sequence, a significant peptide score ($P<0.05$), and the manual confirmation of agreement between the spectra and peptide sequence. At least two peptides were required for protein identification. Protein quantification and data validation was performed using Scaffold (version Scaffold 3.00.06; Proteome Software, Portland, OR, USA), which models the score distributions of the entire dataset of spectra. Database search files generated by TurboSequest were imported into Scaffold and analyzed using the tandem searches against the same protein sequence database the same search parameters as the associated TurboSequest search.

Gene ontology enrichment

The gene ontology (GO) enrichment was assessed using Partek Genomics Suite software v6.5 (Partek Incorporated, Missouri, USA), which could take a list of significantly expressed genes and see how they group in the functional hierarchy. The enrichment scores were calculated using a chi-square test comparing the proportion of the gene list in a group to the proportion of the background in the group. If a functional

group had an enrichment score over 1, the functional category was over expressed. A value of 3 or higher corresponds to significant over expression (*P*<0.05).

In silico prediction of miRNA targets

Conserved and non-conserved targets of detected miRNAs were identified using TargetScan 5.1 (http://www.targetscan.org/). The list of predicted targets was obtained from TargetScan data download and further investigated using pathway analysis. For important miRNAs, the mRNA targets were validated to find exact miRNA complementary sites using RNA22 program (http://cbcsrv.watson.ibm.com/rna22.html). The default stringency settings were used: maximum number of allowed UN-base paired bases =0 in seed/nucleus of 7 nucleotides, and minimum number of paired-up bases in heteroduplex =14; maximum folding energy for heteroduplex (Kcal/mol =225).

Cell signaling and pathway analysis

Gene networks and canonical pathways representing key genes, miRNAs and proteins were identified using the Pathway Studio® software version 7 and ResNet® 7 (Mammal) database (Ariadne Genomics, Inc., Rockville, USA). The data set containing gene/miRNA identifiers and corresponding fold changes were uploaded into the Pathway Studio and each gene identifier was mapped to its corresponding gene object. The functional analysis identified the biological functions and/or diseases that were most relevant to the data sets and facilitated the understanding beyond their functional link to breast neoplasm. This analysis provided an approach to compare different kinds of cellular interactions, including protein-protein interactions as well as genes-miRNAs interactions.

Reference

1. Radpour R, Sikora M, Grussenmeyer T, Kohler C, Barekati Z, et al. (2009) Simultaneous Isolation of DNA, RNA, and Proteins for Genetic, Epigenetic, Transcriptomic, and Proteomic Analysis. J Proteome Res 8: 5264-5274.
2. Radpour R, Barekati Z, Haghighi MM, Kohler C, Asadollahi R, et al. (2010) Correlation of telomere length shortening with promoter methylation profile of p16/Rb and p53/p21 pathways in breast cancer. Mod Pathol 23: 763-772.
3. Radpour R, Kohler C, Haghighi MM, Fan AX, Holzgreve W, et al. (2009) Methylation profiles of 22 candidate genes in breast cancer using high-throughput MALDI-TOF mass array. Oncogene 28: 2969-2978.
4. Ehrich M, Turner J, Gibbs P, Lipton L, Giovanneti M, et al. (2008) Cytosine methylation profiling of cancer cell lines. Proc Natl Acad Sci U S A 105: 4844-4849.
5. Radpour R, Haghighi MM, Fan AX, Torbati PM, Hahn S, et al. (2008) High-Throughput Hacking of the Methylation Patterns in Breast Cancer by In vitro Transcription and Thymidine-Specific Cleavage Mass Array on MALDI-TOF Silico-Chip. Mol Cancer Res 6: 1702-1709.
6. Ehrich M, Nelson MR, Stanssens P, Zabeau M, Liloglou T, et al. (2005) Quantitative high-throughput analysis of DNA methylation patterns by base-specific cleavage and mass spectrometry. Proc Natl Acad Sci U S A 102: 15785-15790.
7. Gur-Dedeoglu B, Konu O, Bozkurt B, Ergul G, Seckin S, et al. (2009) Identification of endogenous reference genes for qRT-PCR analysis in normal matched breast tumor tissues. Oncol Res 17: 353-365.
8. Kwon MJ, Oh E, Lee S, Roh MR, Kim SE, et al. (2009) Identification of novel reference genes using multiplatform expression data and their validation for quantitative gene expression analysis. PLoS One 4: e6162.
9. Peltier HJ, Latham GJ (2008) Normalization of microRNA expression levels in quantitative RT-PCR assays: identification of suitable reference RNA targets in normal and cancerous human solid tissues. RNA 14: 844-852.

10. Latham GJ (2010) Normalization of microRNA quantitative RT-PCR data in reduced scale experimental designs. Methods Mol Biol 667: 19-31.
11. Livak KJ, Schmittgen TD (2001) Analysis of relative gene expression data using real-time quantitative PCR and the 2(-Delta Delta C(T)) Method. Methods 25: 402-408.
12. Grussenmeyer T, Meili-Butz S, Dieterle T, Traunecker E, Carrel TP, et al. (2008) Quantitative Proteome Analysis in Cardiovascular Physiology and Pathology. I. Data Processing. J Proteome Res.
13. Anderson NL, Anderson NG (1978) Analytical techniques for cell fractions. XXII. Two-dimensional analysis of serum and tissue proteins: multiple gradient-slab gel electrophoresis. Anal Biochem 85: 341-354.
14. Gatlin CL, Eng JK, Cross ST, Detter JC, Yates JR, 3rd (2000) Automated identification of amino acid sequence variations in proteins by HPLC/microspray tandem mass spectrometry. Anal Chem 72: 757-763.
