# Supplementary material for: Integrated Epigenetics of Human Breast Cancer: Synoptic Investigation of Targeted Genes, MicroRNAs and Proteins upon Demethylation Treatment
Source: PLoS One. 2011 Nov 4;6(11):e27355. doi: 10.1371/journal.pone.0027355 (PMC3208622; doi:10.1371/journal.pone.0027355)
Supplement: Dataset S2 — MRNA Expression Profiles. PCA analysis of studied cell lines (HB2, MDA-MB231 and SKBR3)K-means cluster analysis of three cell lines based on the Euclidean distance and variance within different treatment passagesThe number of significant up/down-regulated genes within different passagesHeatmaps of significant up/down-regulated oncogenesPathway analysis of oncogenes that are linked to breast neoplasms and metastasisHeatmaps of significant up/down-regulated tumor suppressor genesPathway analysis of tumor suppressor genes that are linked to breast neoplasms and metastasisExpression level of six cancer related genes after treatment using qPCR (PDF) [file pone.0027355.s002.pdf]

Dataset S2

MRNA Expression Profiles

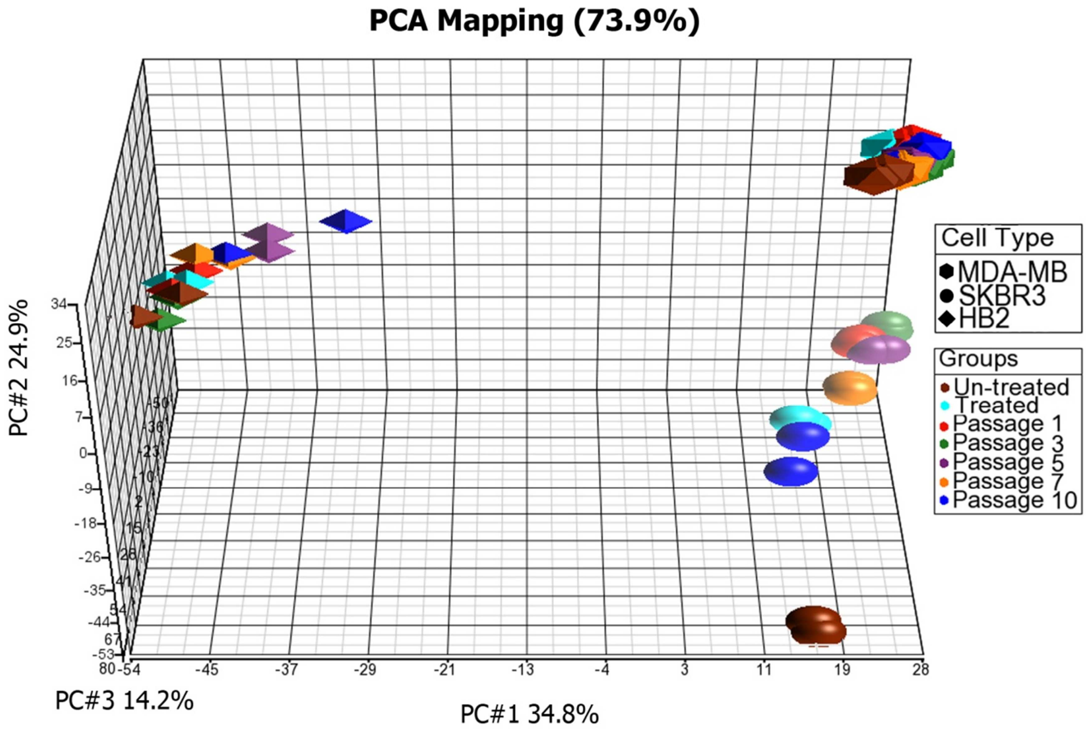

PCA analysis of three different studied cell lines (HB2, MDA-MB231 and SKBR3).

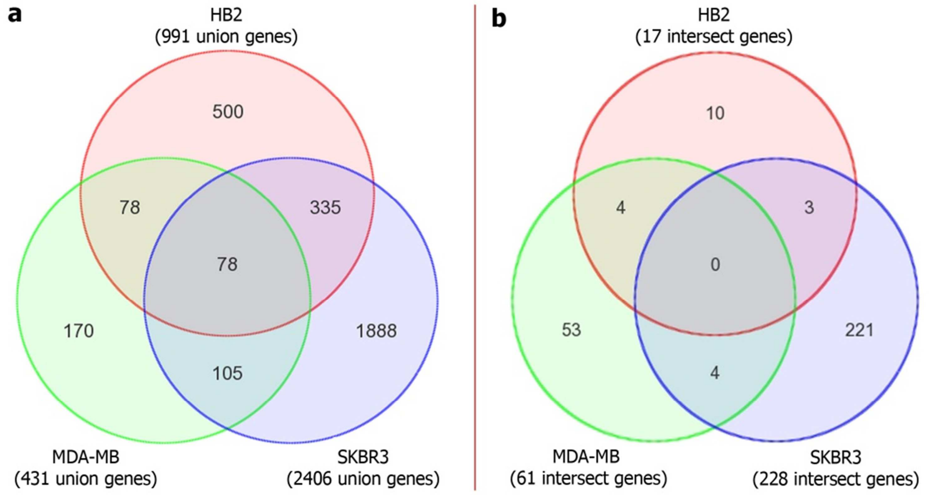

a) Comparison of significant up/down-regulated union genes in three analyzed cell lines.
b) Comparison of significant up/down-regulated intersection genes in three analyzed cell lines.

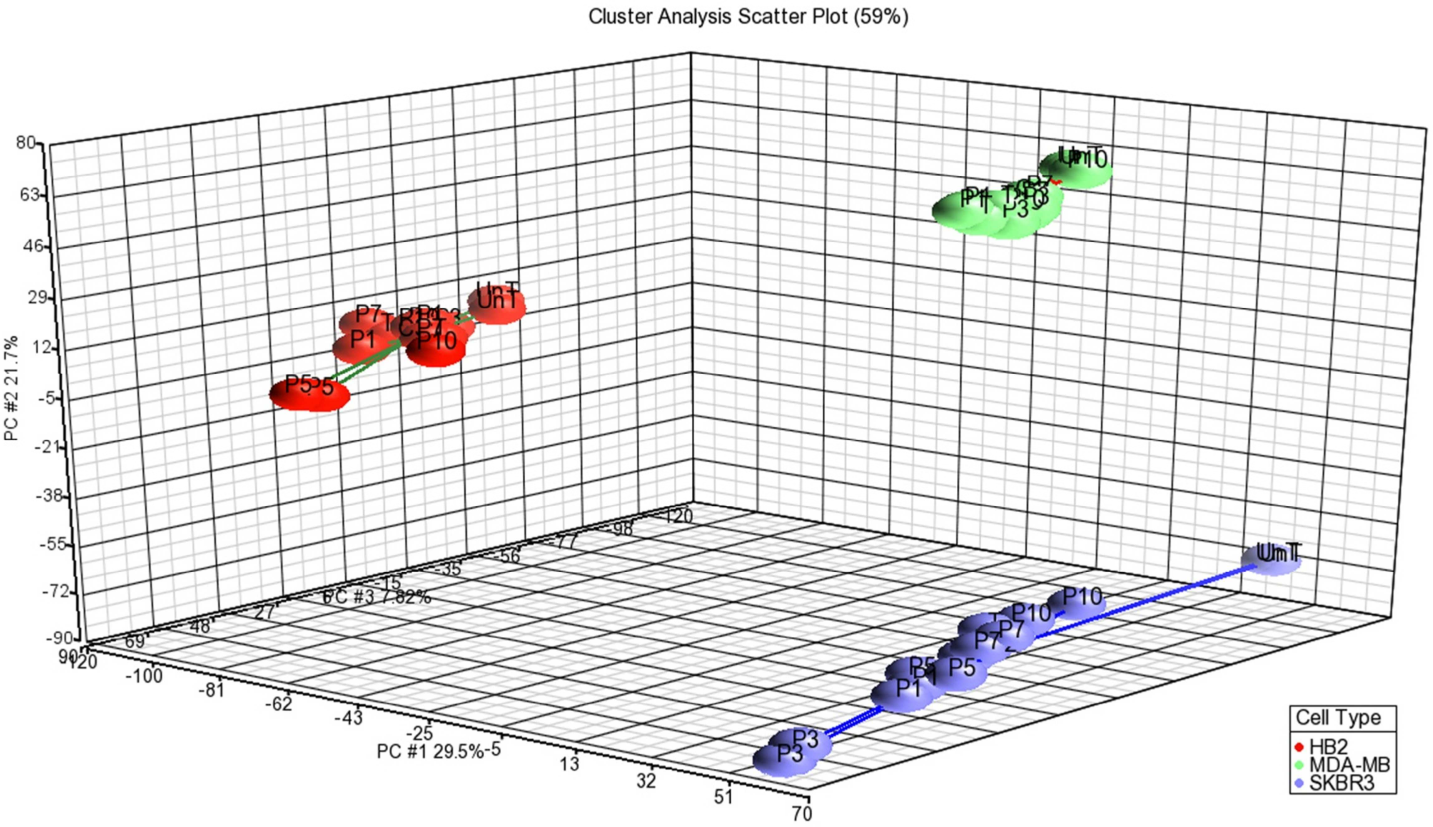

K-means cluster analysis of three cell lines (HB2, MDA-MB231 and SKBR3) based on the Euclidean distance and variance within different treatment passages.

Gene Expression Profiles of HB2
(Breast Epithelial Cell Line)

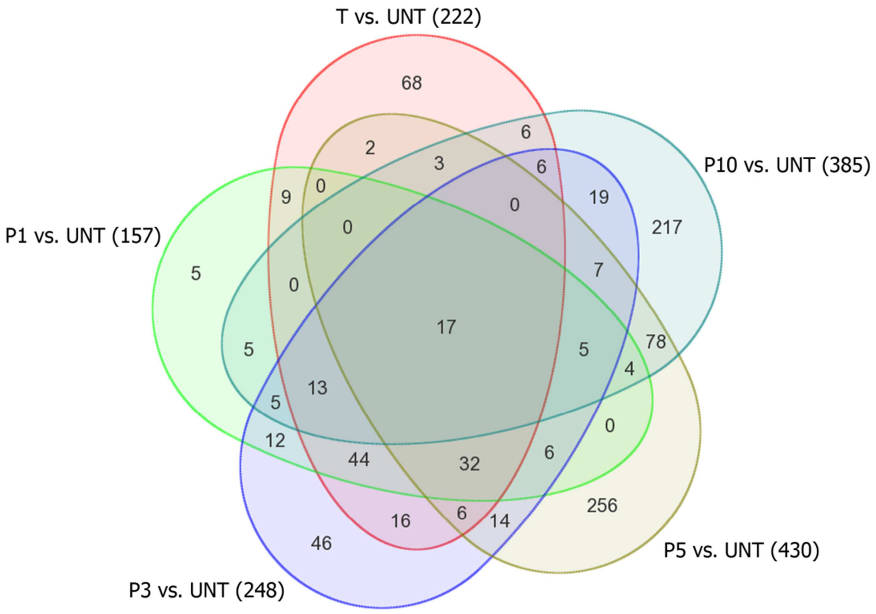

The number of significant up/down-regulated genes within different passages of HB2.

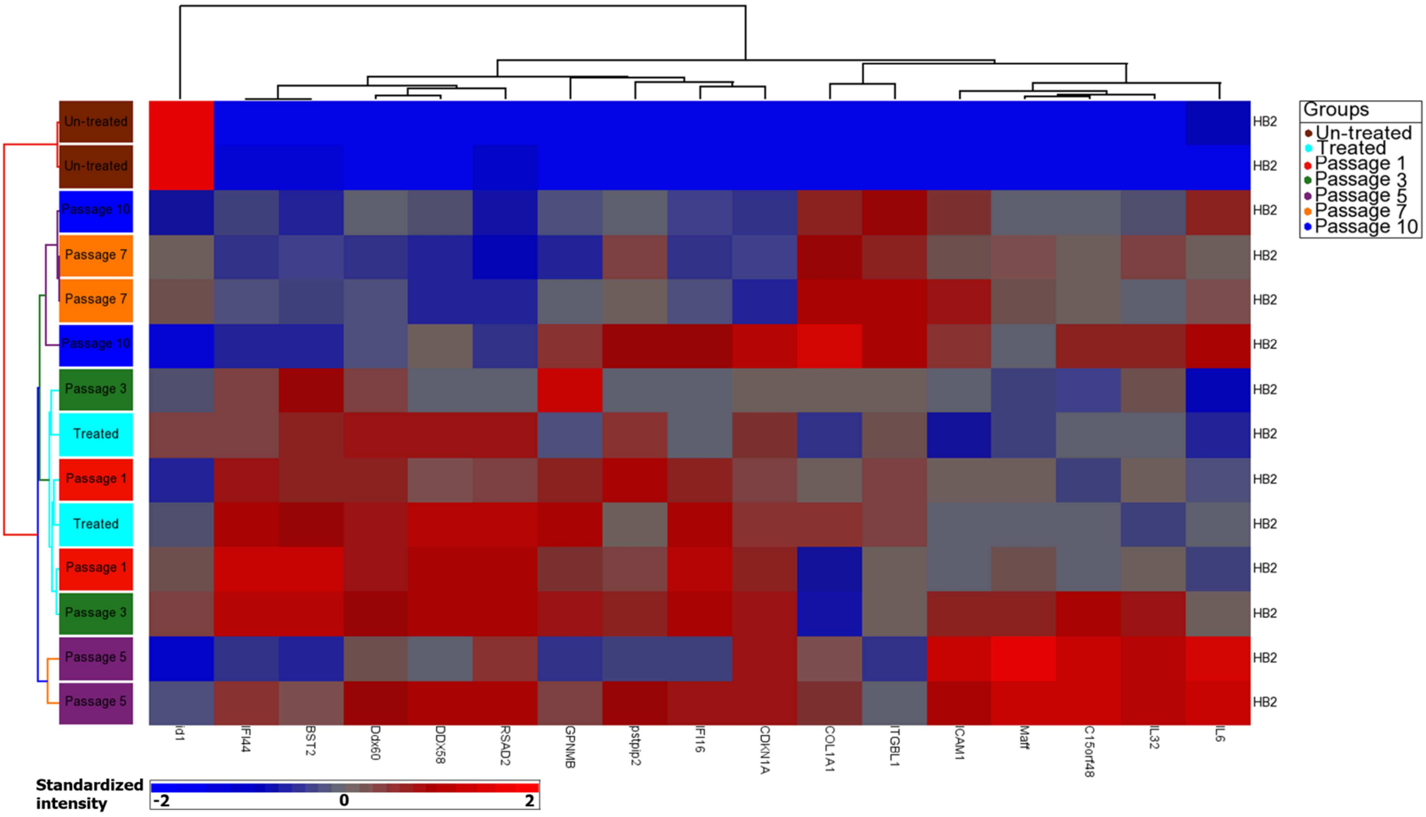

Heatmap shows 17 significant up/down-regulated intersection genes of HB2.

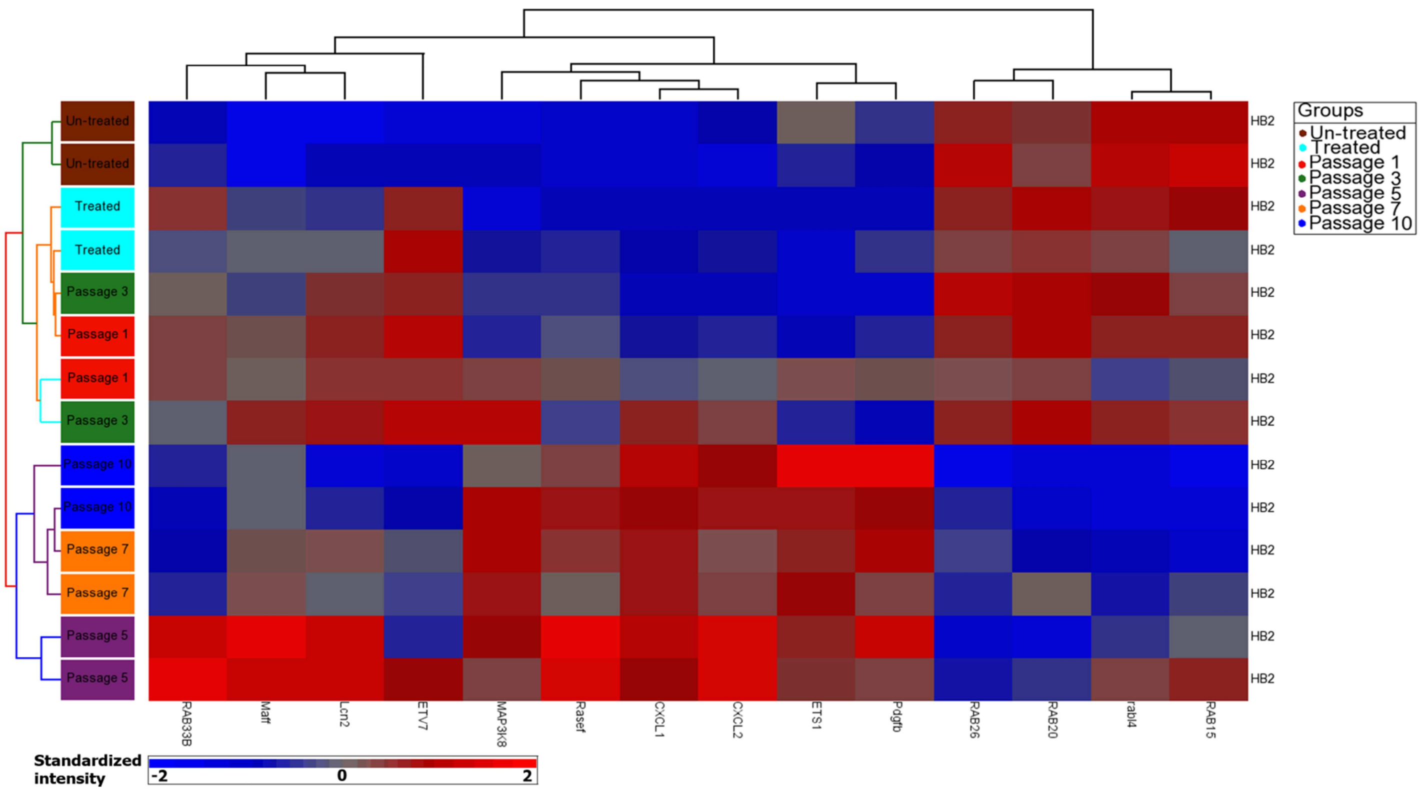

Heatmap shows 14 significant up/down-regulated oncogenes out of 991 union genes in HB2.

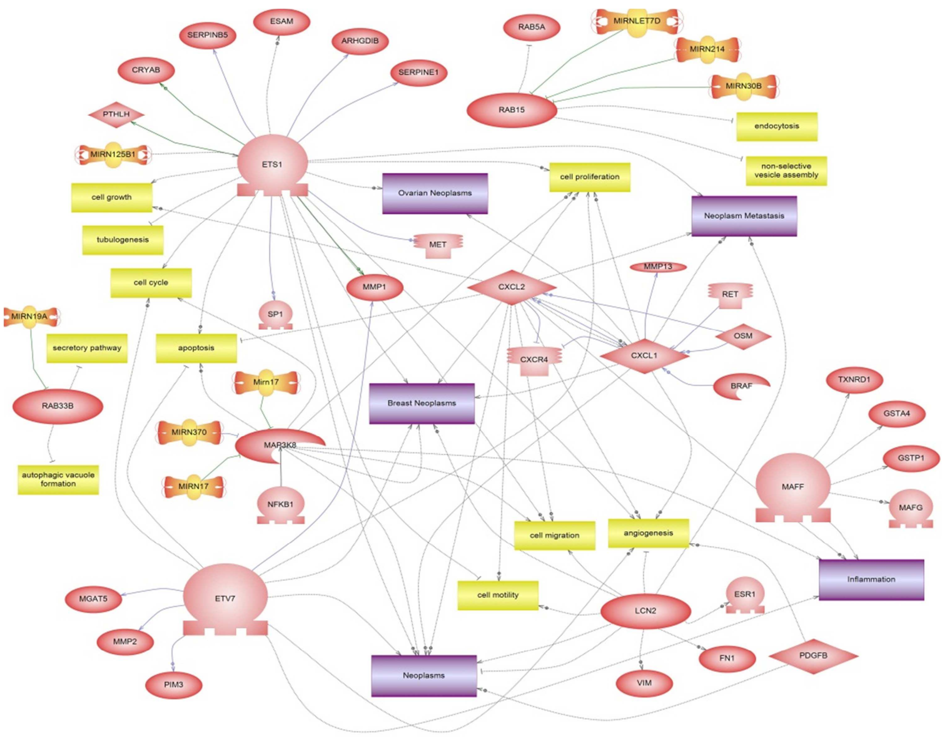

Pathway analysis of 10 out of 14 oncogenes (*ETS1*, *RAB1S*, *CXCL1*, *CXCL2*, *MAFF*, *LCN2*, *PDGFB*, *ETV7*, *RAB33B* and *MAP3K8*) that are linked to breast neoplasms and metastasis. These genes are shown as significant dysregulated during the different passages of HB2.

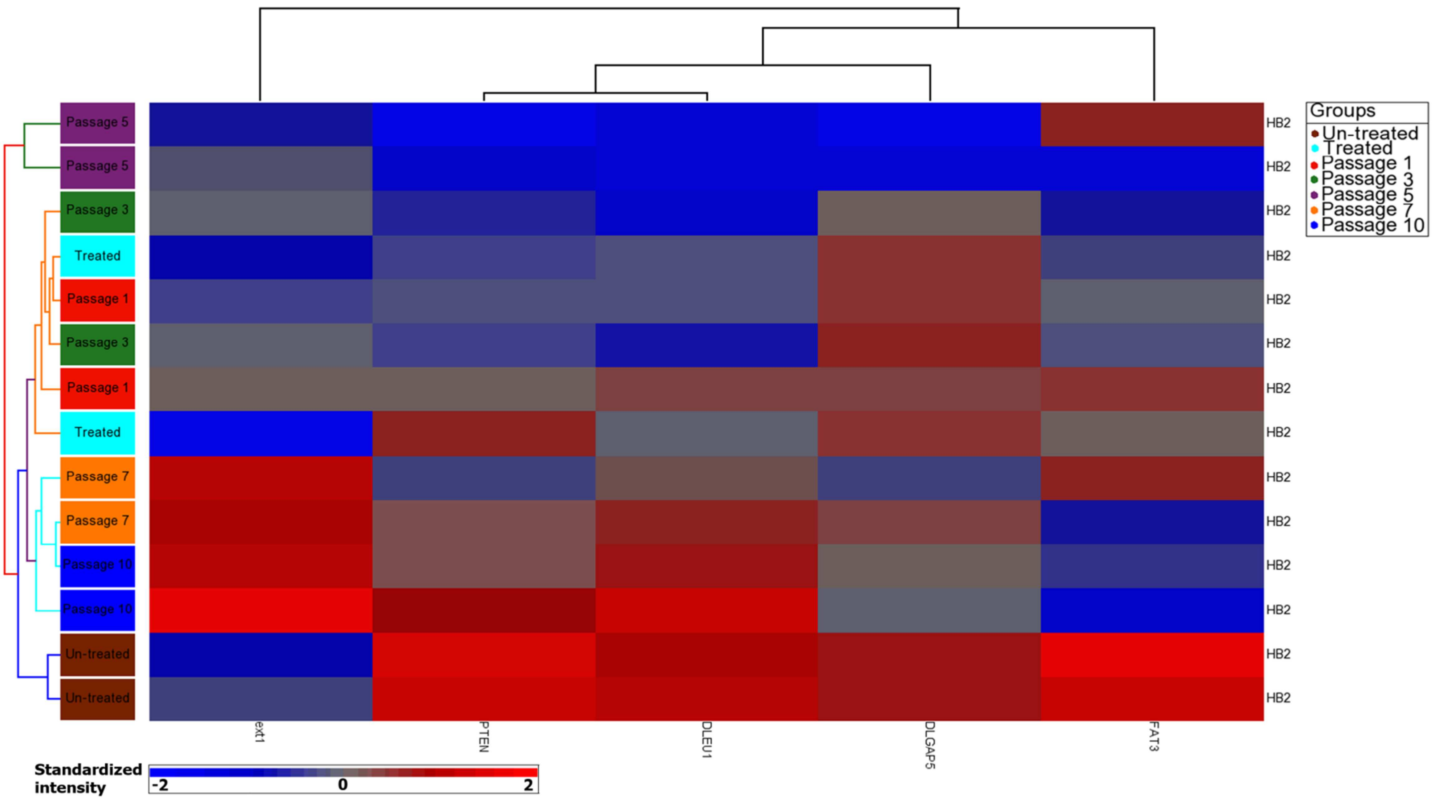

Heatmap shows five significant up/down-regulated tumor suppressor genes out of 991 union genes in HB2.

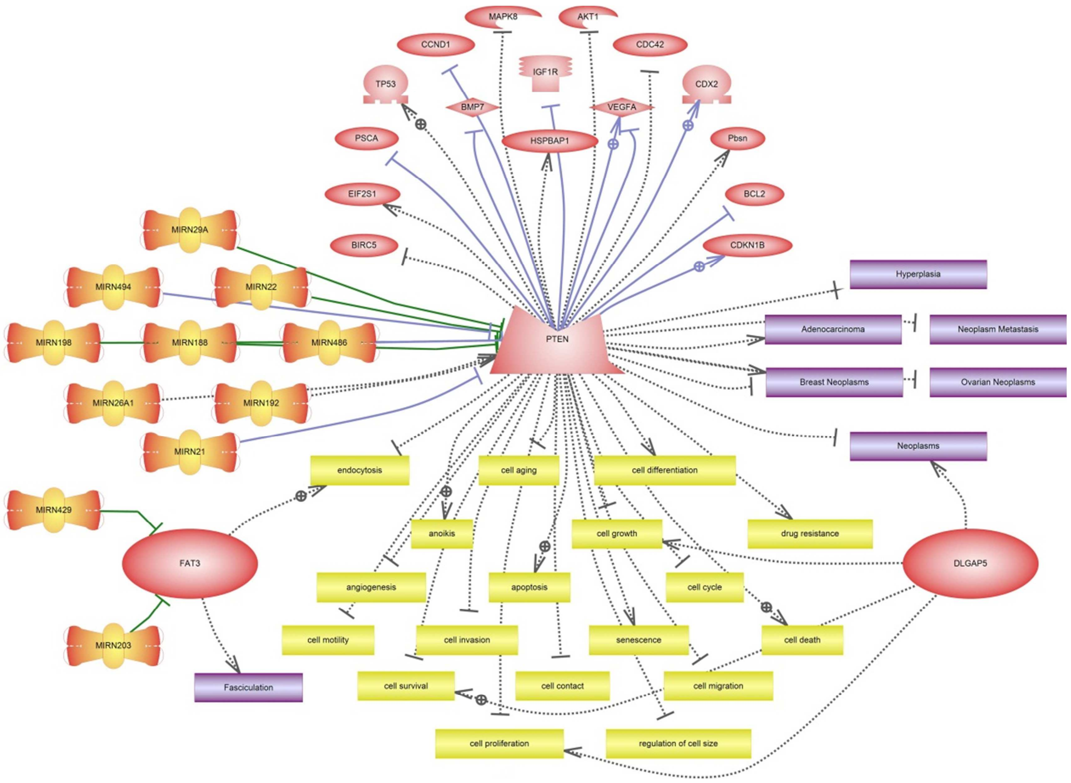

Pathway analysis of three out of five tumor suppressor genes (*FAT3*, *PTEN* and *DLGAP5*) that are linked to breast neoplasms and metastasis. These are significant dysregulated genes during the different passages of HB2.

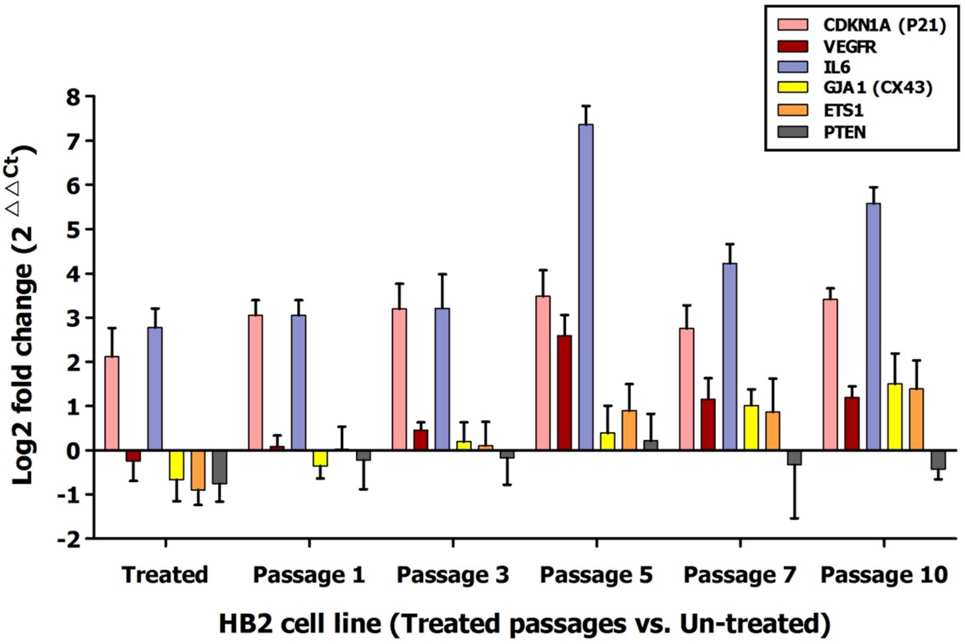

Expression level of six cancer related genes after treatment using qPCR. Four genes (*CDKN1A*, *VEGFR*, *IL6* and *GJA1*) as intersection genes in the three analyzed cell lines, *ETS1* and *PTEN* as examples of significantly changed oncogenes and tumor suppressor genes respectively.

Gene Expression Profiles of MDA-MB231
(Highly Aggressive Breast Cancer Cell Line)

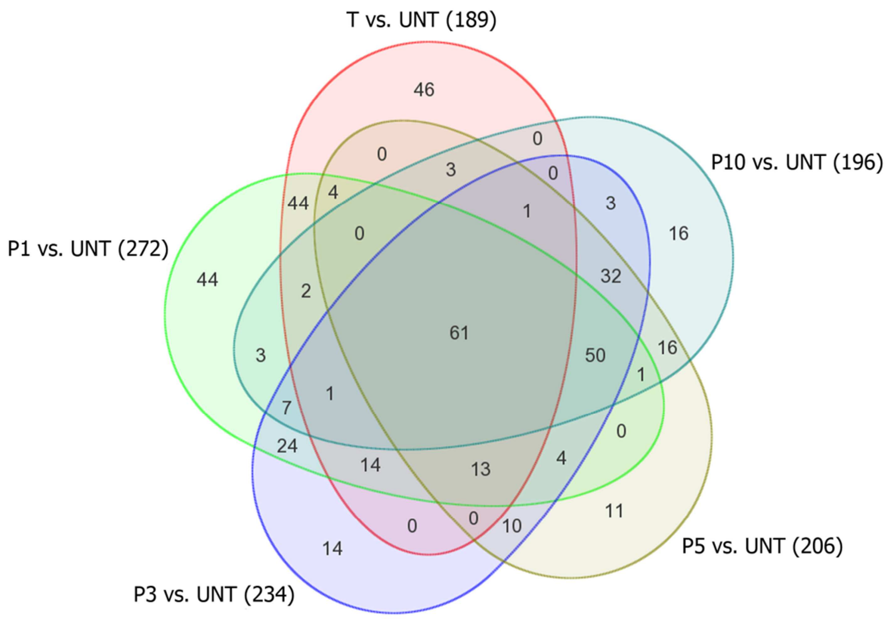

The number of significant up/down-regulated genes within different passages of MDA-MB231.

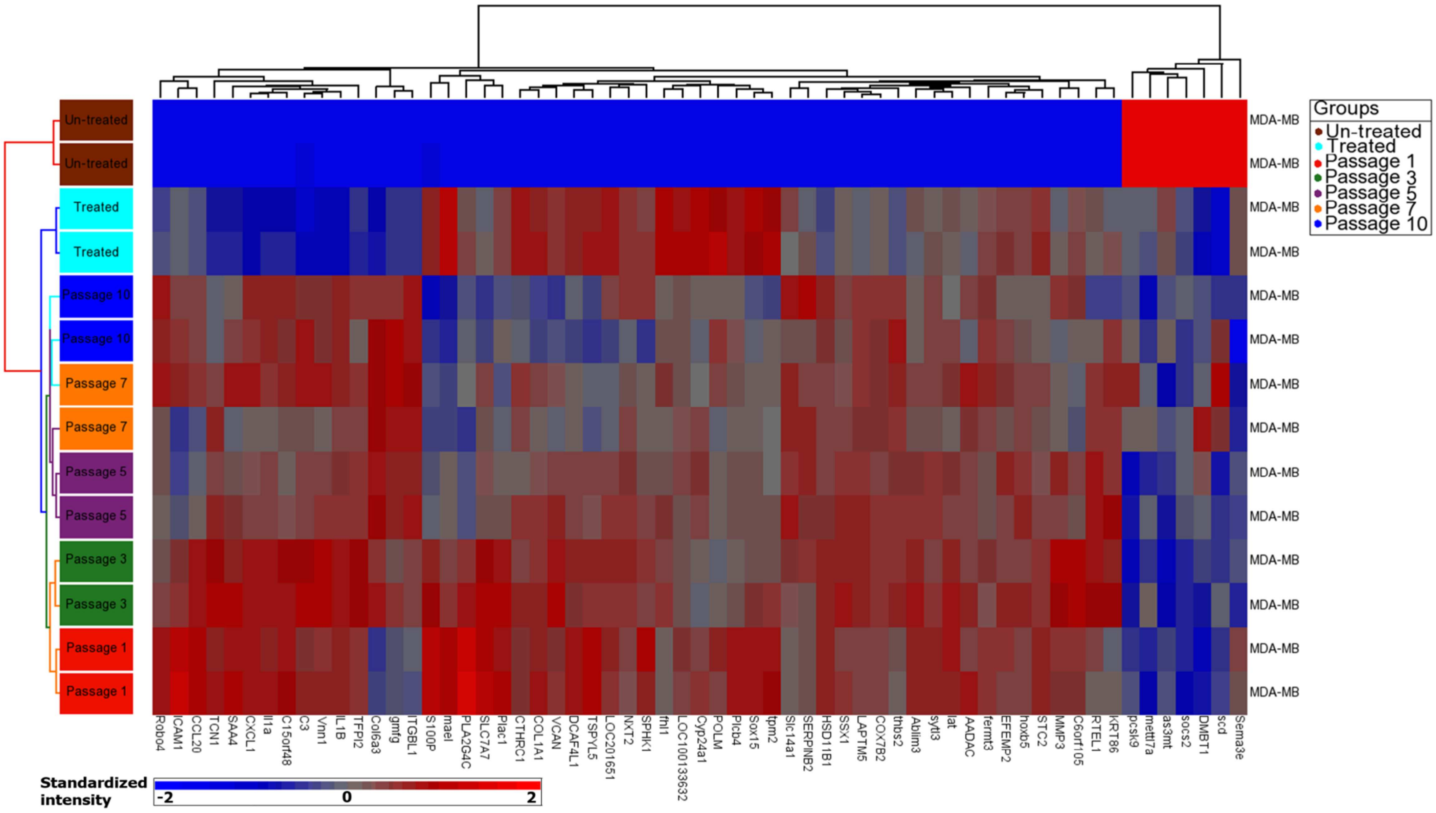

Heatmap shows 61 significant up/down-regulated intersection genes of MDA-MB231.

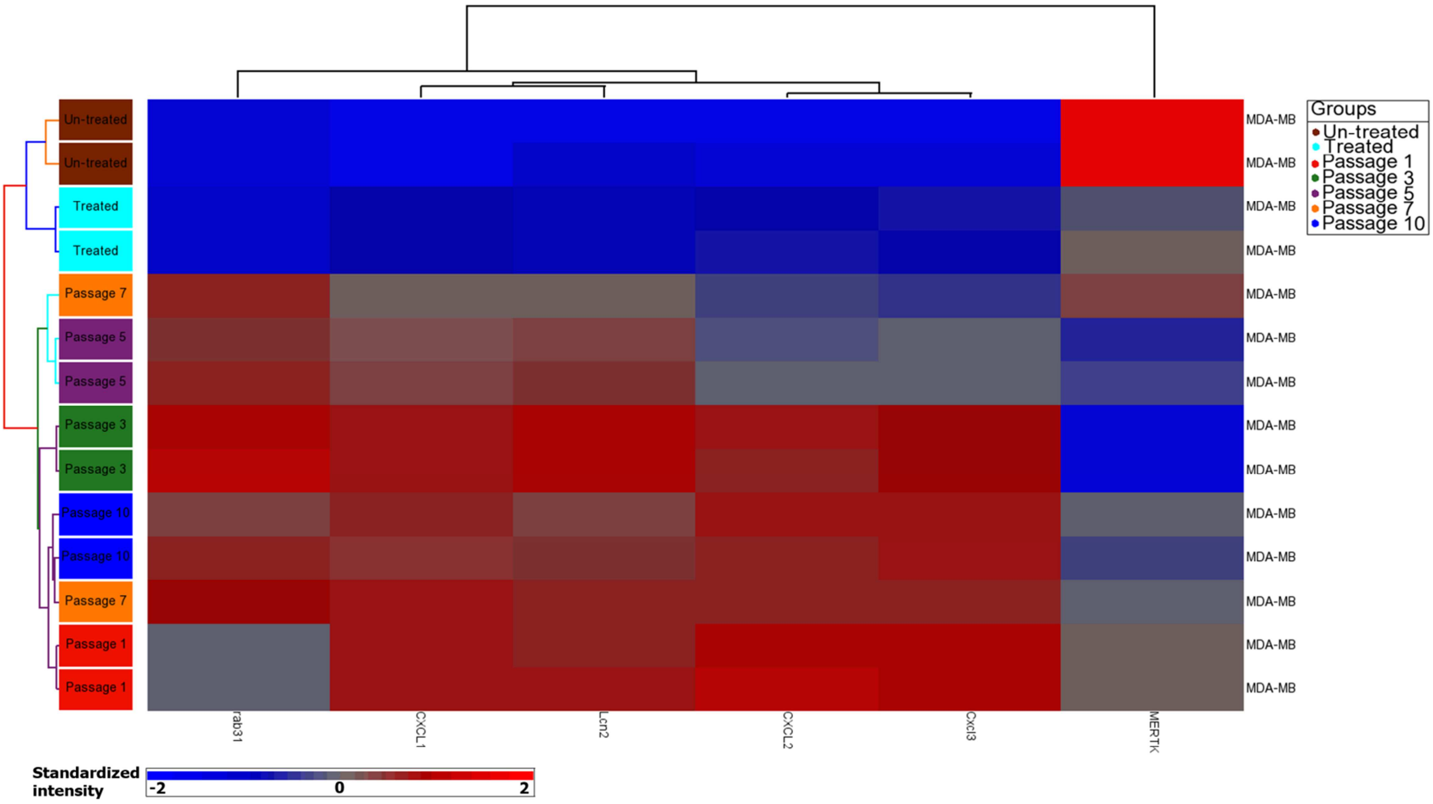

Heatmap shows six significant up/down-regulated oncogenes out of 431 union genes in MDA-MB231.

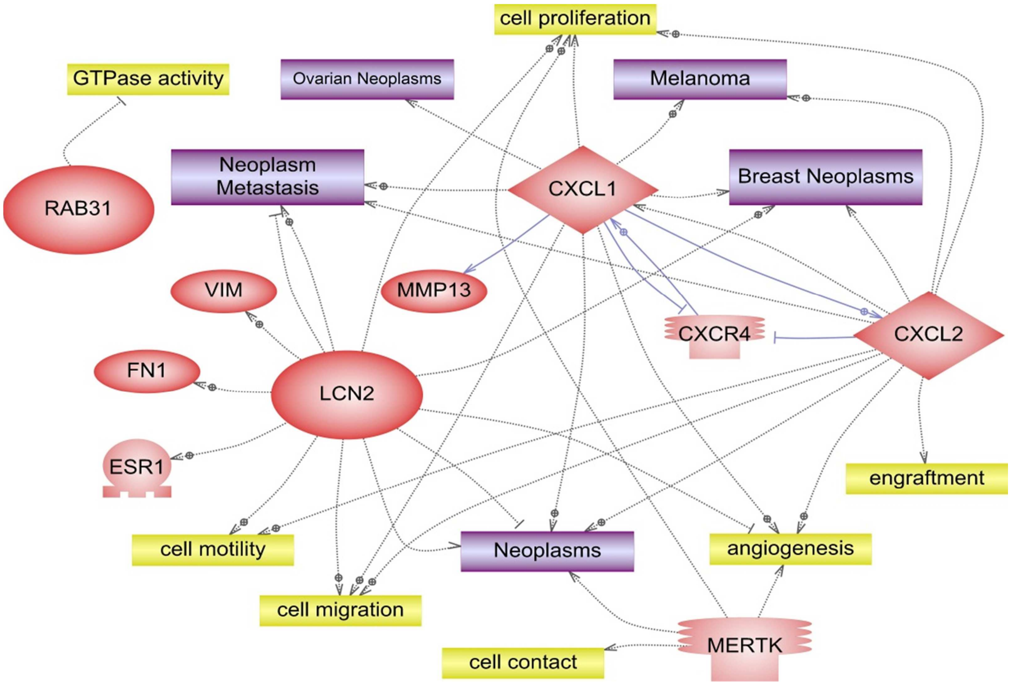

Pathway analysis of five out of six oncogenes (*MERTK*, *RAB31*, *CXCL1*, *CXCL2* and *LCN2*) that are linked to breast neoplasms and metastasis. These genes are shown as significant dysregulated during the different passages of MDA-MB231.

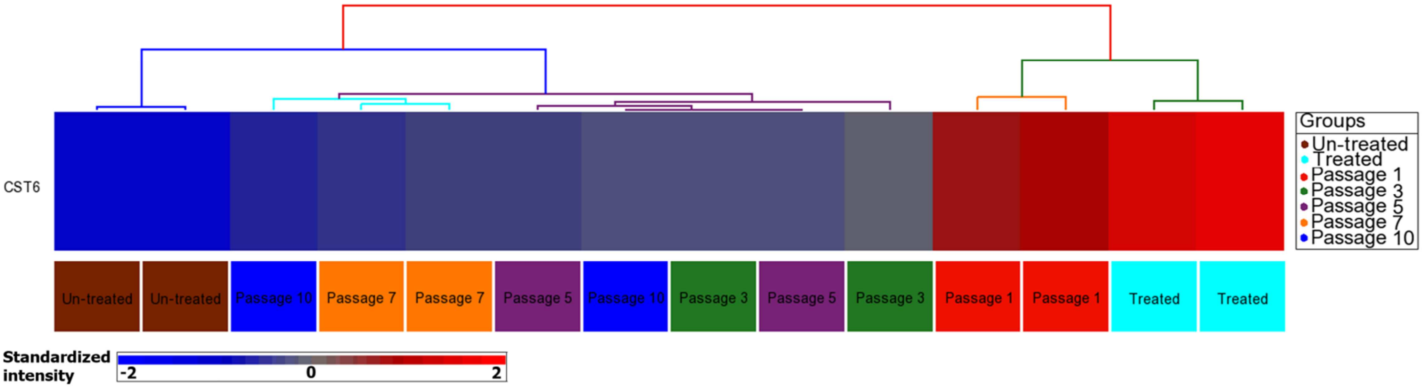

Heatmap shows a significant up/down-regulated tumor suppressor gene out of 431 union genes in MDA-MB231.

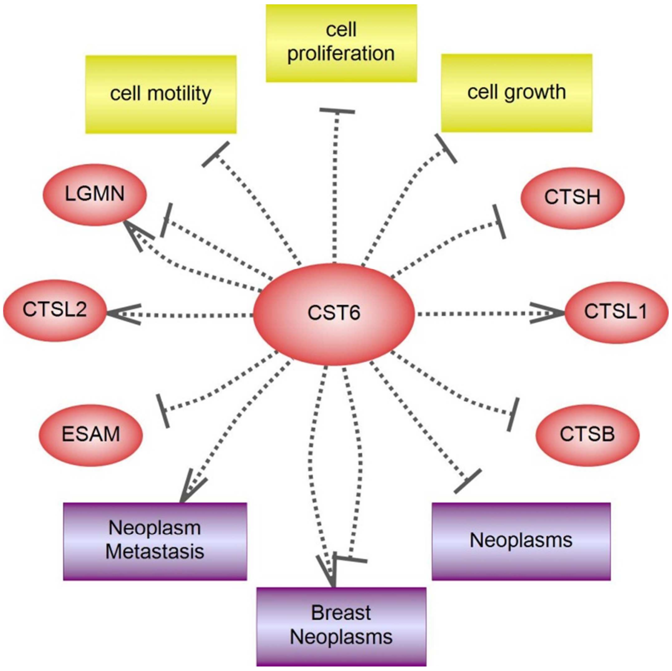

Pathway analysis of *CST6* tumor suppressor gene that is linked to breast neoplasms and metastasis. This gene is marked as significant dysregulated during the different passages of MDA-MB231.

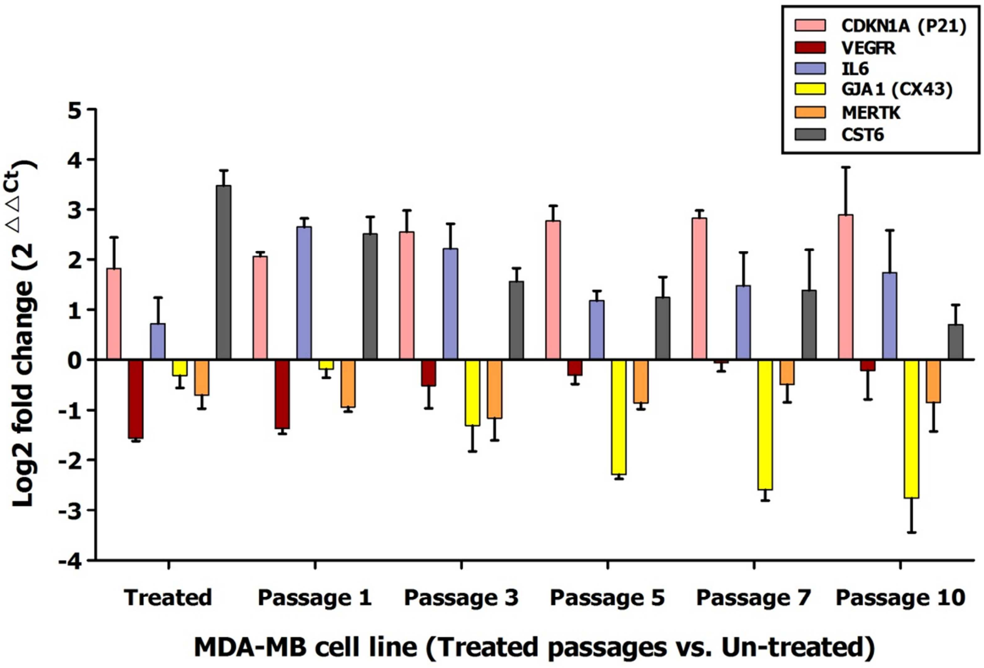

Expression level of six cancer related genes after treatment using qPCR. Four genes (*CDKN1A*, *VEGFR*, *IL6* and *GJA1*) as intersection genes in the three analyzed cell lines, *MERTK* and *CST6* as examples of significantly changed oncogenes and tumor suppressor genes respectively.

Gene Expression Profiles of SKBR3
(Non-aggressive Breast Cancer Cell Line)

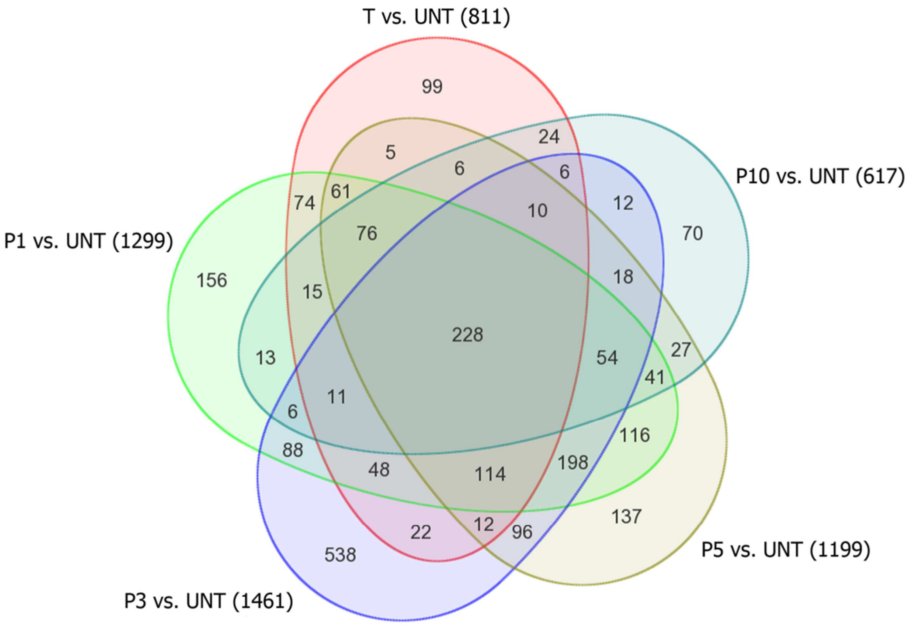

The number of significant up/down-regulated genes within different passages of SKBR3.

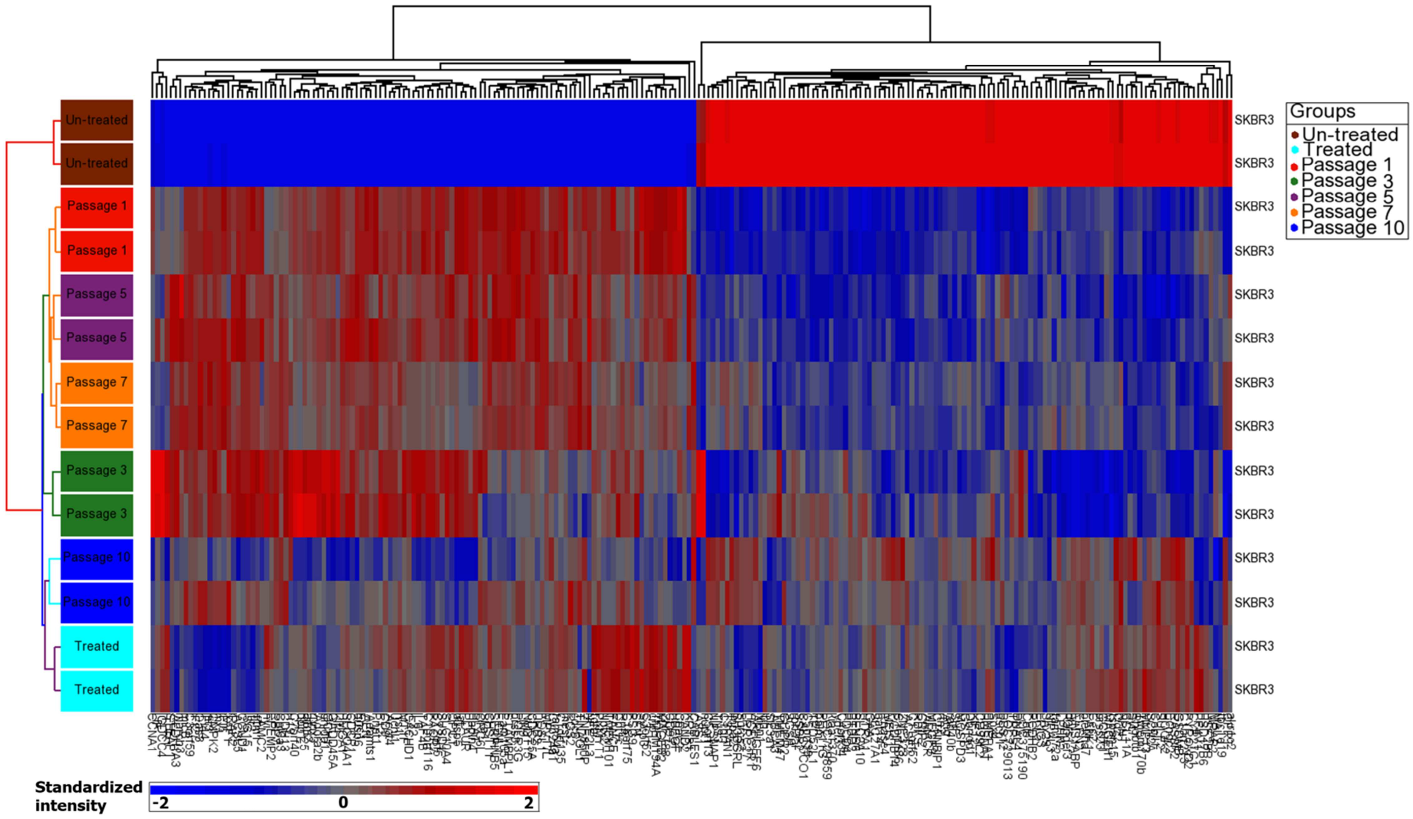

Heatmap shows 228 significant up/down-regulated intersection genes of SKBR3.

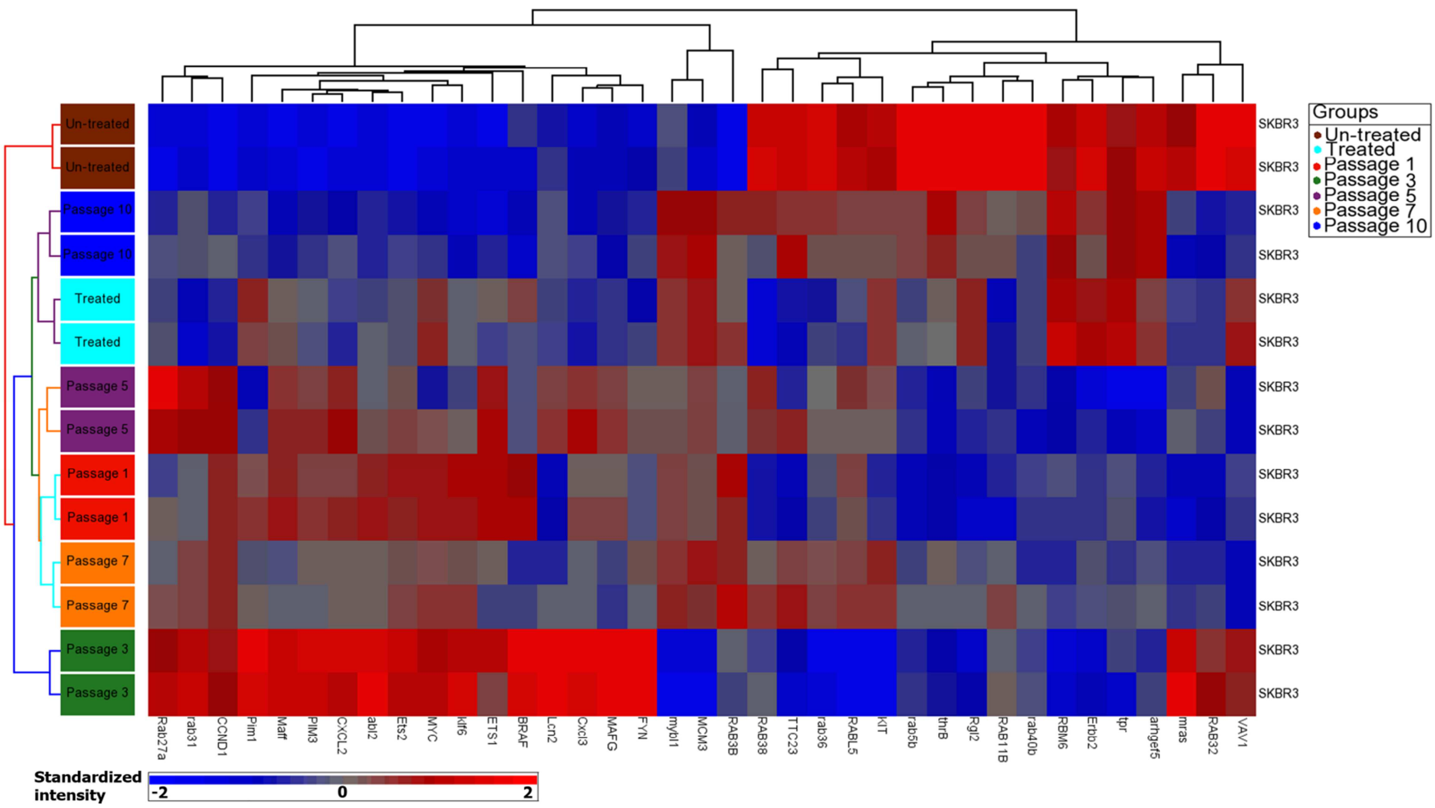

Heatmap shows 37 significant up/down-regulated oncogenes out of 2406 union genes in SKBR3.

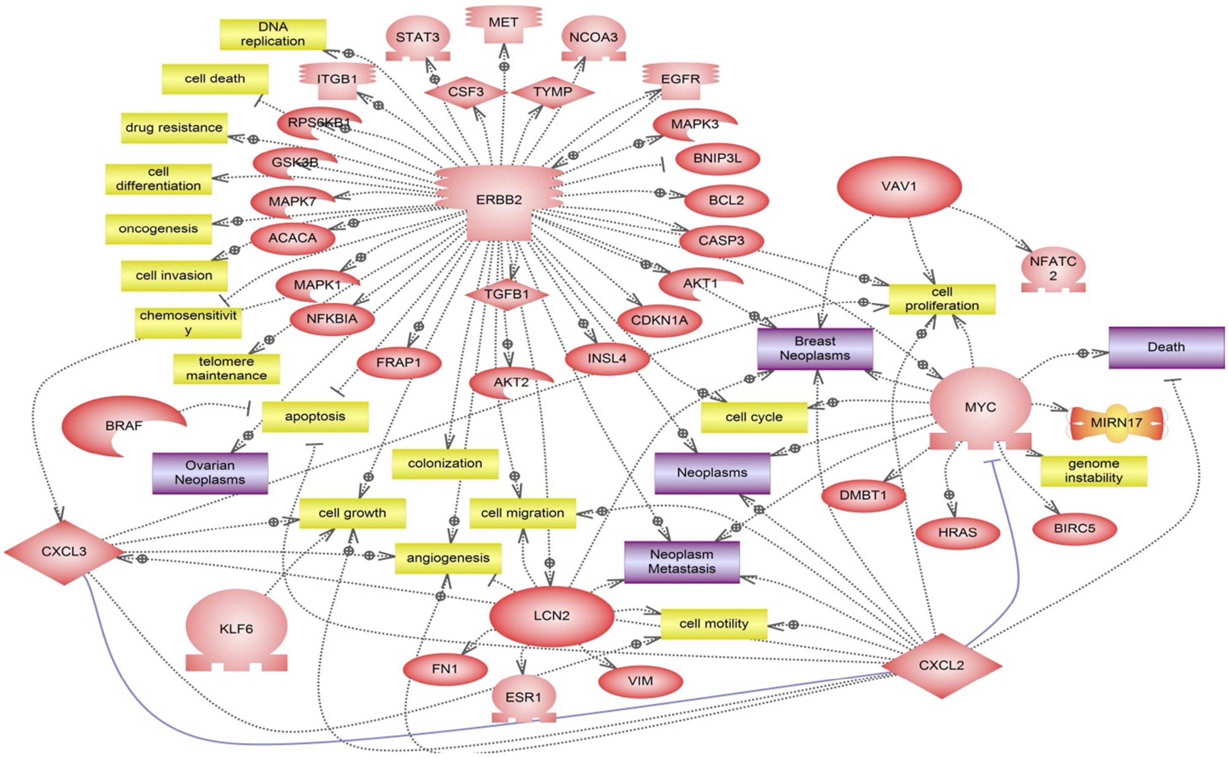

Pathway analysis of eight out of 37 oncogenes (*CXCL2*, *CXCL3*, *ERBB2*, *VAV1*, *MYC*, *BRAF*, *KLF6* and *LCN2*) that are linked to breast neoplasms and metastasis. These genes are shown as significant dysregulated during the different passages of SKBR3.

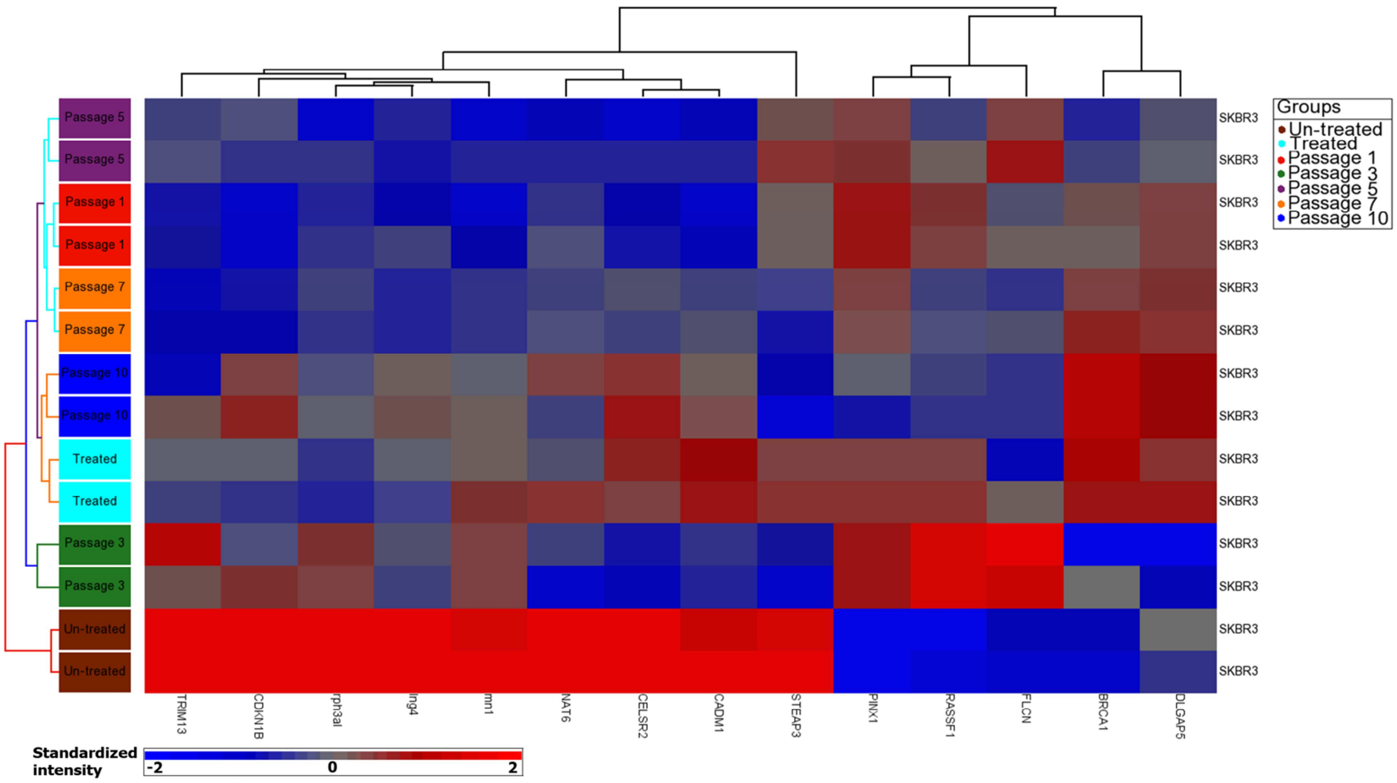

Heatmap shows 14 significant up/down-regulated tumor suppressor genes out of 2406 union genes in SKBR3.

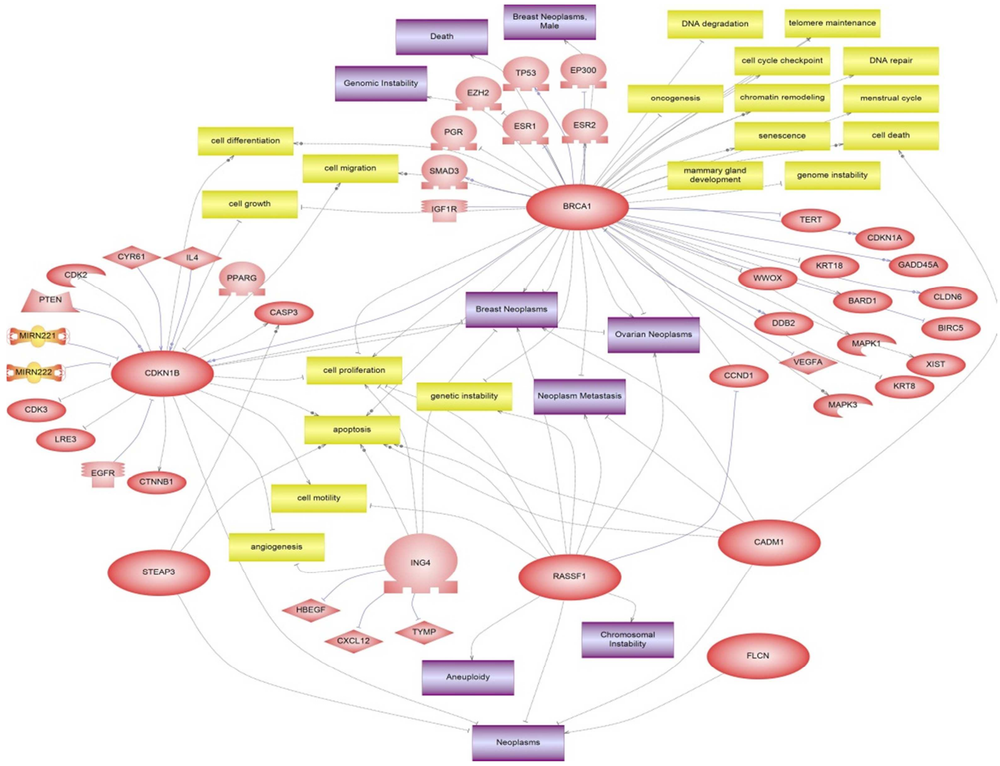

Pathway analysis of seven out of 14 tumor suppressor genes (*BRCA1*, *CDKN1B*, *STEAP3*, *ING4*, *RASSF1*, *CADM1* and *FLCN*) that are linked to breast neoplasms and metastasis. These genes are shown as significant dysregulated during the different passages of SKBR3.

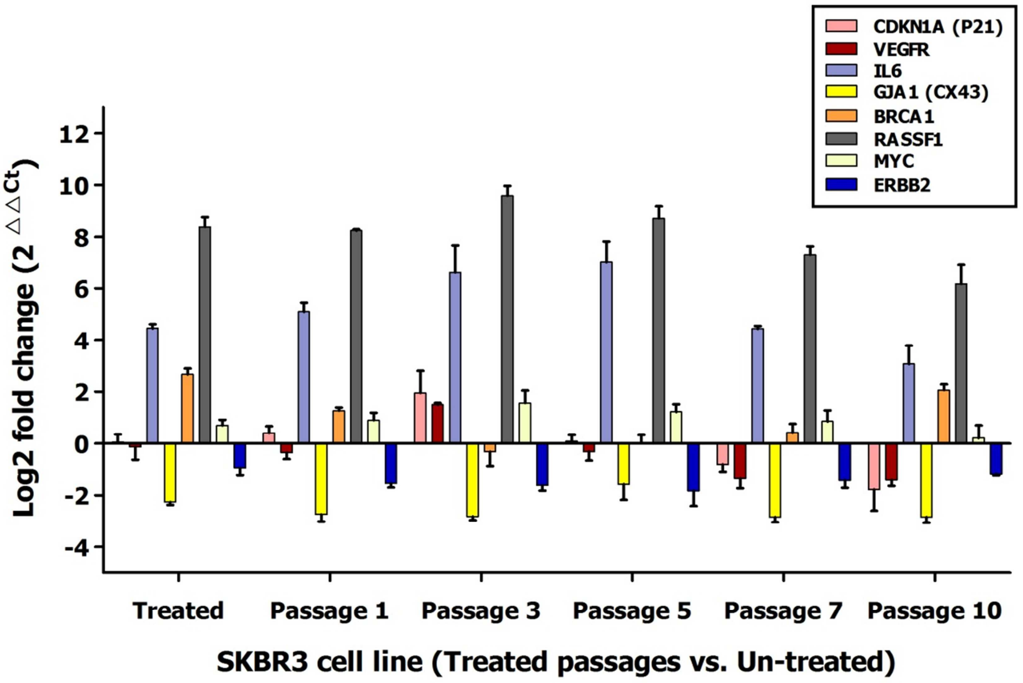

Expression level of eight cancer related genes after treatment using qPCR. Four genes (*CDKN1A*, *VEGFR*, *IL6* and *GJA1*) as intersection genes in the three analyzed cell lines, *MYC* and *ERBB2* as examples of significantly changed oncogenes, *BRCA1* and *RASSF1* as an example of tumor suppressor genes.
