# Supplementary material for: Integrated Epigenetics of Human Breast Cancer: Synoptic Investigation of Targeted Genes, MicroRNAs and Proteins upon Demethylation Treatment
Source: PLoS One. 2011 Nov 4;6(11):e27355. doi: 10.1371/journal.pone.0027355 (PMC3208622; doi:10.1371/journal.pone.0027355)
Supplement: Dataset S3 — MicroRNA Expression Profiles. PCA analysis of studied cell lines (HB2, MDA-MB231 and SKBR3)K-means cluster analysis of three cell lines based on the Euclidean distance and variance within different treatment passagesThe number of significant up/down-regulated miRNAs within different passagesPathway analysis of miRNAs that are linked to neoplasms, metastasis or carcinogenesisHeatmaps of significant up/down-regulated union miRNAsExpression level of important cancer related miRNAs using qPCR (PDF) [file pone.0027355.s003.pdf]

Dataset S3

MicroRNA Expression Profiles

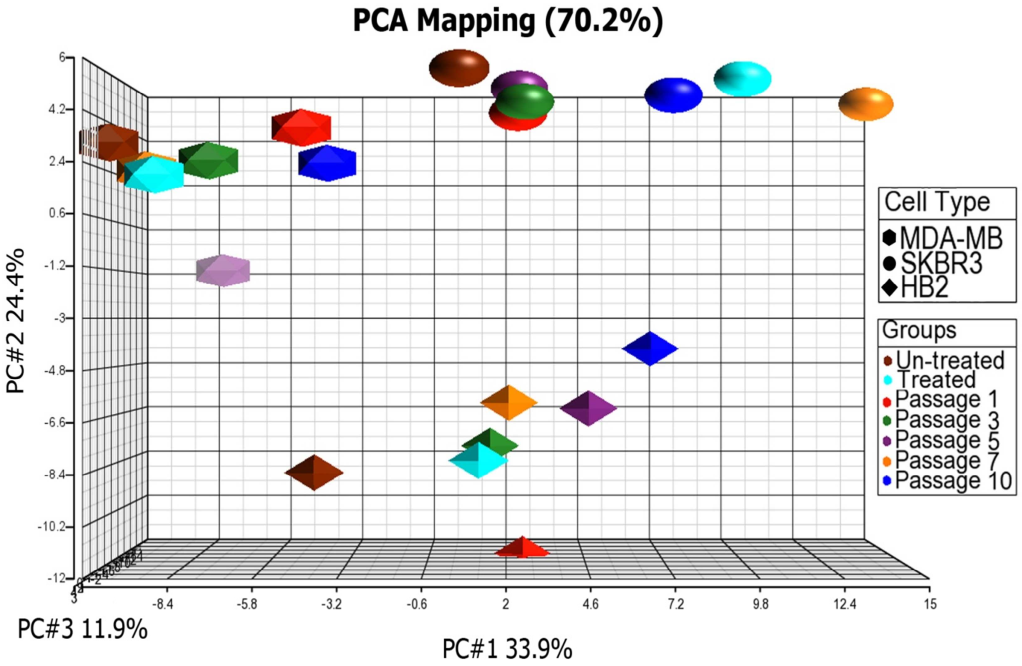

PCA analysis of three studied cell lines (HB2, MDA-MB231 and SKBR3).

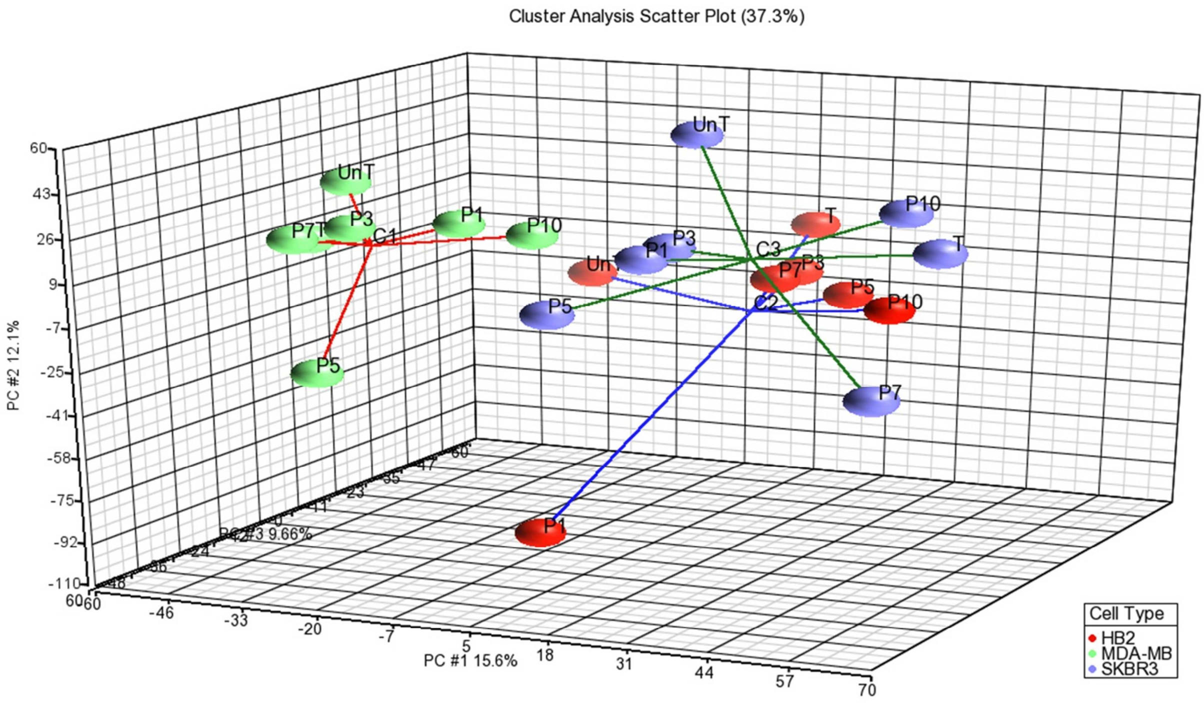

K-means cluster analysis of three cell lines (HB2, MDA-MB231 and SKBR3) based on the Euclidean distance and variance within different treatment passages.

MicroRNA Expression Profiles of HB2
(Breast Epithelial Cell Line)

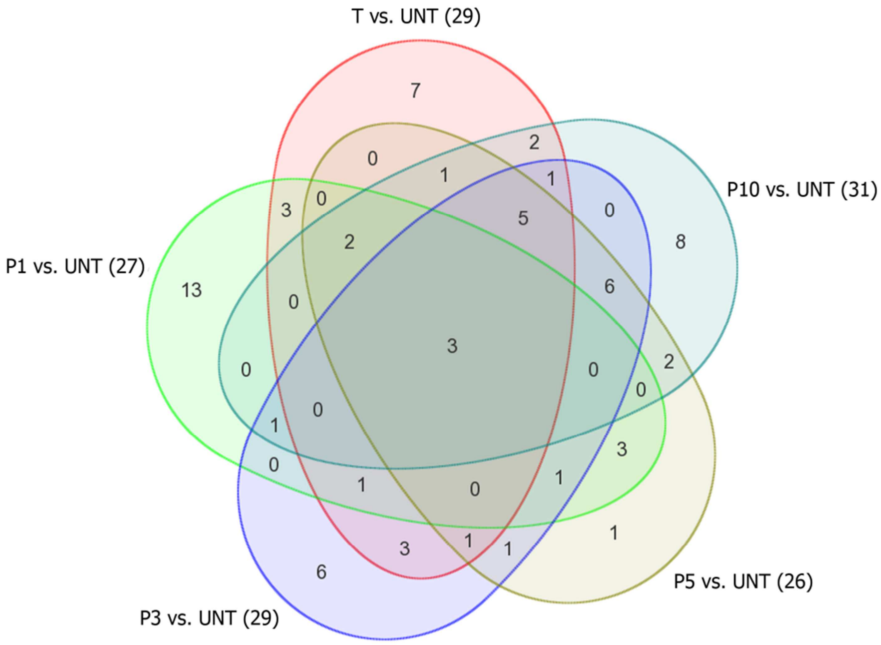

The number of significant up/down-regulated miRNAs within different passages of HB2.

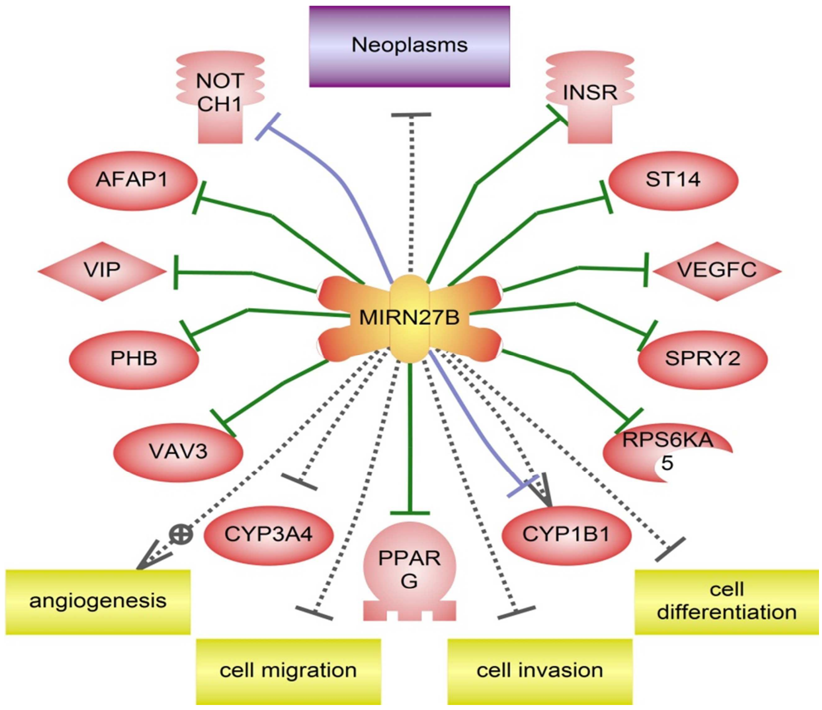

Pathway analysis of a miRNA out of three intersection miRNAs in HB2 that is linked to neoplasms, metastasis or carcinogenesis.

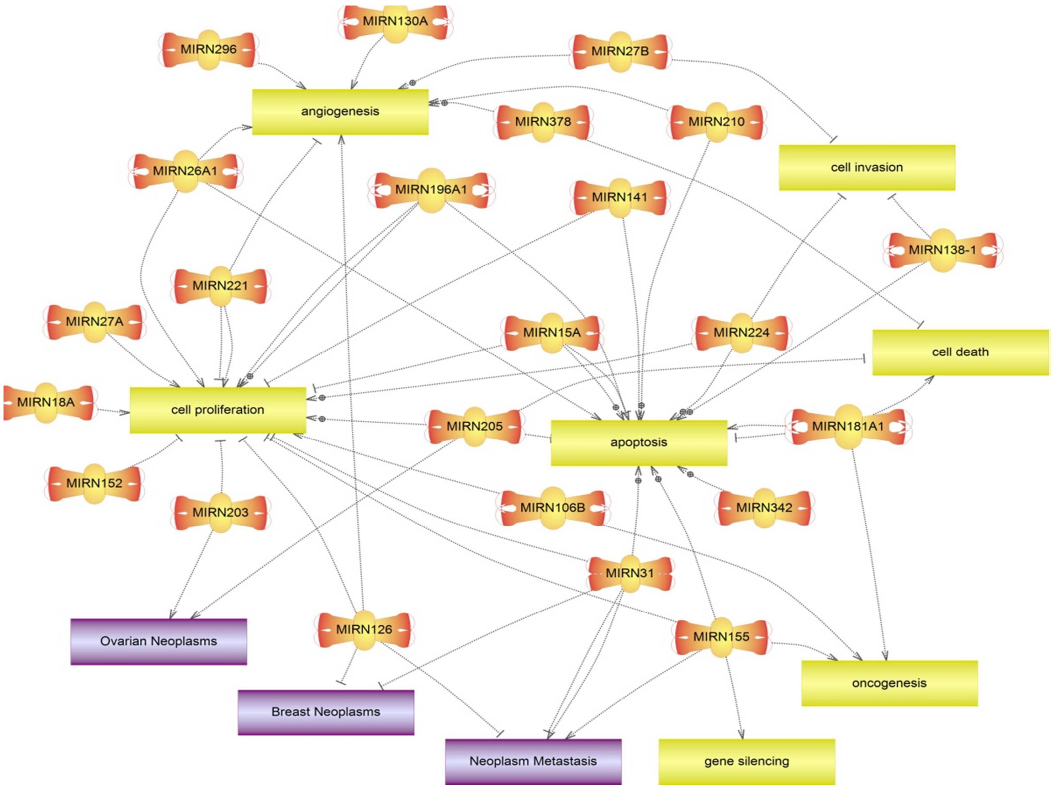

Pathway analysis of 23 union miRNAs in HB2 that are linked to neoplasms, metastasis or carcinogenesis.

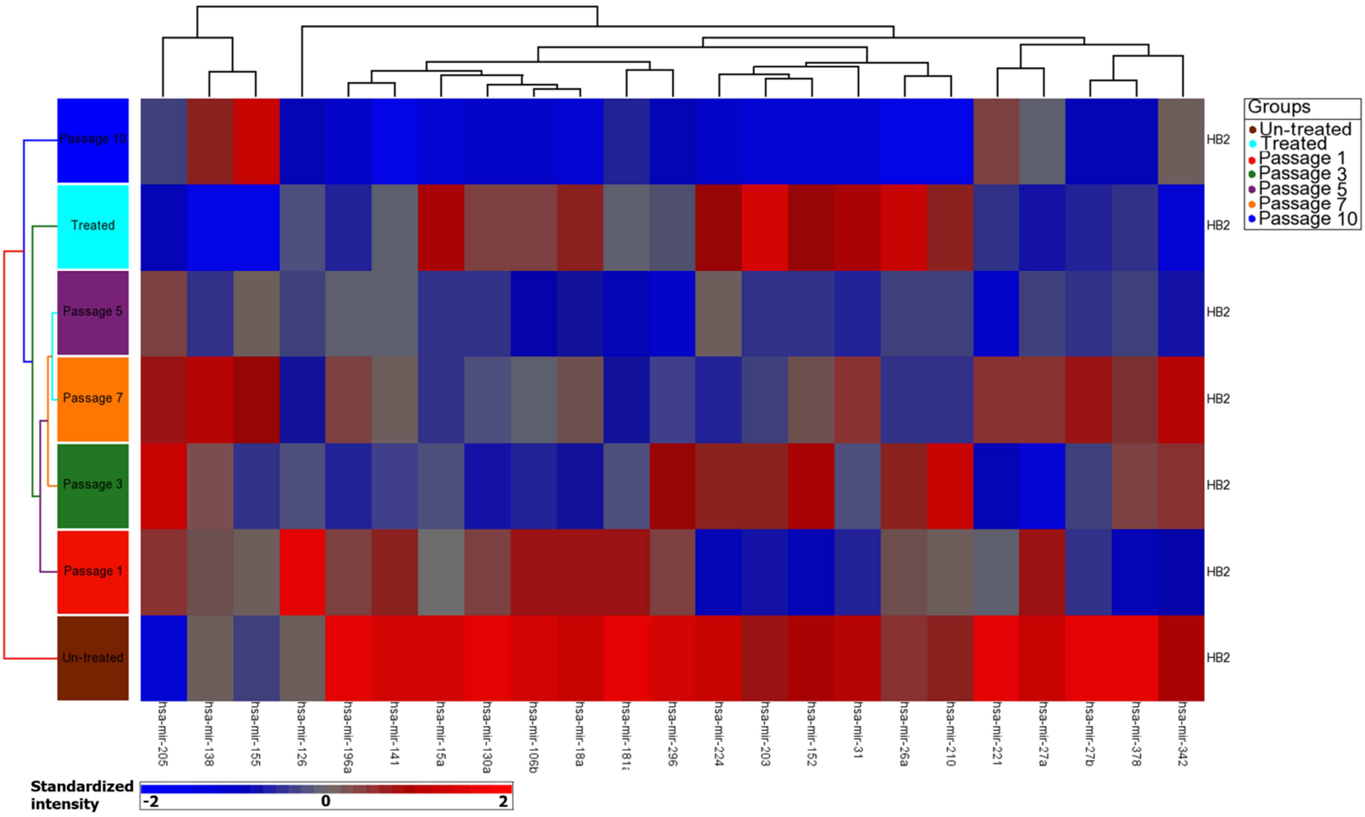

Heatmap shows 23 significant up/down-regulated union miRNAs in HB2.

MicroRNA Expression Profiles of MDA-MB231
(Highly Aggressive Breast Cancer Cell Line)

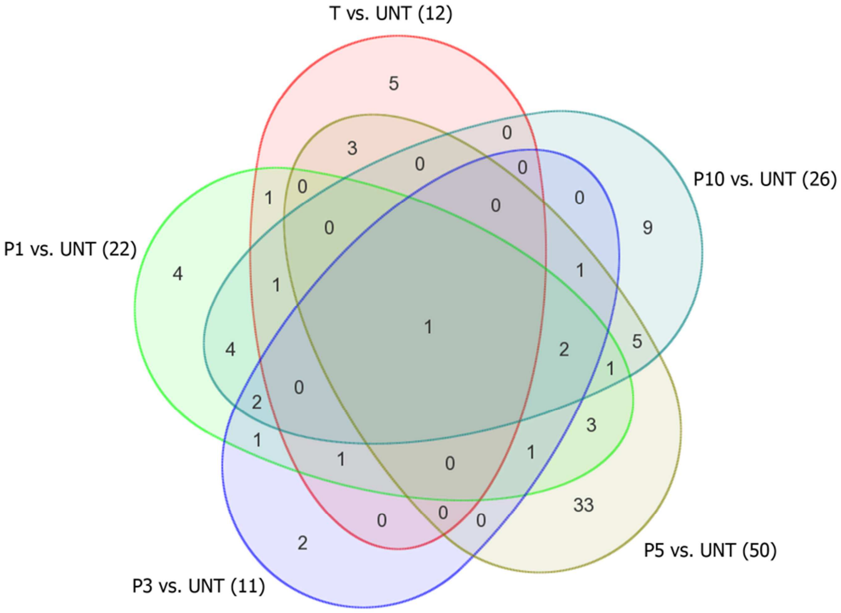

The number of significant up/down-regulated miRNAs within different passages of MDA-MB231.

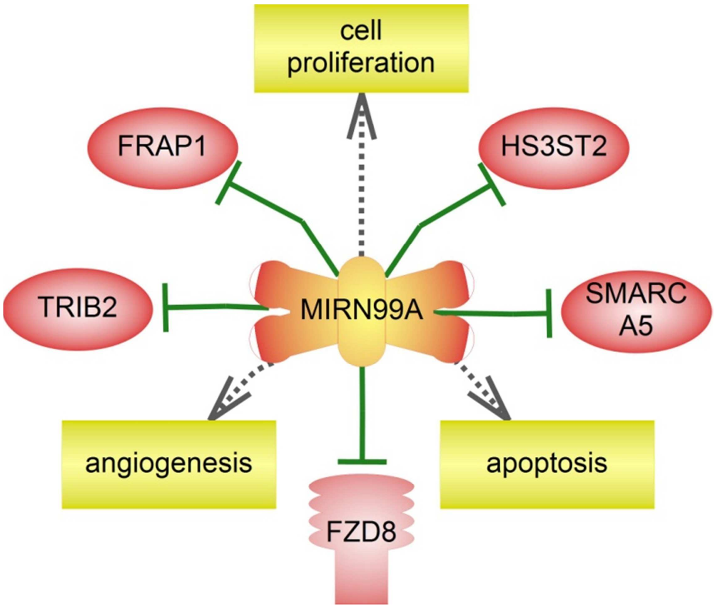

Pathway analysis of an intersection miRNA in MDA-MB231 that is linked to neoplasms, metastasis or carcinogenesis.

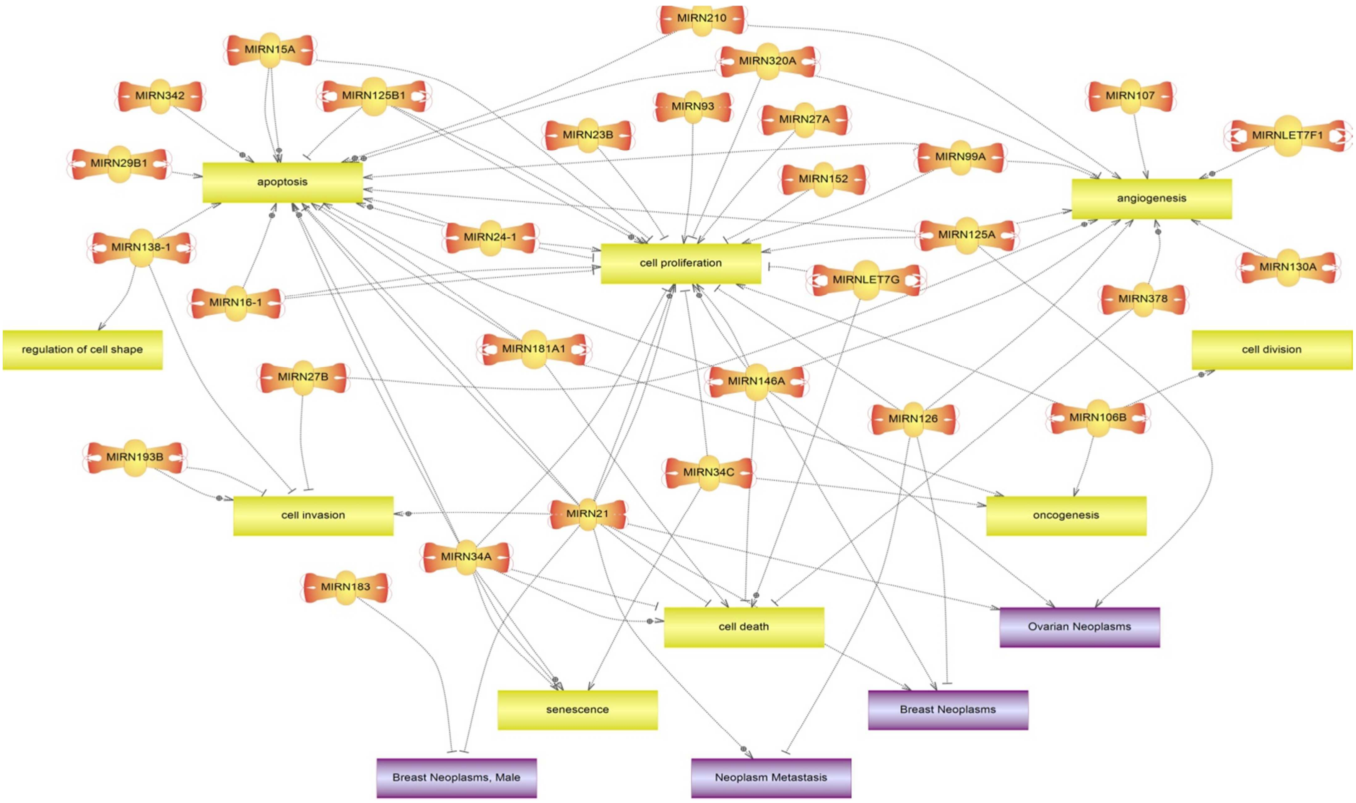

Pathway analysis of 30 union miRNAs in MDA-MB231 that are linked to neoplasms, metastasis or carcinogenesis.

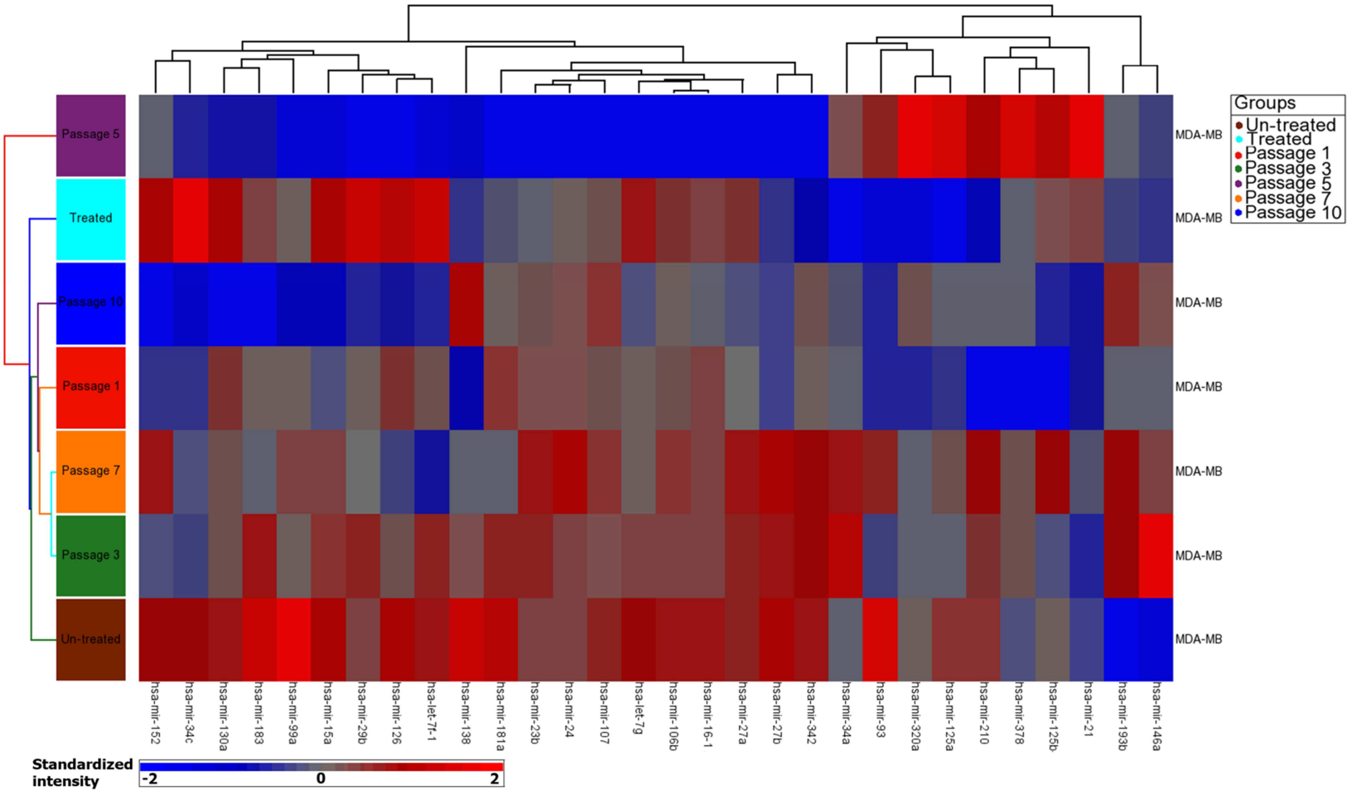

Heatmap shows 30 significant up/down-regulated union miRNAs in MDA-MB231.

MicroRNA Expression Profiles of SKBR3
(Non-aggressive Breast Cancer Cell Line)

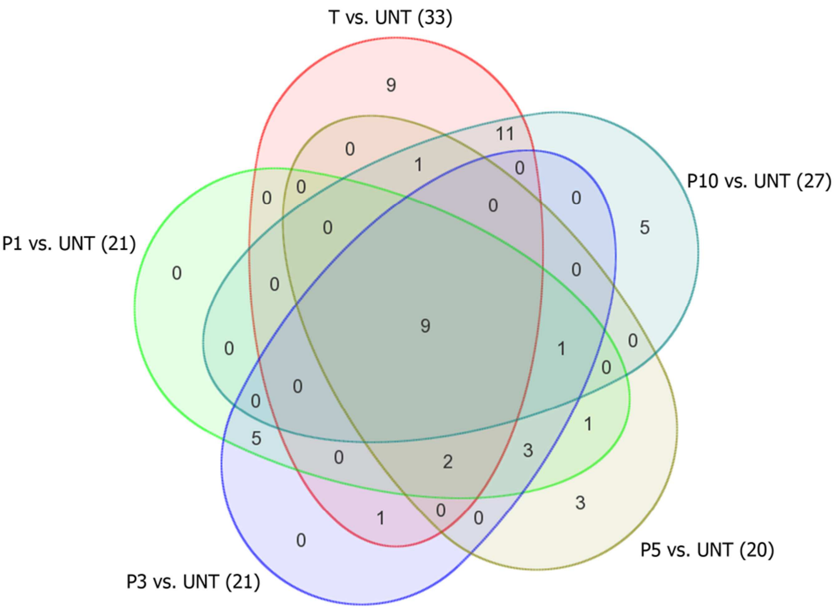

The number of significant up/down-regulated miRNAs within different passages of SKBR3.

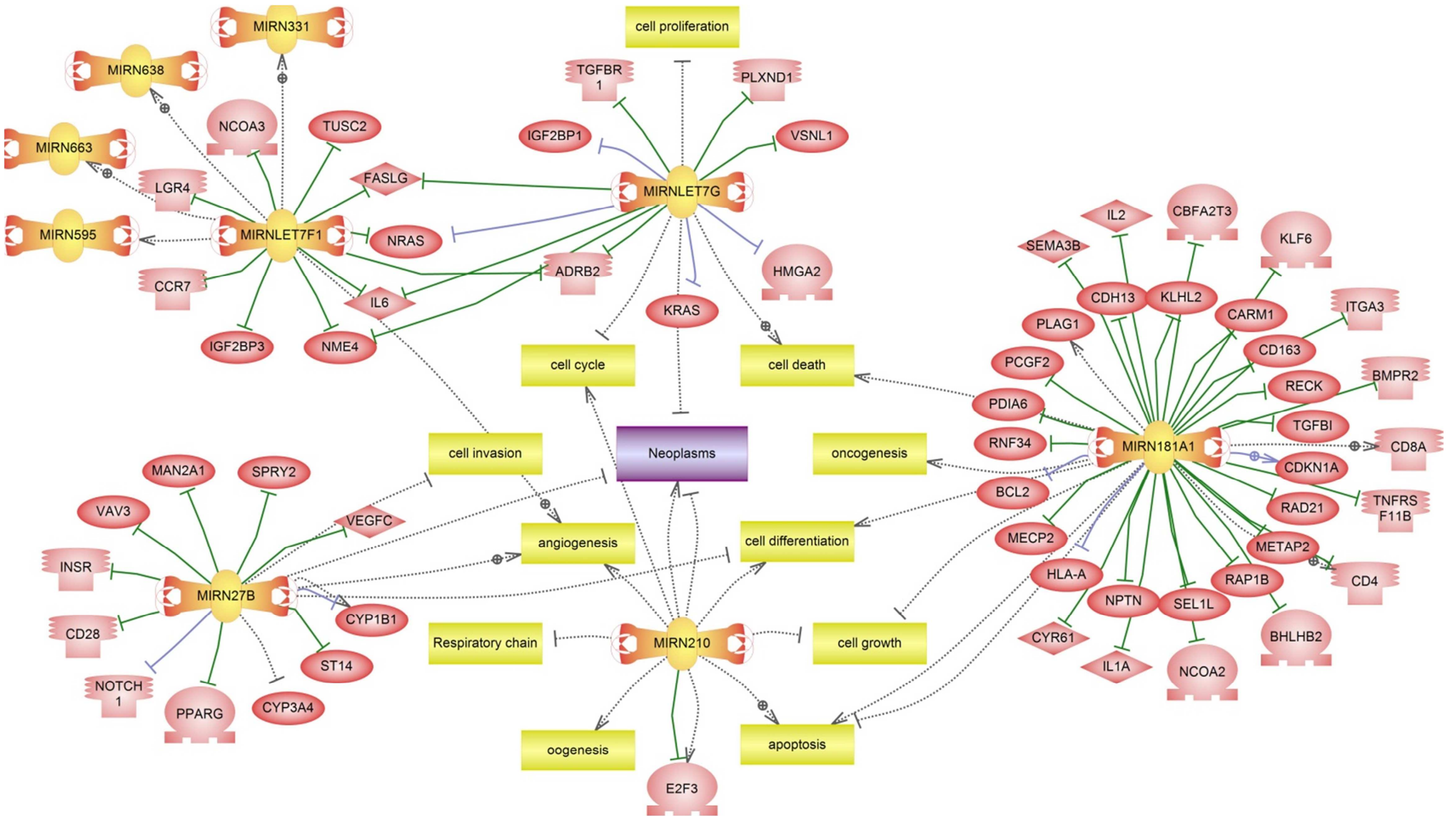

Pathway analysis of five miRNAs out of nine intersection miRNAs in SKBR3 that are linked to neoplasms, metastasis or carcinogenesis.

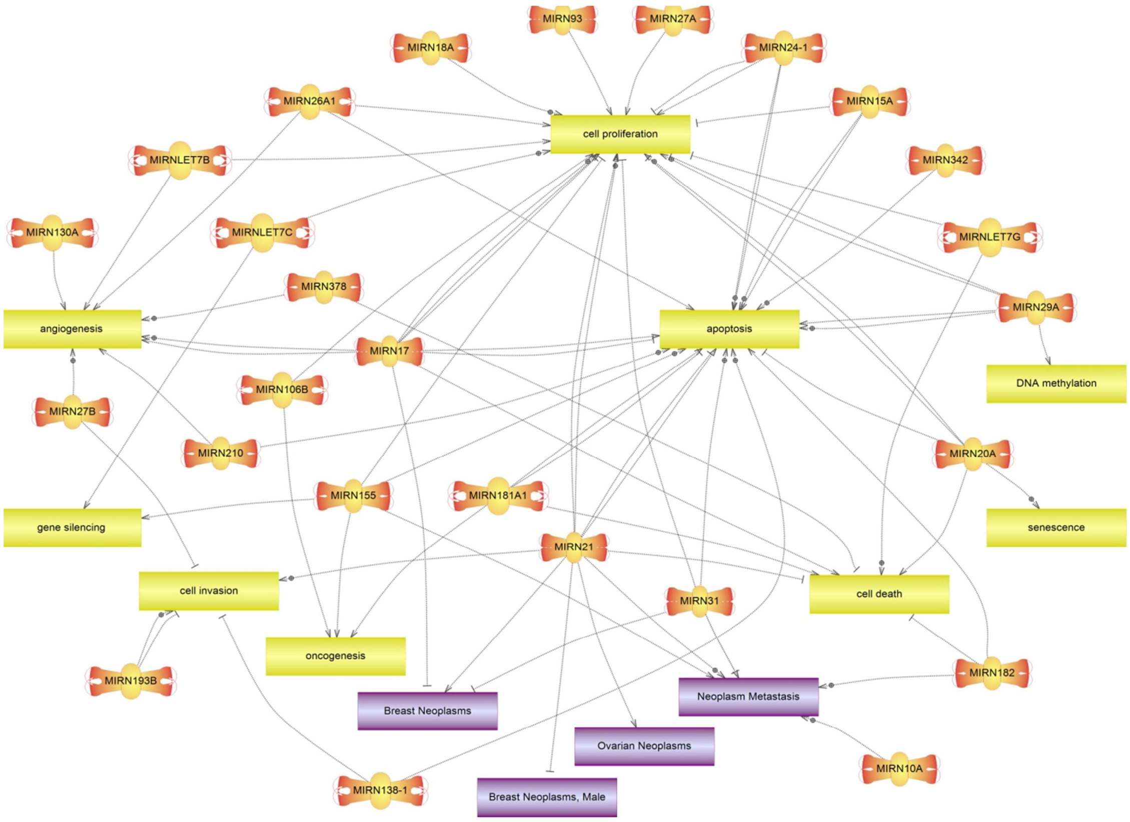

Pathway analysis of 27 union miRNAs in SKBR3 that are linked to neoplasms, metastasis or carcinogenesis.

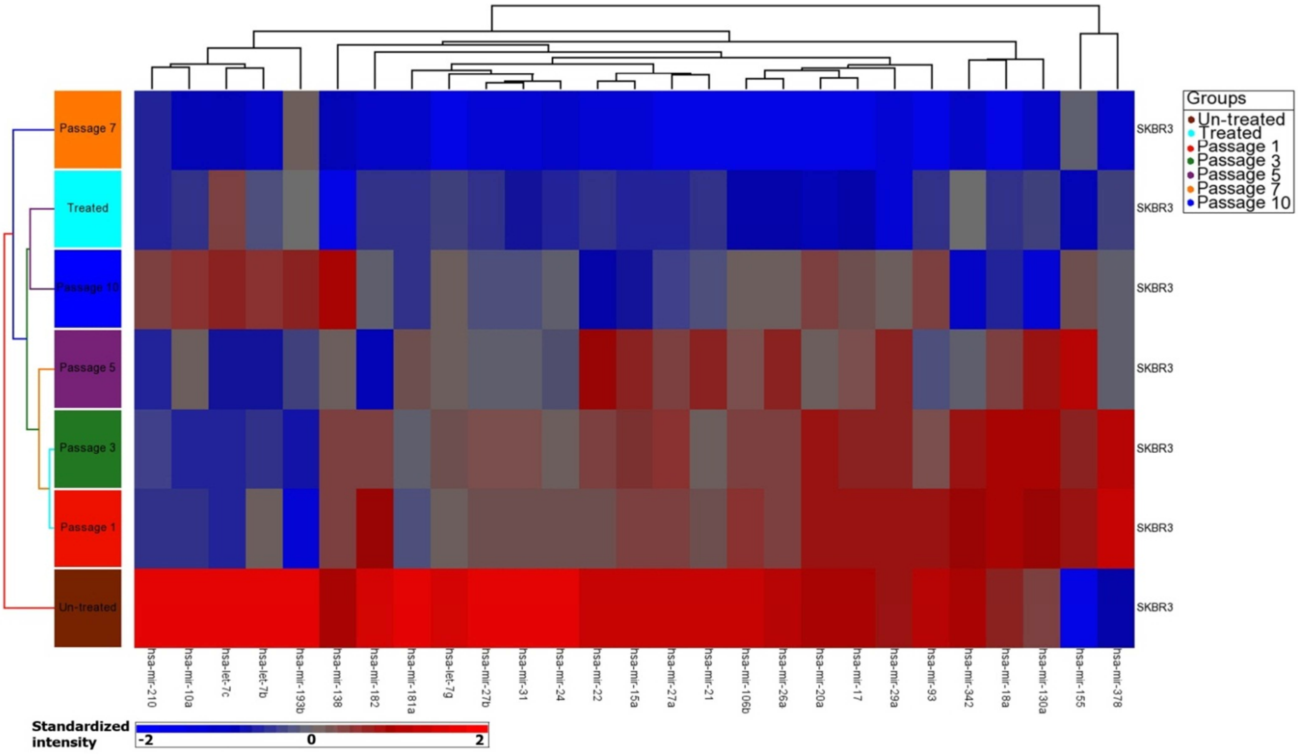

Heatmap shows 27 significant up/down-regulated union miRNAs in SKBR3.

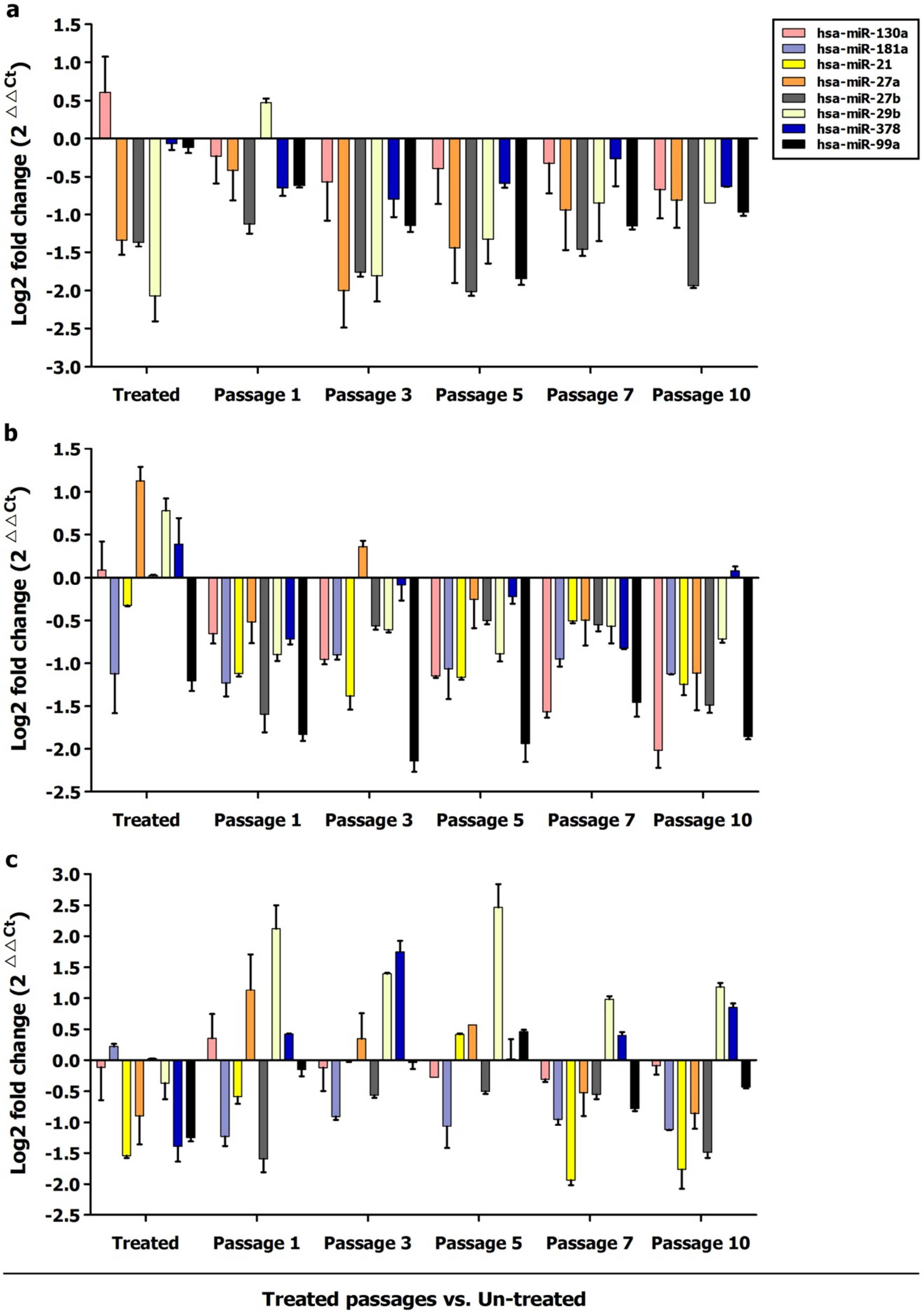

Expression level of important cancer related miRNAs using qPCR.

a) HB2 cell line. b) MDA-MB231 cell line. c) SKBR3 cell line.
