# Supplementary material for: Integrated Epigenetics of Human Breast Cancer: Synoptic Investigation of Targeted Genes, MicroRNAs and Proteins upon Demethylation Treatment
Source: PLoS One. 2011 Nov 4;6(11):e27355. doi: 10.1371/journal.pone.0027355 (PMC3208622; doi:10.1371/journal.pone.0027355)
Supplement: Dataset S4 — Proteomic Profiles. Spot picking gels for significant up/down-regulated intersection proteinsExpression profile of candidate spots within different treatment passagesPathway analysis of proteins/isoforms in that are linked to neoplasms, metastasis or carcinogenesisExpression profiles of five genes/proteins that were detected similarly in both transcriptomic and proteomic analysis (PDF) [file pone.0027355.s004.pdf]

Dataset S4

Proteomics Profiles

HB2 (Breast Epithelial Cell Line)

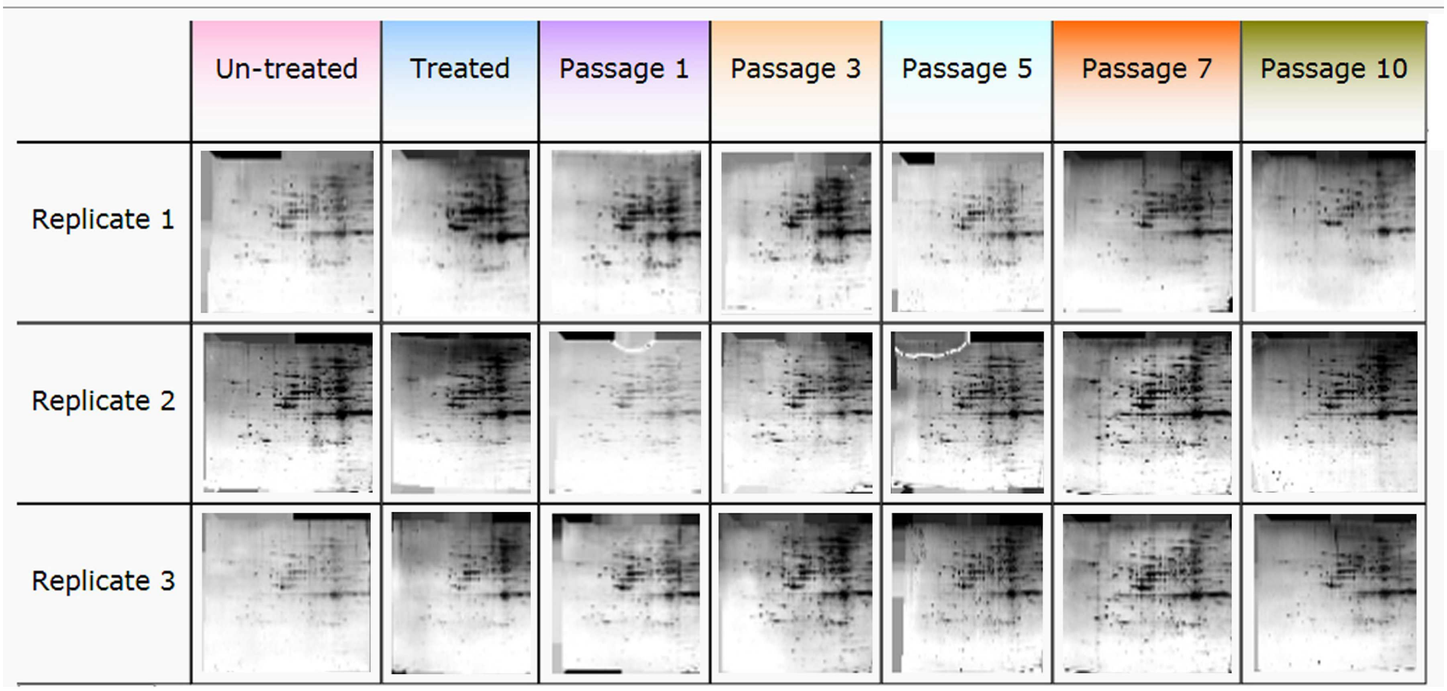

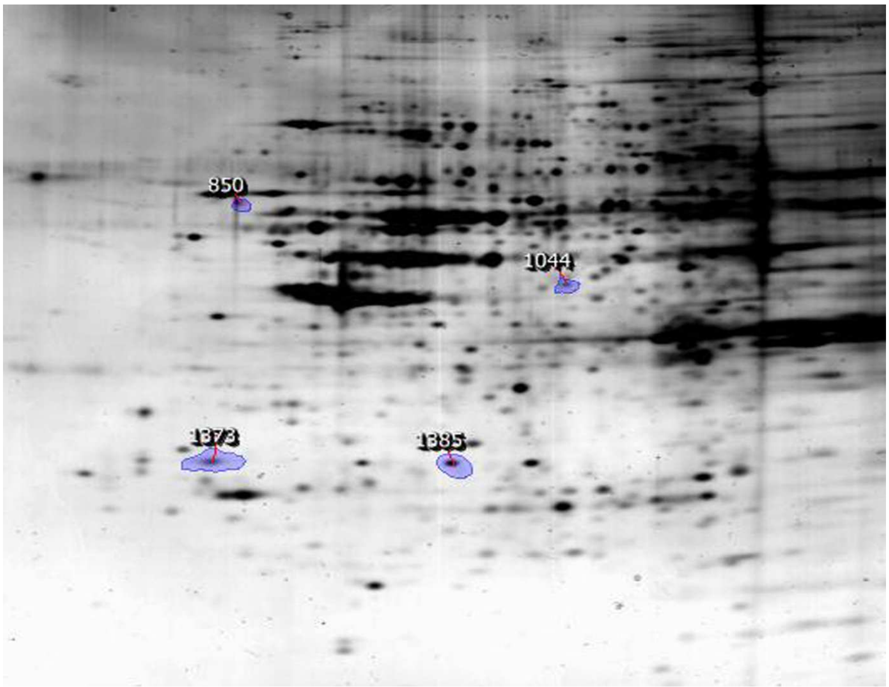

Spot No.	Anova (p)	Fold change	pI
1373	0.022	4.9	5.07
850	0.040	2.0	5.3
1044	0.044	1.6	7.75
1385	0.050	2.4	6.94

Spot picking gel for significant up/down-regulated intersection proteins.

Expression profile of candidate spots within different treatment passages.

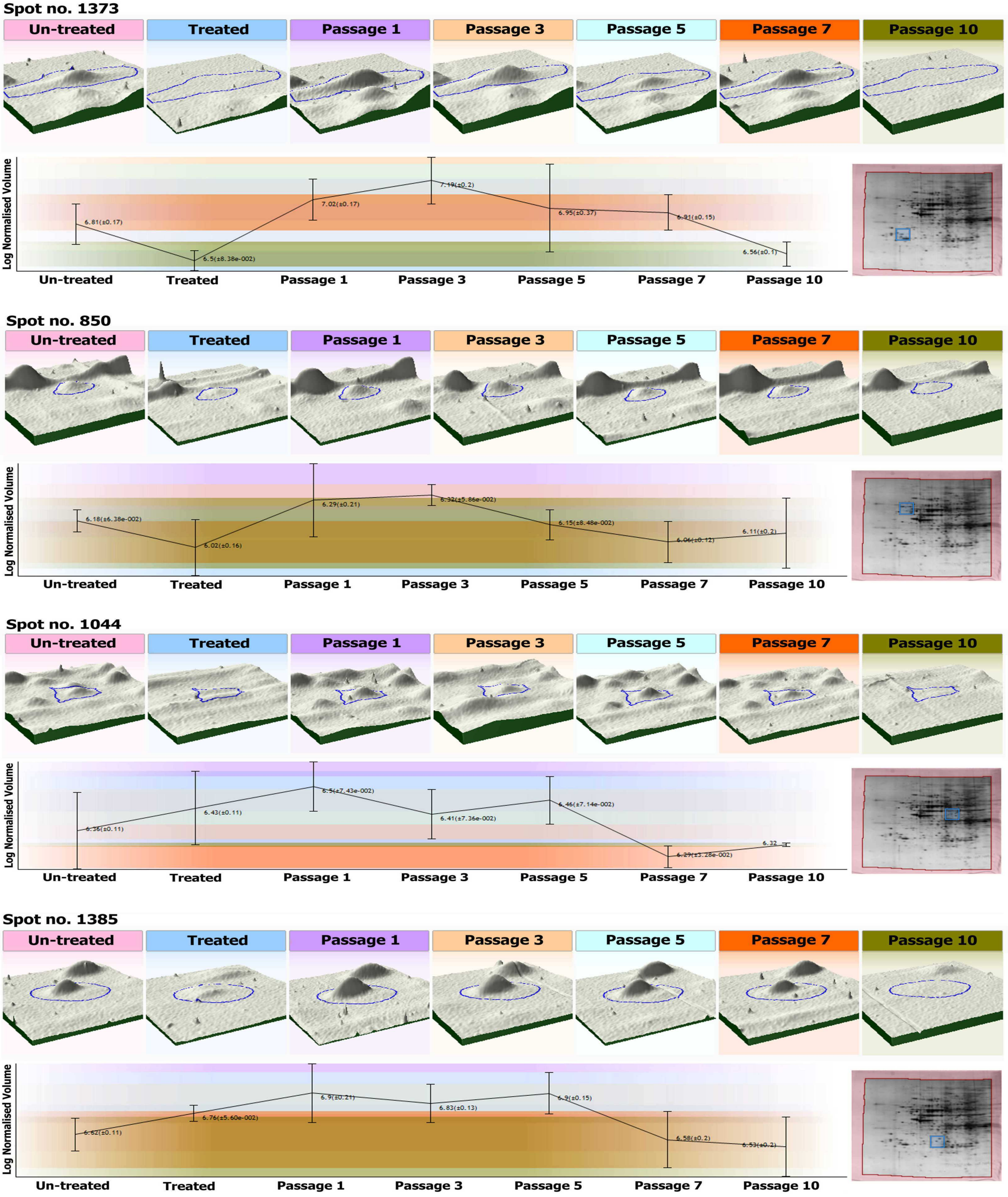

MDA-MB231 (Highly Aggressive Breast Cancer Cell Line)

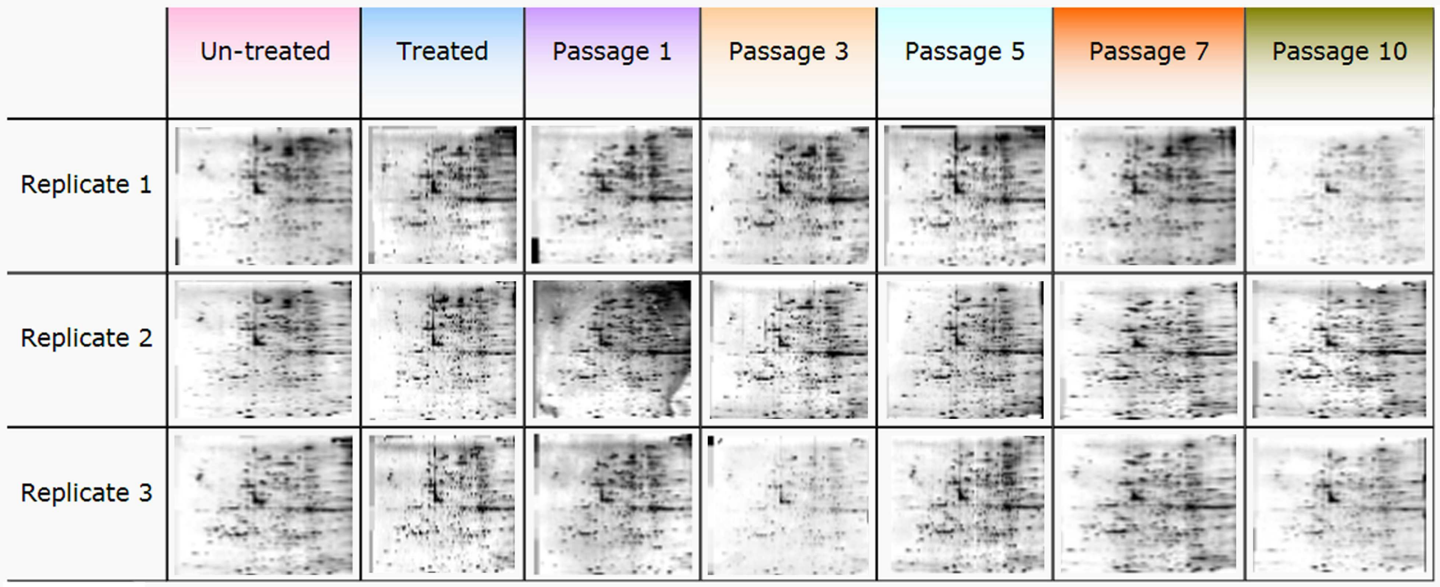

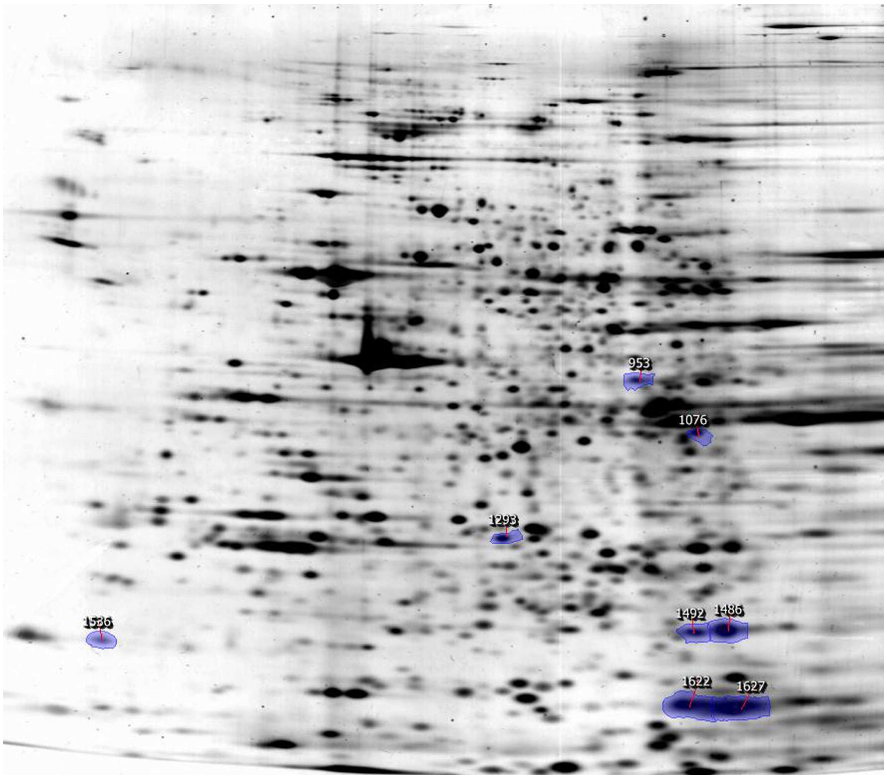

Spot No.	Anova (p)	Fold change	pI
1622	0.007	2.8	8.36
953	0.030	1.6	7.98
1486	0.032	1.9	8.7
1492	0.036	2.0	8.46
1627	0.038	2.6	8.83
1536	0.038	1.6	3.72
1293	0.041	1.7	6.94
1076	0.047	1.9	8.47

Spot picking gel for significant up/down-regulated intersection proteins.

Expression profile of candidate spots within different treatment passages.

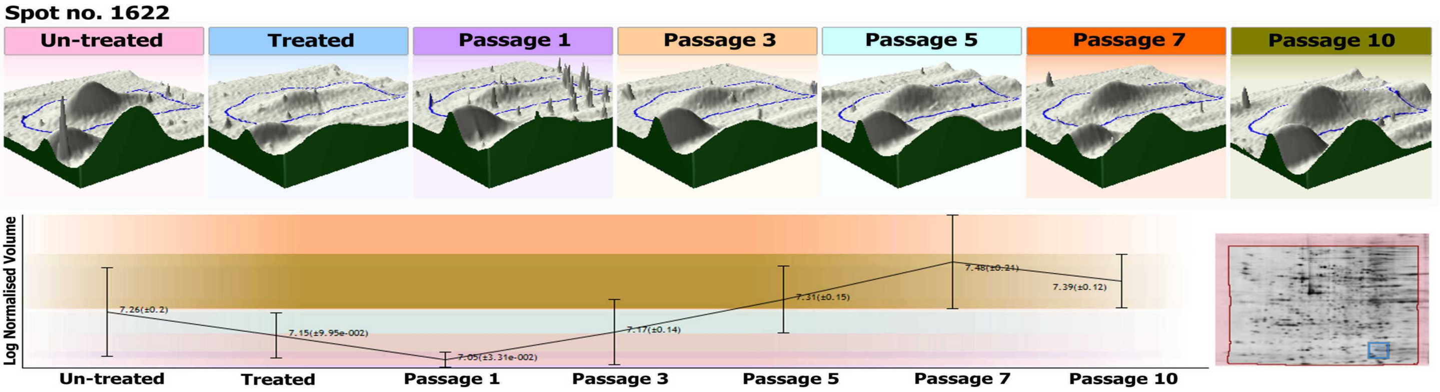

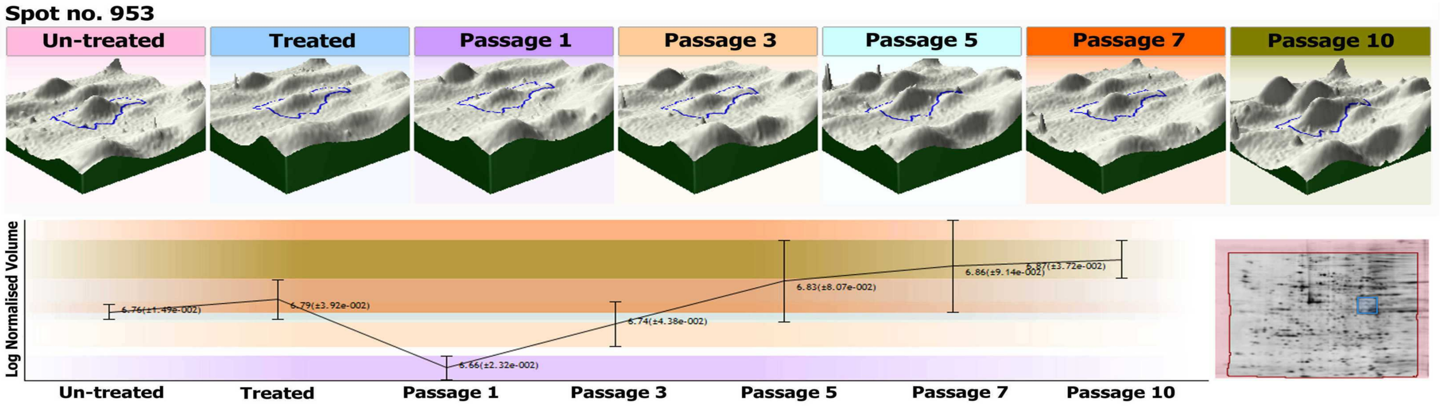

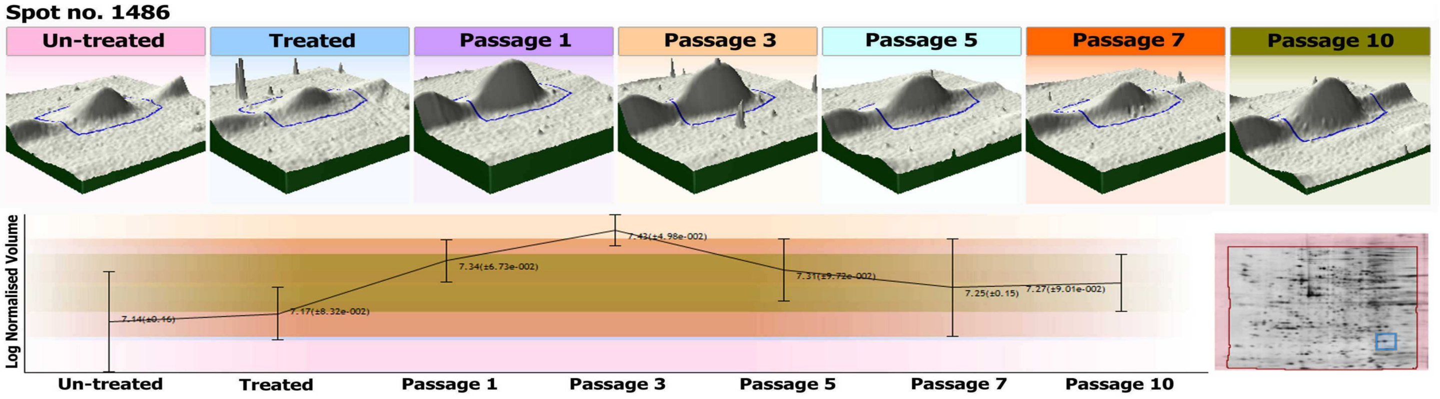

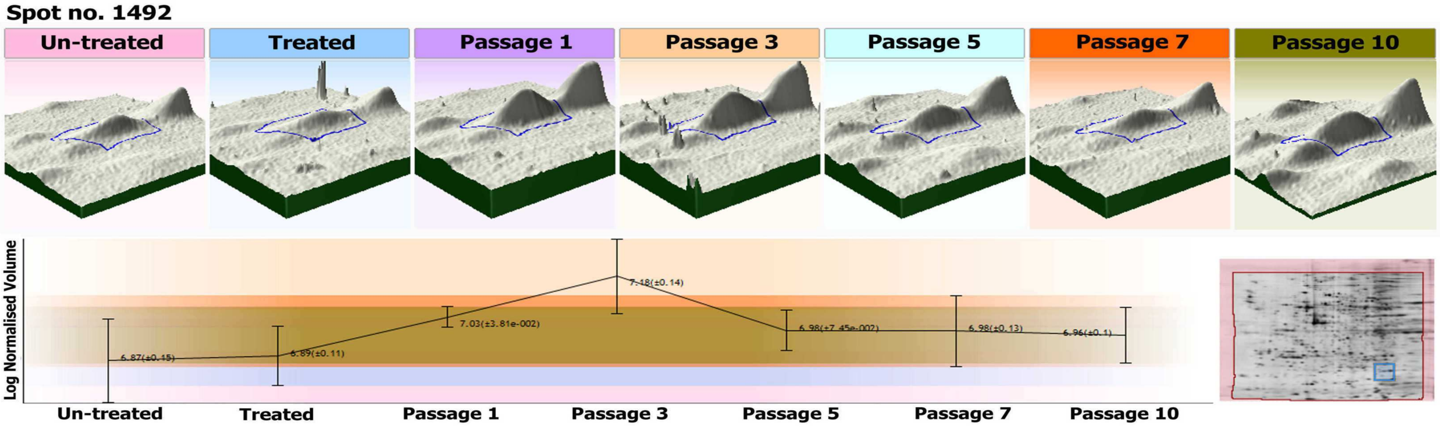

Spot no. 1627

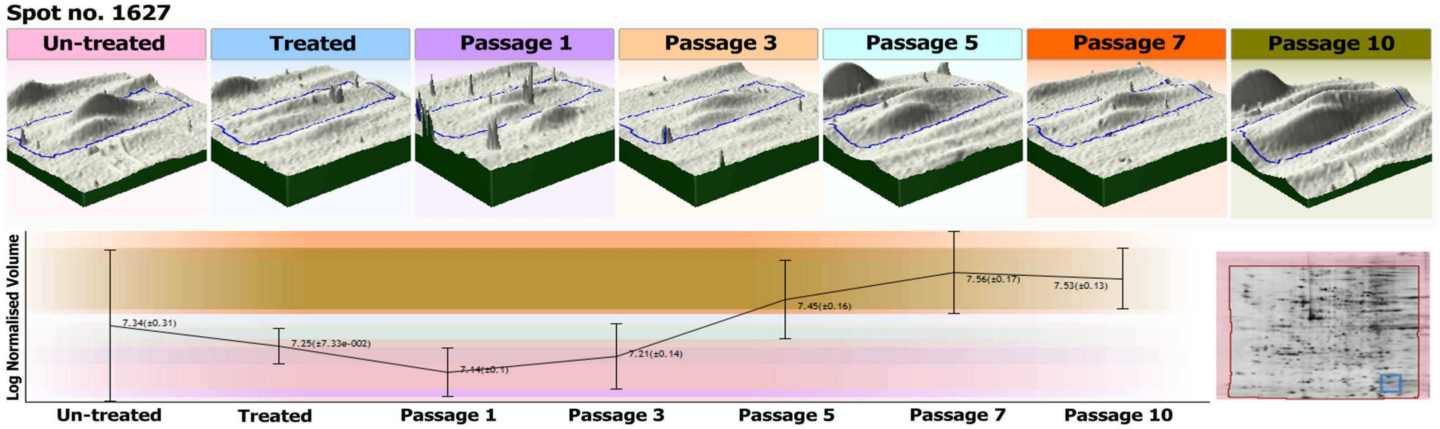

Spot no. 1536

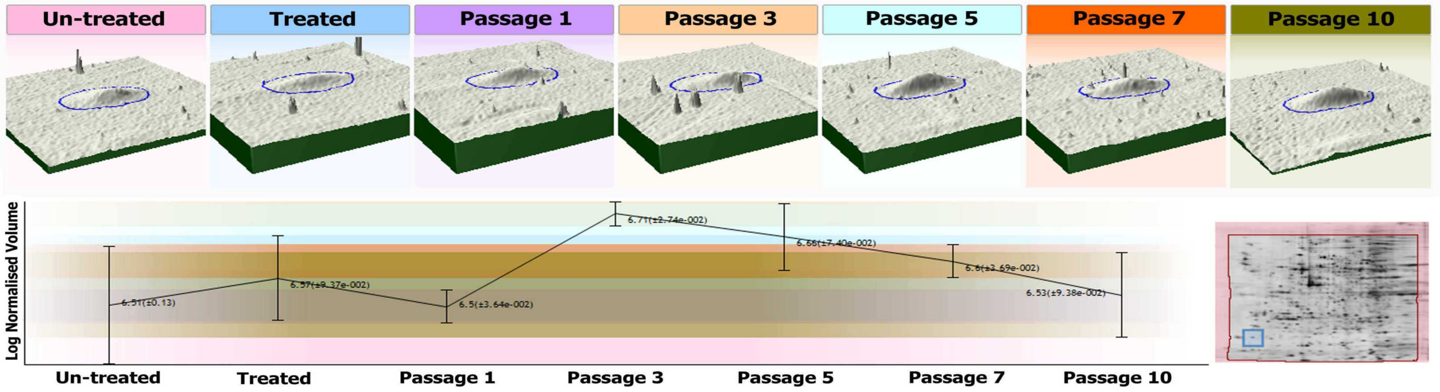

Spot no. 1293

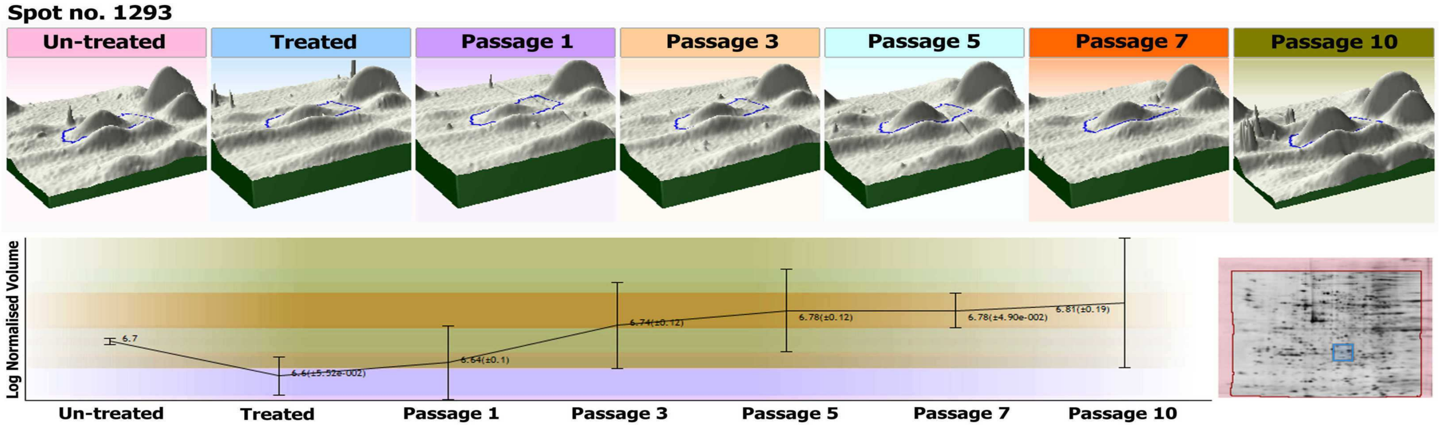

Spot no. 1076

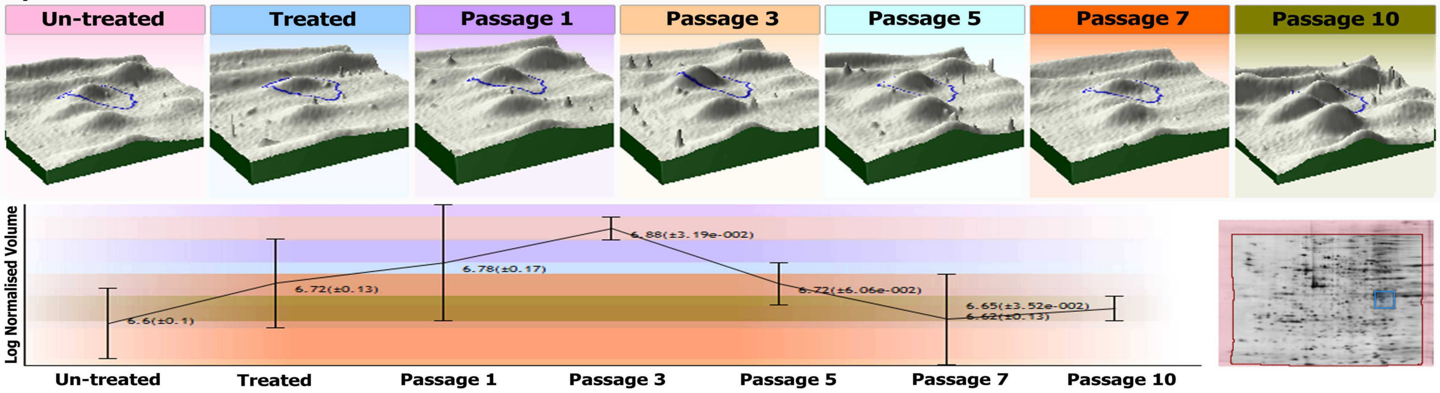

SKBR3 (Non- aggressive Breast Cancer Cell Line)

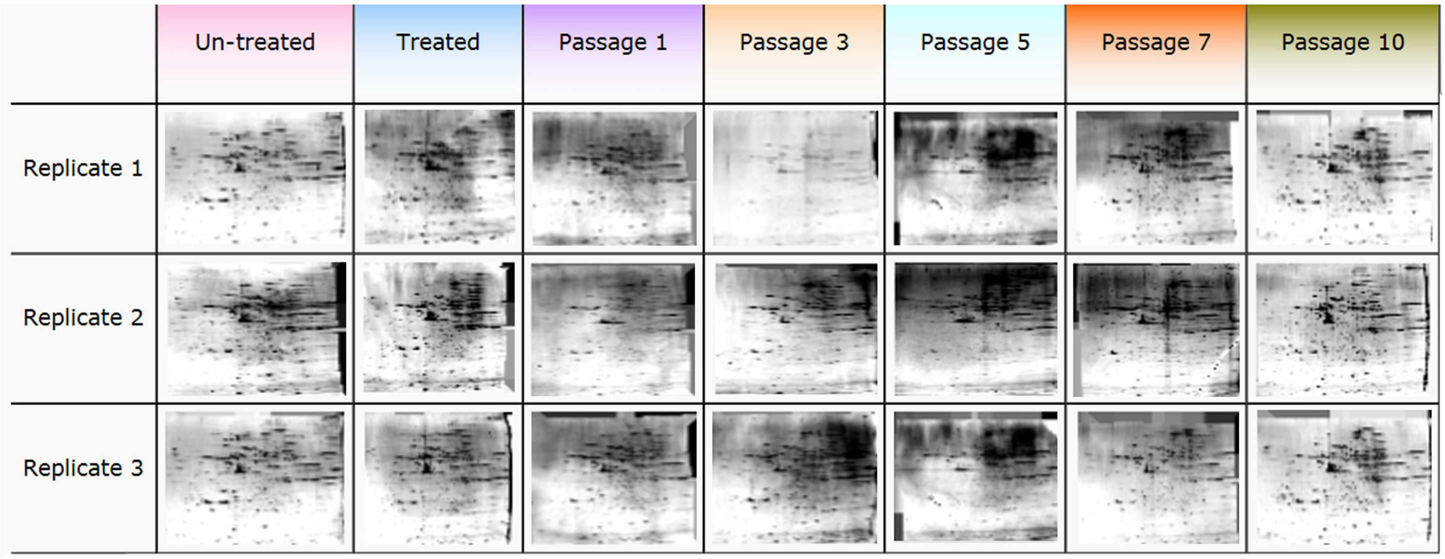

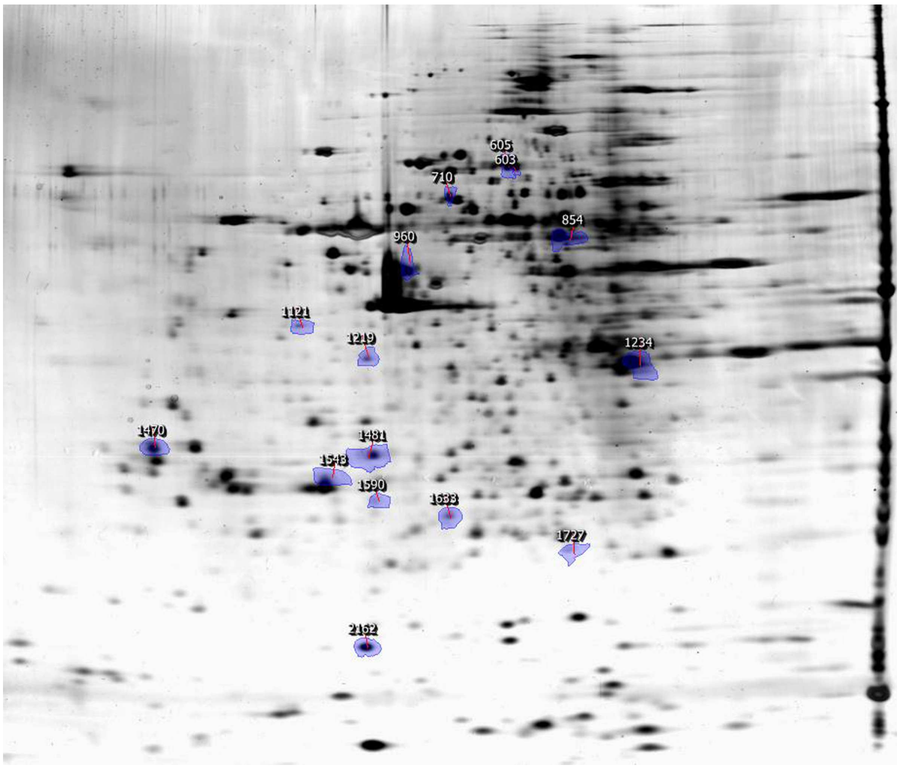

Spots No.	Anova (p)	Fold change	pI
710	0.005	3.1	7.2
1543	0.013	2.2	6.11
2162	0.016	2.6	6.42
1727	0.016	1.9	8.24
603	0.017	7.3	7.76
1470	0.021	6.6	4.67
1121	0.025	3.2	5.87
854	0.030	1.9	8.16
1633	0.030	2.9	7.18
960	0.038	2.7	6.8
1219	0.041	2.2	6.47
1234	0.045	2.0	8.78
605	0.047	3.3	7.7
1590	0.047	2.5	6.53
1481	0.048	2.7	6.53

Spot picking gel for significant up/down-regulated intersection proteins.

xpression profile of candidate spots within different treatment passages.

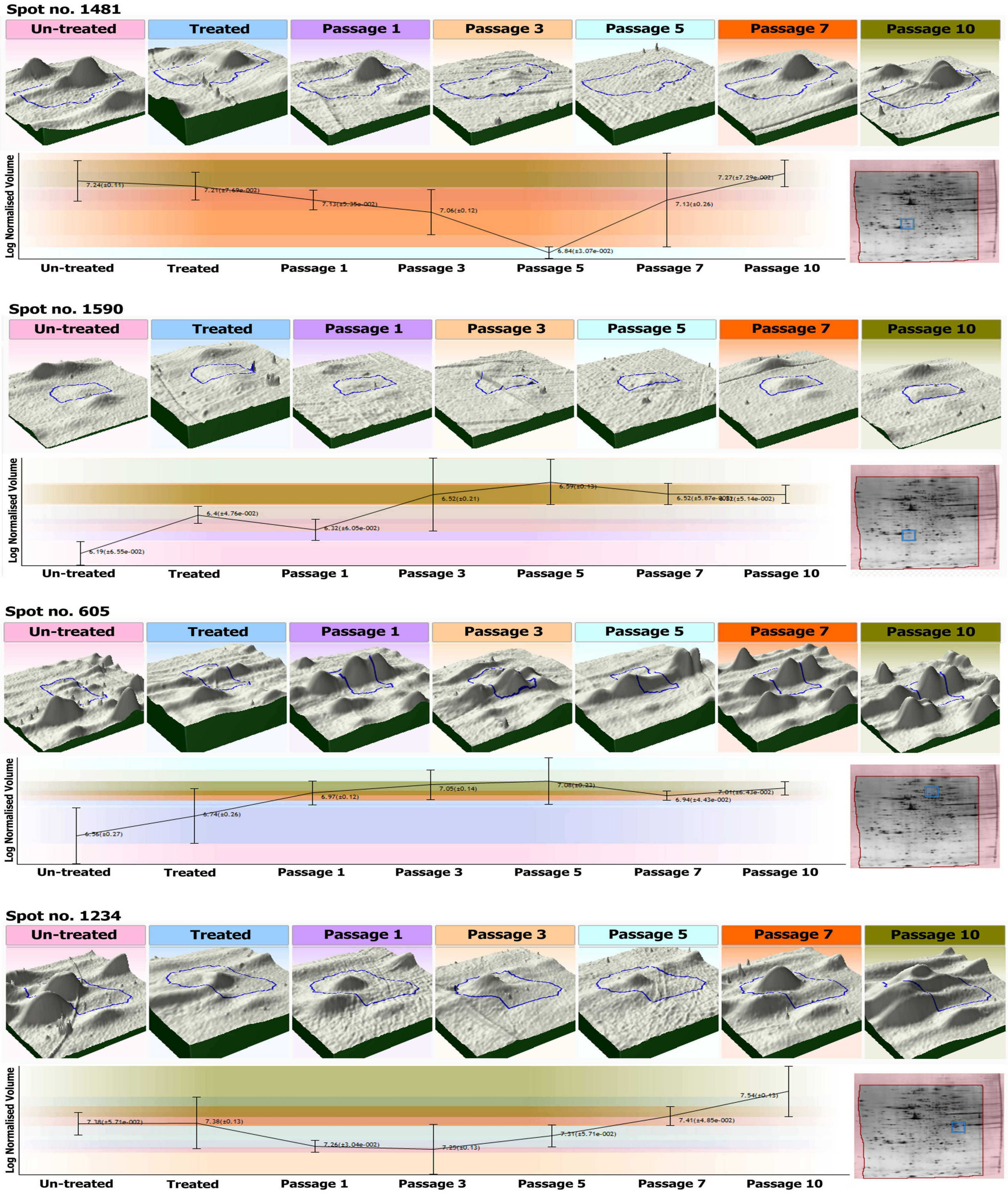

Spot no. 1219

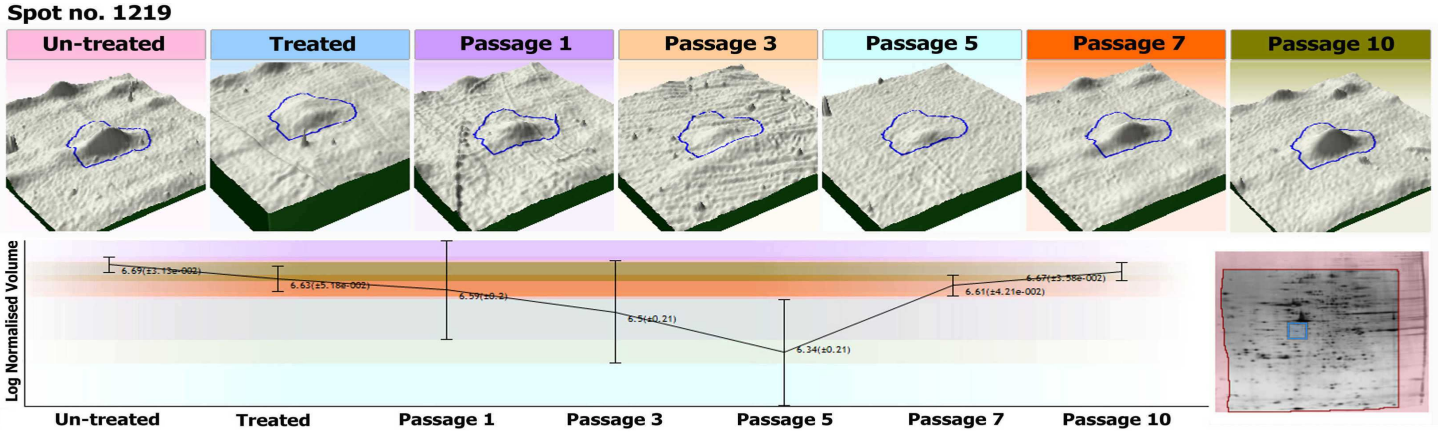

Spot no. 960

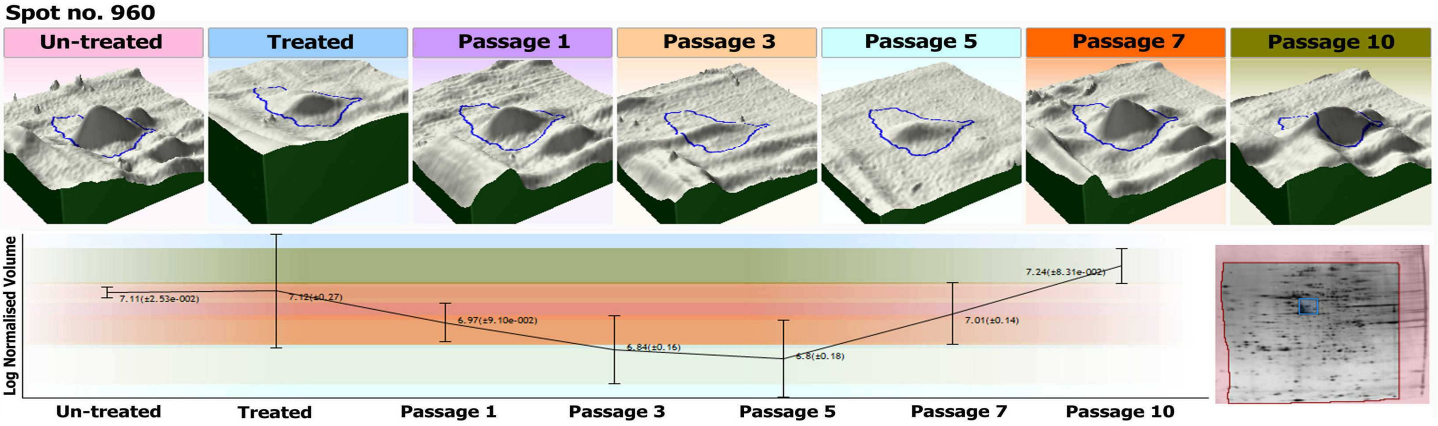

Spot no. 1633

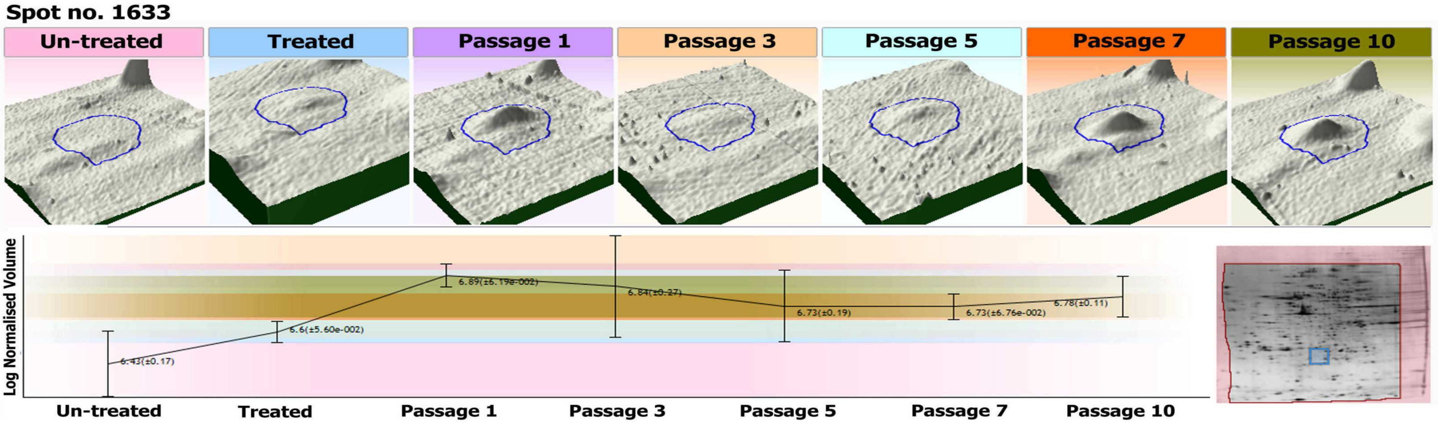

Spot no. 854

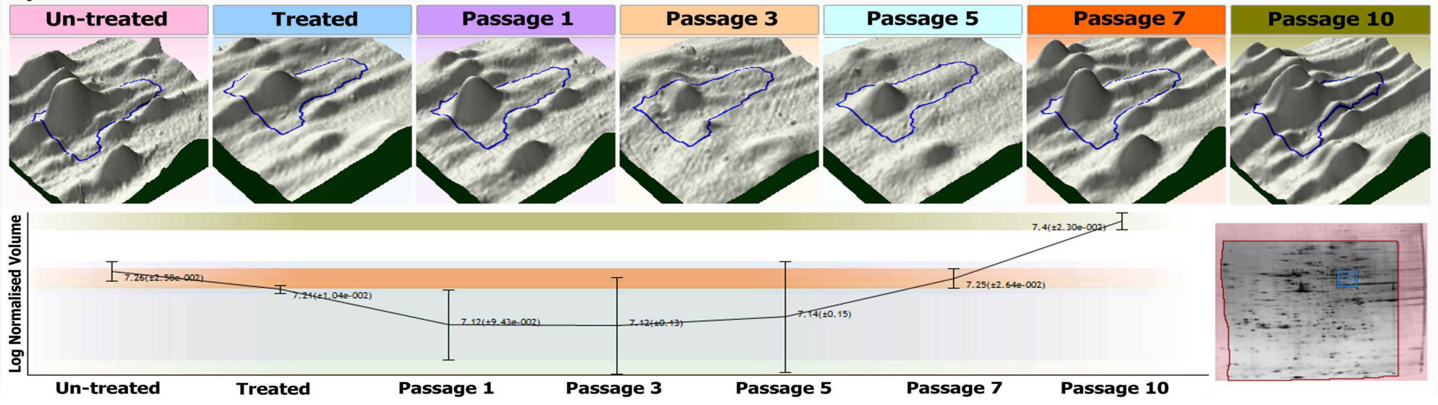

Spot no. 1121

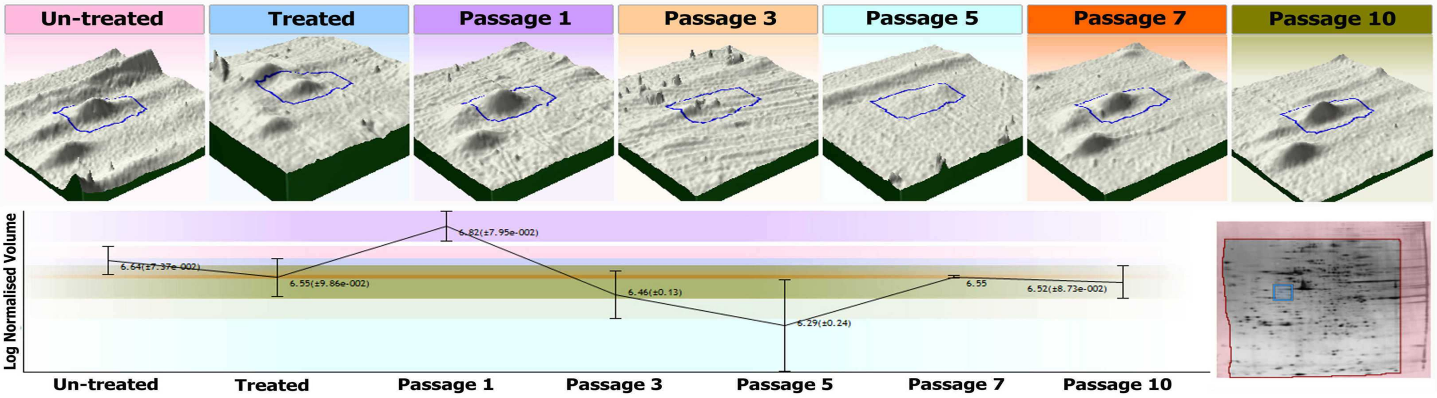

Spot no. 4070

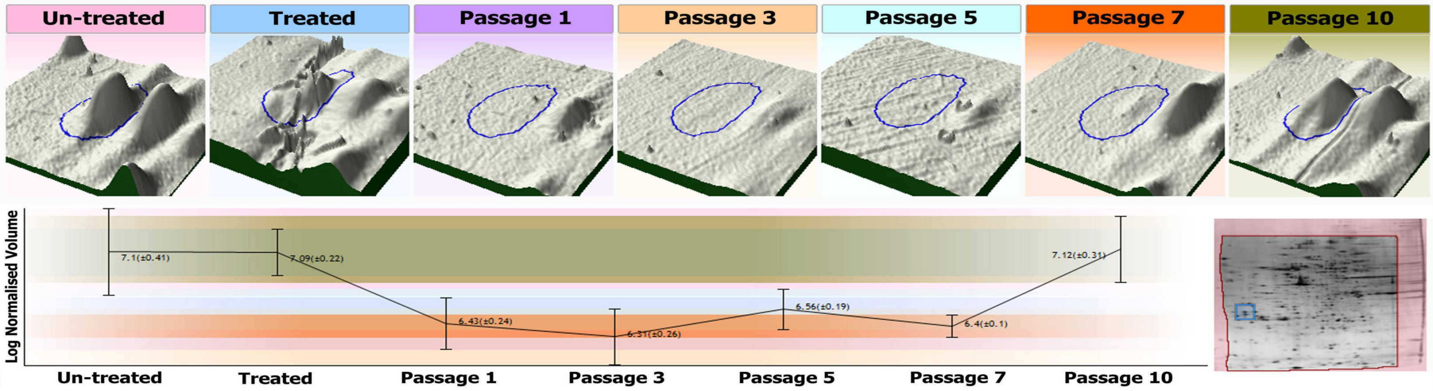

Spot no. 603

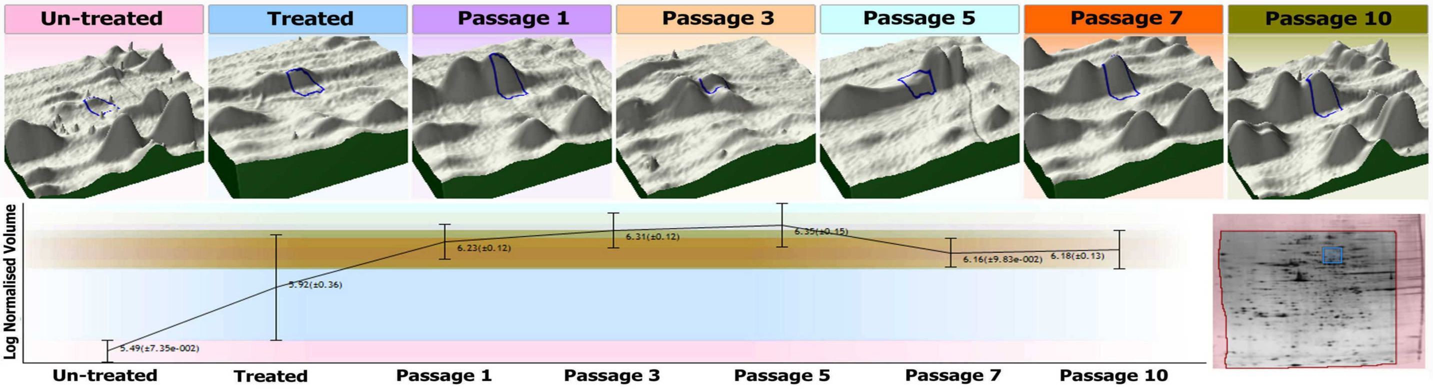

Spot no. 1727

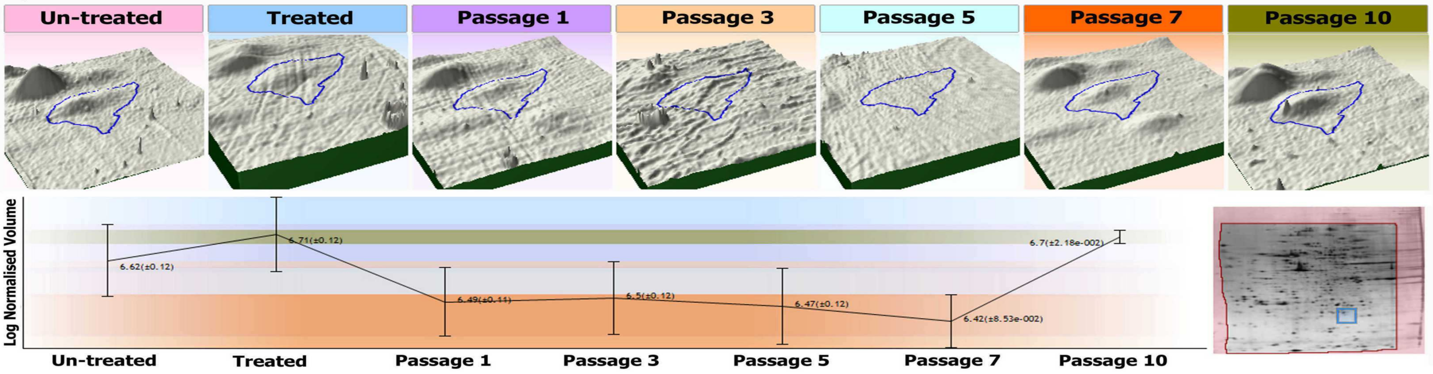

Spot no. 2162

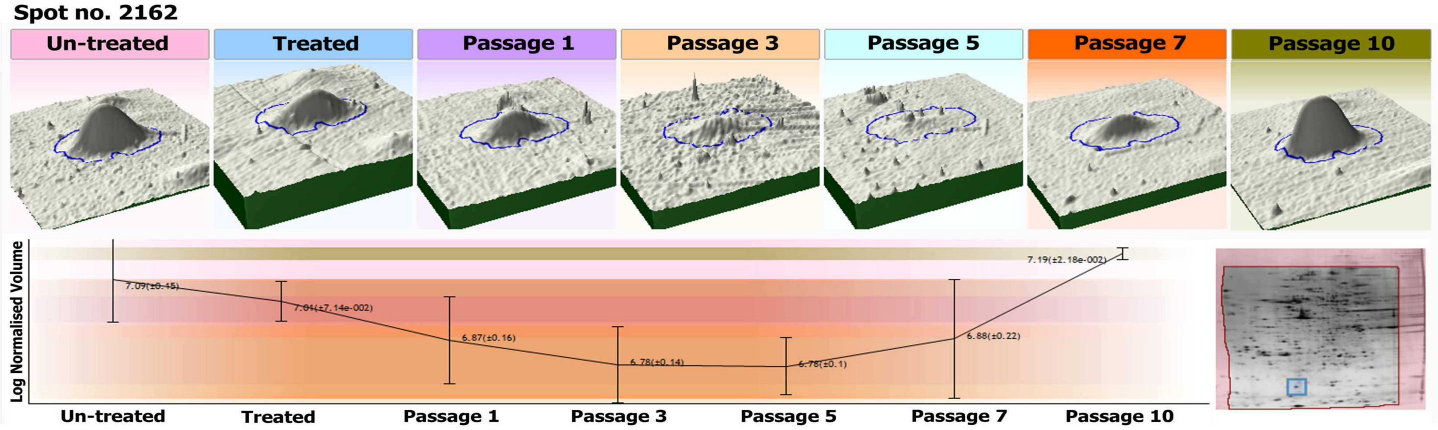

Spot no. 1543

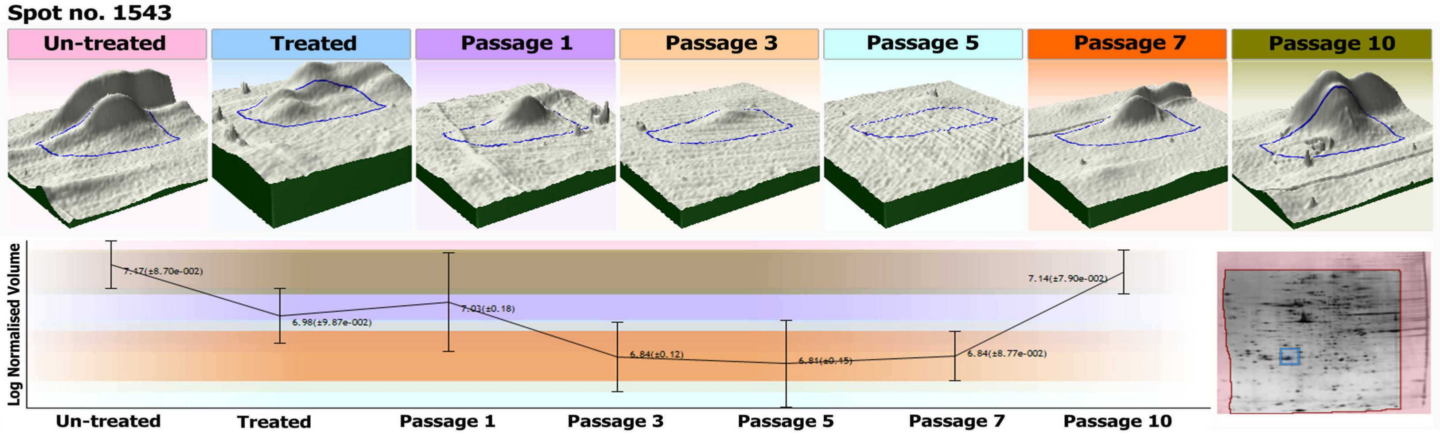

Spot no. 710

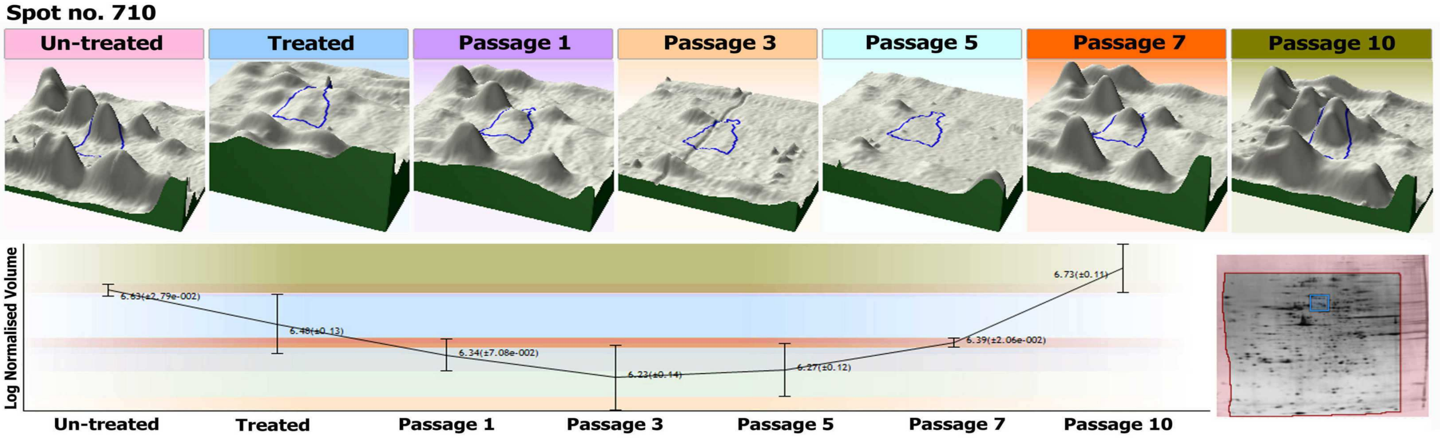

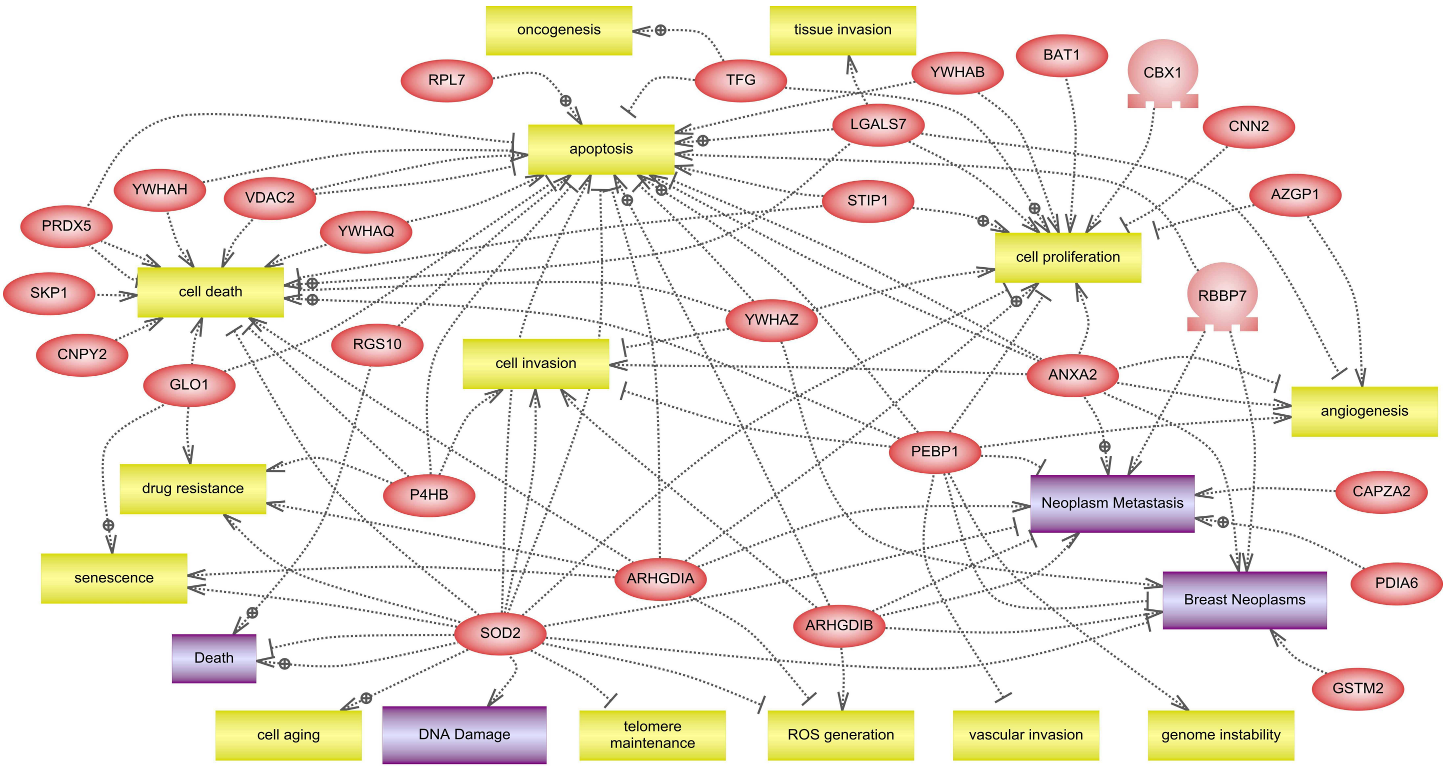

Pathway analysis of 28 out of 41 detected proteins/ isoforms in three analyzed cell lines (HB2, MDA-MB231, SKBR3) that are linked to neoplasms, metastasis or carcinogenesis.

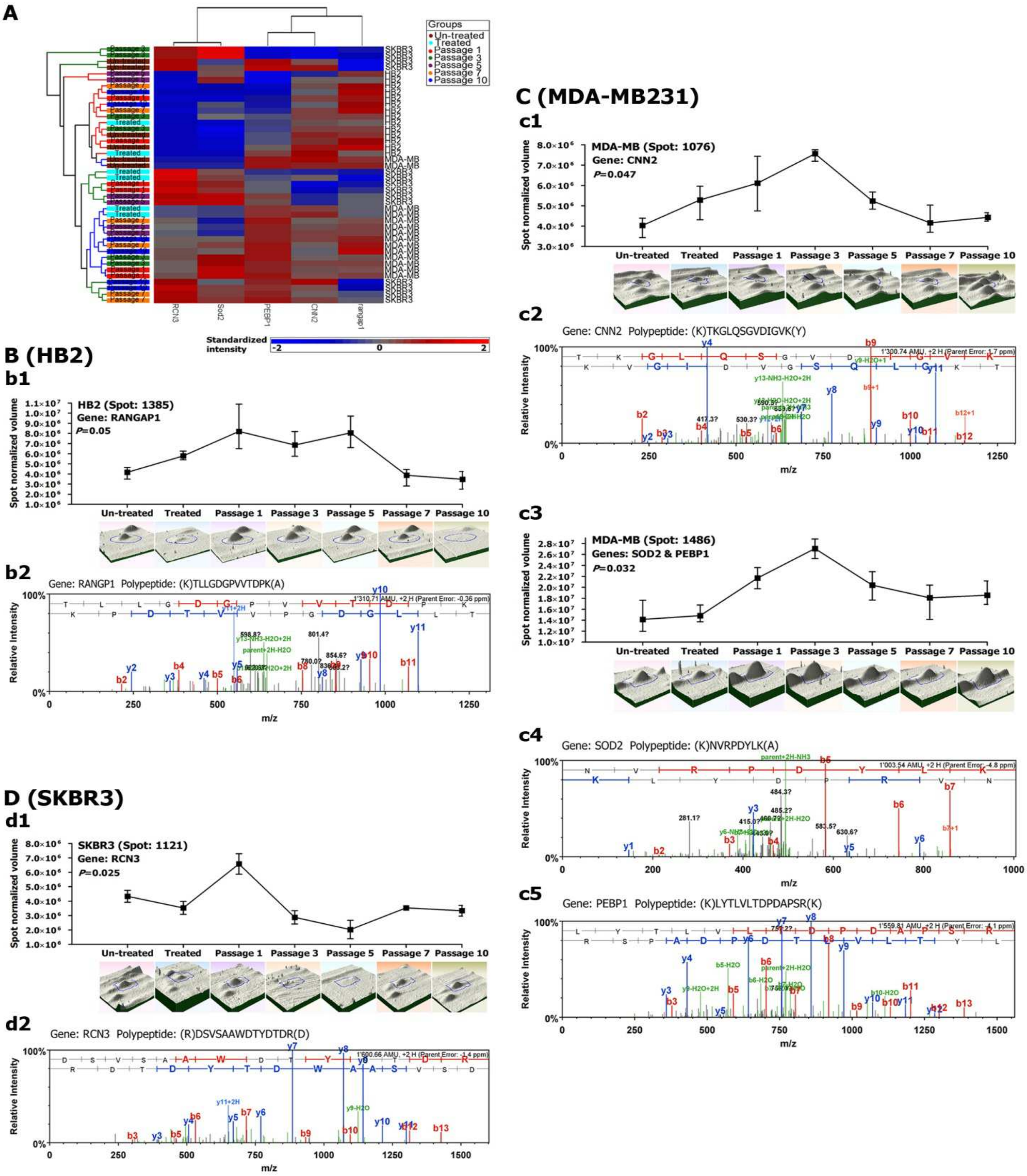

Expression profiles of five genes/proteins that were detected similarly in both transcriptomic and proteomic analysis. A) Expression profiles of five differentially expressed genes in transcriptomic analysis. B-D) Expression profiles of five proteins as significant changing markers in proteomic analysis for the HB2, MDA-MB231 and SKBR3 cell lines. b1-2) Proteomic profile of Rangp1 protein including expression pattern and LC/MS/MS detection. c1-2) Proteomic profile of Cnn2 protein including expression pattern and LC/MS/MS detection. c3-5) Proteomic profiles of Sod2 and Pebp1 proteins including expression pattern and LC/MS/MS detection. d1-2) Proteomic profile of Rnc3 protein including expression pattern and LC/MS/MS detection.
